# Replacing Plastics with Alternatives Is Worse for Greenhouse Gas Emissions in Most Cases

**DOI:** 10.1021/acs.est.3c05191

**Published:** 2024-01-31

**Authors:** Fanran Meng, Miguel Brandão, Jonathan M Cullen

**Affiliations:** † Department of Chemical and Biological Engineering, University of Sheffield, Mappin Street, Sheffield S1 3JD, United Kingdom; ‡ Department of Sustainable Development, Environmental Science and Engineering, KTH Royal Institute of Technology, Stockholm 100-44, Sweden; § Department of Engineering, University of Cambridge, Trumpington Street, Cambridge CB2 1PZ, United Kingdom

**Keywords:** Plastics, Greenhouse gas emission, Climate change, Life-cycle assessment, Plastic alternative, Plastic pollution

## Abstract

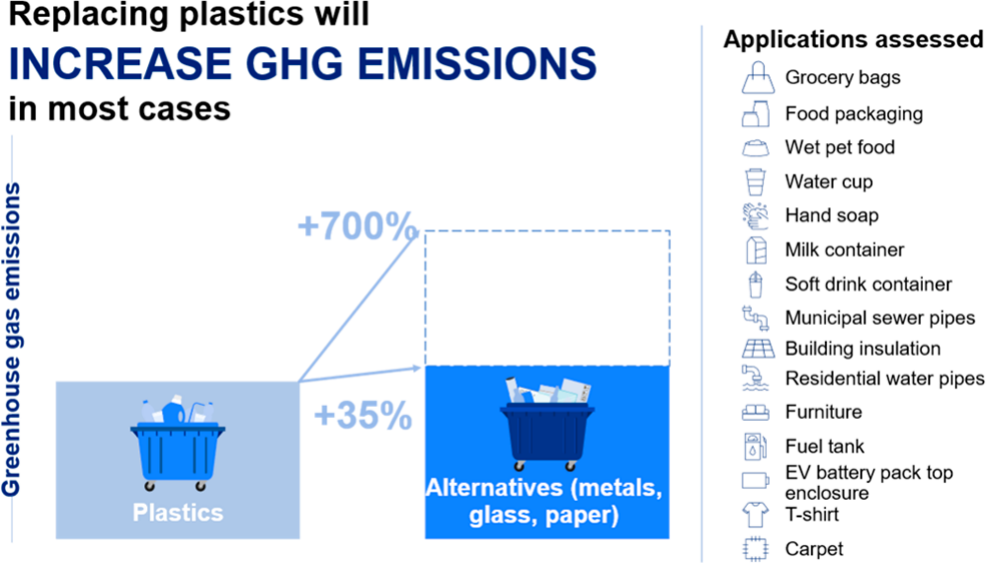

Plastics are controversial due to their production from fossil fuels, emissions during production and disposal, potential toxicity, and leakage to the environment. In light of these concerns, calls to use less plastic products and move toward nonplastic alternatives are common. However, these calls often overlook the environmental impacts of alternative materials. This article examines the greenhouse gas (GHG) emission impact of plastic products versus their alternatives. We assess 16 applications where plastics are used across five key sectors: packaging, building and construction, automotive, textiles, and consumer durables. These sectors account for about 90% of the global plastic volume. Our results show that in 15 of the 16 applications a plastic product incurs fewer GHG emissions than their alternatives. In these applications, plastic products release 10% to 90% fewer emissions across the product life cycle. Furthermore, in some applications, such as food packaging, no suitable alternatives to plastics exist. These results demonstrate that care must be taken when formulating policies or interventions to reduce plastic use so that we do not inadvertently drive a shift to nonplastic alternatives with higher GHG emissions. For most plastic products, increasing the efficiency of plastic use, extending the lifetime, boosting recycling rates, and improving waste collection would be more effective for reducing emissions.

## Abstract 2

Replacing plastics leads to higher full life-cycle emissions using alternative materials in most current applications.

## 1 Introduction

Plastic production, use, and disposal all emit significant amounts of greenhouse gases. Calls to use less plastics have garnered popular appeal in response to concerns about plastic pollution and climate change. However, if reducing plastic use requires a switch to alternative materials or products, then it is critical that these alternatives result in lower emissions. For example, what good is a shift away from plastic bags if the paper alternative emits more greenhouse gas (GHG) emissions across the product life cycle? Images of plastic bags clogging rivers and turtles ensnared by six-pack rings have become familiar viewing. Despite its many uses, plastics have attracted growing criticism for its role in marine pollution and roadside litter.^1−5^ Further controversy has arisen over plastics being derived from fossil fuel feedstocks. These are valid concerns. Between 1950 and 2015, annual plastic production increased from 2 to 380 Mt, with a cumulative 8500 Mt produced in that period.^6^ Geyer et al. estimated that 6300 Mt of this plastic production was discarded as waste, but only 600 Mt were recycled. The remaining plastic waste was either incinerated (∼800 Mt) or deposited in a landfill or the natural environment (∼4900 Mt).^6^ Better disposal of plastics is an urgent challenge for the plastic industry and governments, given the threats to biodiversity and ecosystem health worldwide.^1,7^

The contribution of plastics to the greenhouse effect is a less commonly emphasized but still a pressing concern. Plastics are responsible for approximately 4.5% of global GHG emissions.^8^ International commitments to keep global warming to within 1.5 °C above preindustrial levels, therefore, require urgent actions to address the climate impacts of plastics.^9^ Some such actions are being taken. In January 2023, the UK government announced a ban on single-use plastics, and several other countries have banned the use of plastic bags and straws.^10^

However, the wider environmental implications of a shift away from plastics and the substitution of alternative materials, such as paper, glass, or metal, have received little attention. A balanced, science-based perspective will be required to reduce GHG emissions, while still pursuing other objectives, such as minimizing waste leakage^11^ and promoting circular systems.^12^ Plastic alternatives are typically heavier and therefore incur more emissions during production and use, while biodegradable alternatives can release more emissions during end-of-life treatment. There remains a lack of academic studies that compare the full life-cycle impacts of plastic products against their alternatives across the full range of products in use.

In this context, life-cycle assessment (LCA) is a useful method for assessing the environmental impact of comparable products, and this methodology can easily be applied to plastic products and systems.^13^ This paper adopts an LCA approach to assess the GHG emissions of plastic products versus alternative products in the same market applications. The goal is to assess the climate change impacts of plastics across a broad range of applications with enough rigor to be representative, comprehensive, and meaningful. In doing so, this work provides an additional perspective to the plastics sustainability dialogue through the lens of life-cycle GHG emissions, providing context on the alternatives available and offering science-based arguments to guide future discussions. We demonstrate the significant complexity within each plastic use sector and uncertainties associated with key parameters, including emissions levels and end of life (EoL) treatment options.

## 2 Methods

Following ISO14040/44 standards,^14,15^ we develop full life-cycle models to assess the total direct and indirect GHG emissions from plastics and alternative materials in 16 applications: 14 with nonplastic alternatives and two with plastic-enabled mix alternatives. Applications are selected judiciously to cover the full spectrum of plastic use, covering about 90% of global plastics by volume (see Section 2.5). Our base analysis focuses on the United States in 2020, with sensitivity analyses extending to other geographical regions, such as western Europe and China, and creating a 2050 view of a decarbonized and circular world. The decision to base our analyses on the United States stems from the availability of data and the fact that the US’s energy mix and EoL treatment options are close to the global average.^16^ We leverage the US EPA’s Waste Reduction Model^17^ as our primary life-cycle inventory data source, augmented with data from the ecoinvent database v3.7^18^ and other published LCA studies. Details of direct and indirect impacts, as well as the sources and expanded allocations for each application, can be found in the Supporting Information.

### 2.1 Functional Unit

A functional unit is the quantified performance of a product system used as a reference in an LCA. For example, a functional unit for a beverage container can be defined as a given volume of the beverage. Product-level comparisons within each application are chosen to be fair and reasonable using an appropriate functional unit that reflects equivalence between alternatives including the consideration of product life spans. For example, a 250 L (55 gallons) drum with a 10-year usage is chosen as the functional unit for the industrial drum, to neutralize the difference in life span between the high-density polyethylene (HDPE) drum with a 5-year life span and the steel drum with a 10-year life span (see Table S2, and further details can be found in Supporting Information). Impacts are allocated to coproducts based on reaction stoichiometry and production context, typically by mass. The substitution approach is used for recycling, whereby the substitution of virgin material production results in a credit at the EoL to reflect the avoided burdens.

### 2.2 System Boundary

**Figure 1:**
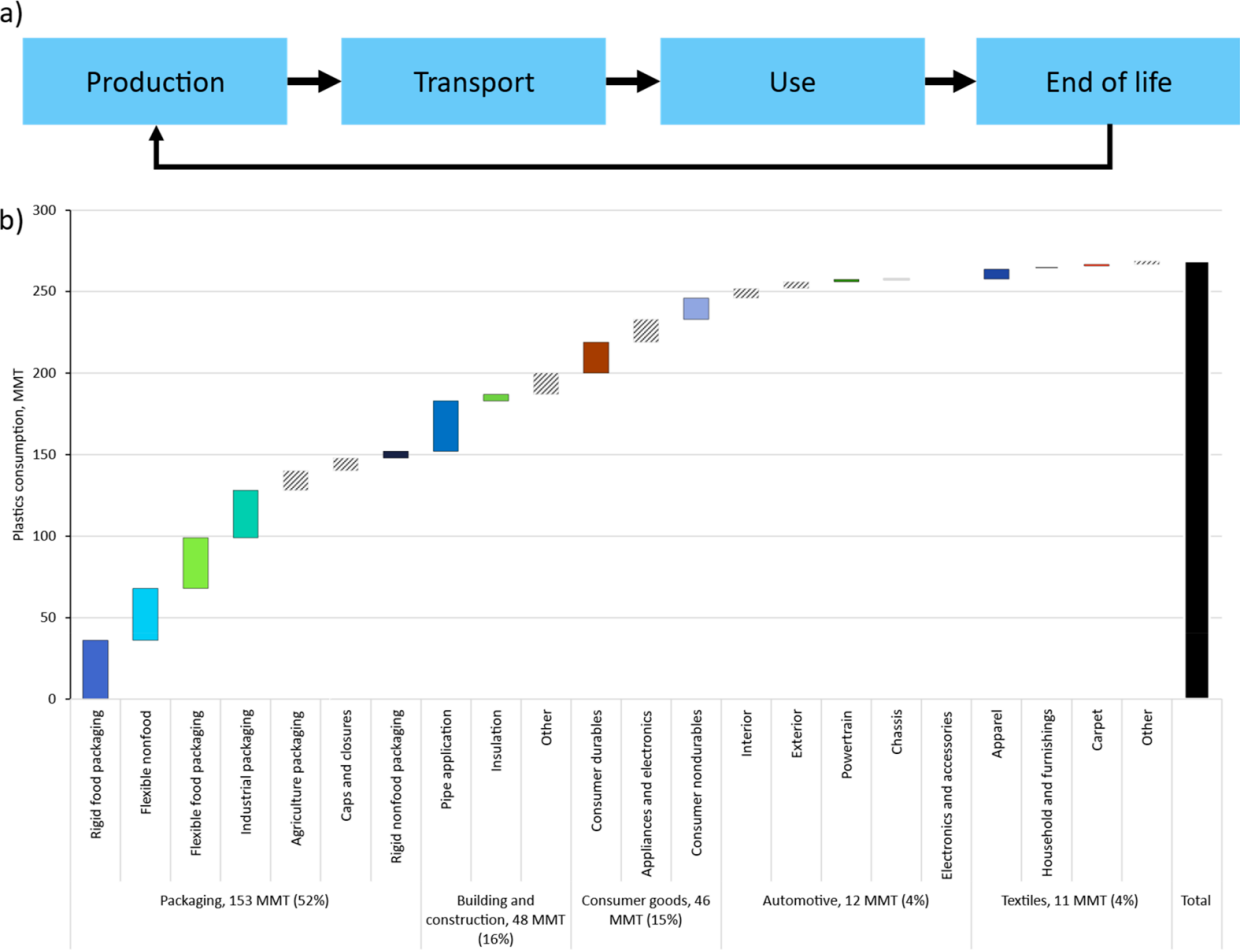
(a) Overview of the system boundaries. (b) 16 selected application categories based on the top five sectors for 2020 global plastic demand, million metric tons (MMT) (diagonal stripped application categories represent the applications not selected). WtE = waste to energy. Plastic applications cover about 90% of the global plastics by volume.

The system boundary is chosen on a cradle-to-grave basis (throughout the product’s life cycle) (Figure 1), with the following phases:

- Production includes emissions from resource extraction, raw materials processing, final product manufacturing, and all transportation steps including distribution.
- Transport emissions are calculated using the average distance traveled from product manufacturing facilities to retail outlets and mode-specific fuel used based on data obtained from the 2012 US Census Commodity Flow Survey.^19^ Transport from retail to end user is not included due to a lack of available data, and this is assumed to be a nonmaterial factor.
- Use includes emissions resulting from product breakage and spoilage, heating and cooling requirements from improved insulation, and fuel efficiency from light-weighting.
- End of life considers emissions based on four EoL pathways using a system expansion approach. The pathways are adopted in the model in proportions representative of their shares in the US and are as follows:
  - Landfill, including transport to landfill, methane emissions
  - Waste to energy (WtE), which refers to incineration with energy recovery and includes transport to the combustion site, combustion emissions, avoided utility emissions, and steel recovery offsets when the plastic alternatives are steel
  - Recycling, which includes collection, sorting, processing, and transport to a manufacturing facility that uses recycled inputs
  - Reuse, which includes collection, washing, and transport to a refilling facility

### 2.3 Life-Cycle Inventory

Life-cycle inventory data are collected from various publicly available data sets including the EPA’s Advancing Sustainable Materials Management report^20^ and various industry reports.^21^ The electricity grid mix factor is calculated based on the US Energy Information Administration’s Annual Energy Outlook^22^ and the EPA’s Inventory of US Greenhouse Gas Emissions and Sinks^23^ and the Emissions & Generation Resource Integrated Database.^24^ In addition, the model uses regional energy mix data from the International Energy Agency (IEA)^22^ and data from the McKinsey Centre for Future Mobility^25^ for the commercial internal combustion engine vehicle (ICEV) versus battery electric vehicle (BEV) mix for the transportation of goods. Expert interviews and industry reports are used to identify secondary emissions during the use phase (e.g., breakage, fuel efficiency, heating, etc.). Further details can be found in the Supporting Information.

### 2.4 Life-Cycle Greenhouse Gas Emissions

Models are created to assess the direct and indirect impacts for each of the applications. GHG emissions are calculated based on the most recent Integrated Pollution Prevention and Control 100-year Global Warming Potential factors in terms of CO_2_ equivalents (CO_2_e).^26^ The model includes (a) methane (CH_4_) and nitrous oxide (N_2_O) from landfill decomposition or waste-to-energy (WtE) processing of biogenic carbon and (b) methane from cellulose decomposition in landfill storage for alternative materials where this applies. Biogenic CO_2_ from landfills or WtE and carbon stored in fossil-derived products in landfills are excluded.

### 2.5 Plastic Product Use

Global plastic demand is disaggregated by sector and provides the basis for selecting applications for the analysis. In 2020, global plastic demand was approximately 300 million metric tons (MMT), of which the top five sectors with the highest plastic consumption—packaging, building and construction, consumer goods, automotive, and textiles—comprised 270 MMT, or around 90% of total volume (Figure 1). Plastic applications are chosen from these sectors and compared to nonplastic alternatives based on their GHG emissions impact.

Sixteen applications are selected across the sectors: seven in packaging, three in building and construction, two in consumer goods, two in automotive, and two in textiles. Each application is representative of its respective sector’s subcategory and the product mixes found in the US 2020 market, which are considered a reasonable proxy for the global average. EoL disposal rates are obtained from the EPA’s Advancing Sustainable Materials Management report and expert interviews.^20^Table S2 shows the alternatives modeled for each application in each sector, including the functional unit used.

We focus on plastic and paper grocery bags, excluding reusable grocery bags due to the wide array of volumes and materials used and a lack of reliable data about reuse, which can have a critical impact on the life cycle of these alternatives. We also exclude compostable and biodegradable alternatives; although these alternatives hold promise for reducing GHG emissions, they currently account for less than 1% of the plastic market (at approximately two million tons annually).^27^

### 2.6 Sensitivity Analyses for Selected Applications

To complement the US 2020 view, sensitivity analyses are undertaken for western Europe and China and in a 2050 decarbonized, circular world for two illustrative applications: soft drink containers and milk containers (further details of scenarios can be found in Section 3.1). The sensitivity analyses explore three variables that are likely to change between now and 2050: the energy mix, the EoL disposition mix, and the vehicle powertrain mix of battery electric vehicles (BEV) versus the internal combustion engine vehicles (ICEV). The energy mix of the base-case and best-case scenarios are derived from the IEA’s Stated Policies Scenario (STEPS) and Sustainable Development Scenario (SDS), respectively.^22^ The EoL disposition and BEV versus ICEV mix for both cases are based on previous studies^25^ and expert interviews.^28^

## 3 Results and Discussion

Our results indicate that in 15 of the 16 applications a plastic product has the lowest greenhouse gas emission impact. This includes 14 applications where a plastic-based product is compared with alternative materials such as metal or glass and two applications where plastics are compared with plastic-enabled mixed materials (i.e., water cups and milk containers). In these latter two applications, the difference is less pronounced, and the GHG profiles for the plastic and plastic-enabled materials are similar.

In the 13 applications where a plastic product has lower emissions than its nonplastic alternatives, the GHG emission impact is between 10% and 90% lower than the next-best alternatives (Table 1). This includes indirect impacts, such as fuel savings in lighter cars, lower energy consumption in houses insulated with polyurethane, and reduced food spoilage when using plastic packaging instead of butcher paper. If we exclude the indirect impacts and only compare direct life-cycle emissions (production, retail transport, and end-of-life disposition), a plastic product has the lowest GHG impact in nine out of 14 applications. Depending on the application, this is generally due to one of two factors: (1) plastics are less energy intensive to produce, for example, polyethylene terephthalate (PET) versus aluminum net of recycling rates, or (2) plastics are more, a plastic product has the lowest GHG impact in nine out of 14 applications, i.e., weight efficient (such as PET versus glass).

**Table 1:**
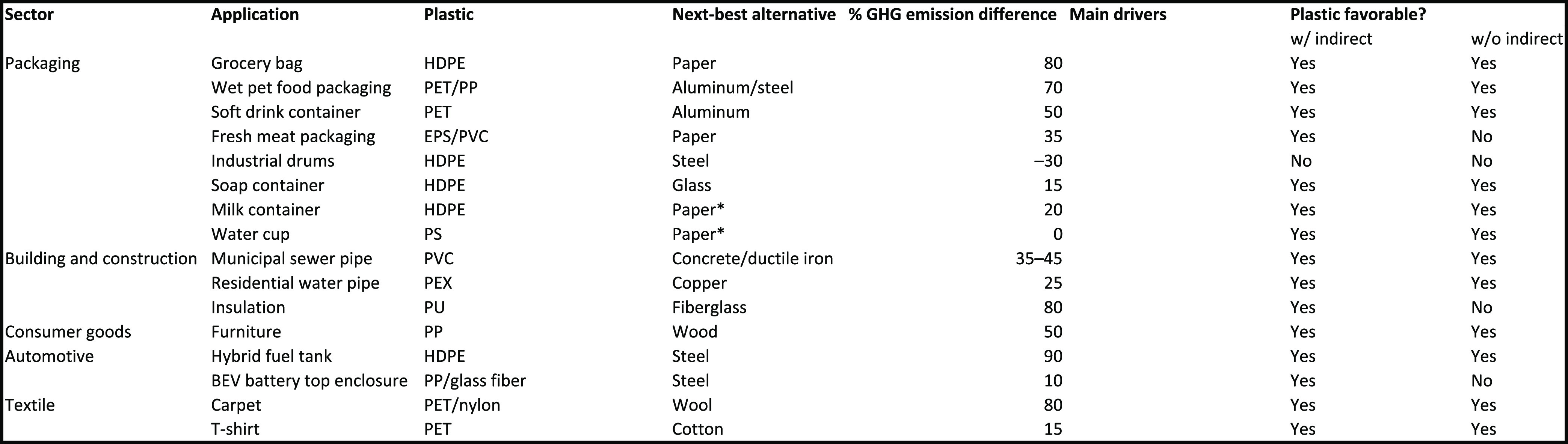
Climate Impact of 16 Plastic and Nonplastic Alternative Applications^a^

a EPS (expanded polystyrene), HDPE (high-density polyethylene), PET (polyethylene terephthalate), PEX (cross-linked polyethylene), PP (polypropylene), PU (polyurethane), and PVC (polyvinyl chloride). * denotes plastic-enabled mixed materials.

Plastics have a lower impact on the upstream processes (production and transport) in 10 of 16 applications. Depending on the application, this is due to one of two factors: plastics being less energy intensive to produce per unit weight of material (e.g., PET vs aluminum) or plastics being lighter and requiring less material weight for the same functional unit, thereby reducing production (and transportation) emissions (e.g., PET vs glass).

Indirect impacts in the background system, relating to energy used for heating, cooling, and transport, can be substantial. For both insulation and hybrid vehicle fuel tanks, the indirect impacts from the use and end-of-life phases far outweigh the direct impact of the plastics in the product. In the former, polyurethane insulates better than glass fiber batt and thus reduces heating fuel consumption, while in the latter, plastic tanks reduce vehicle weight and thus are more fuel efficient.

There are few alternatives to plastics in food packaging across a broad range of applications. This is primarily due to higher levels of food spoilage when using nonplastic alternatives. An evaluation of 20 common food categories reveals that plastic packaging is used in more than 90% of products sold in six categories (breakfast cereal, yogurt, cheese, still bottled water, and fresh and frozen meat). In another eight categories (milk, edible oil, chocolate, nut/seed mix, sweet biscuit, packaged bread, juice, and rice), plastics are present in the packaging of more than 50% of products sold. The remaining six categories (ice cream, carbonated soft drink, pasta, jam and preserve, soup and pickled products) use plastics in less than 50% of the products sold, as plastics have viable alternatives in use.^29^ The role of plastic packaging in keeping food from spoiling translates into a significant but often unquantified GHG benefit relative to alternatives.

In one of the 16 applications, industrial drums, steel remains preferable to plastics due to its durability and recyclability. While a steel drum has higher levels of GHG emissions in production, it lasts twice as long and is typically recycled at EoL. For water cups, the emissions are close to equal. This is because the plastic and nonplastic alternatives weigh about the same, leading to similar emissions for production and transportation activities. Conversely, in the application of grocery bags, paper bags weigh significantly more than HDPE bags, leading to higher GHG emissions for production and transportation. Unsurprisingly, materials that are more durable, lighter, or recycled generate lower GHG emissions.

### 3.1 Packaging Plastics

#### 3.1.1 Soft Drink Containers (PET vs Glass Bottle vs Aluminum Can)

**Figure 2:**
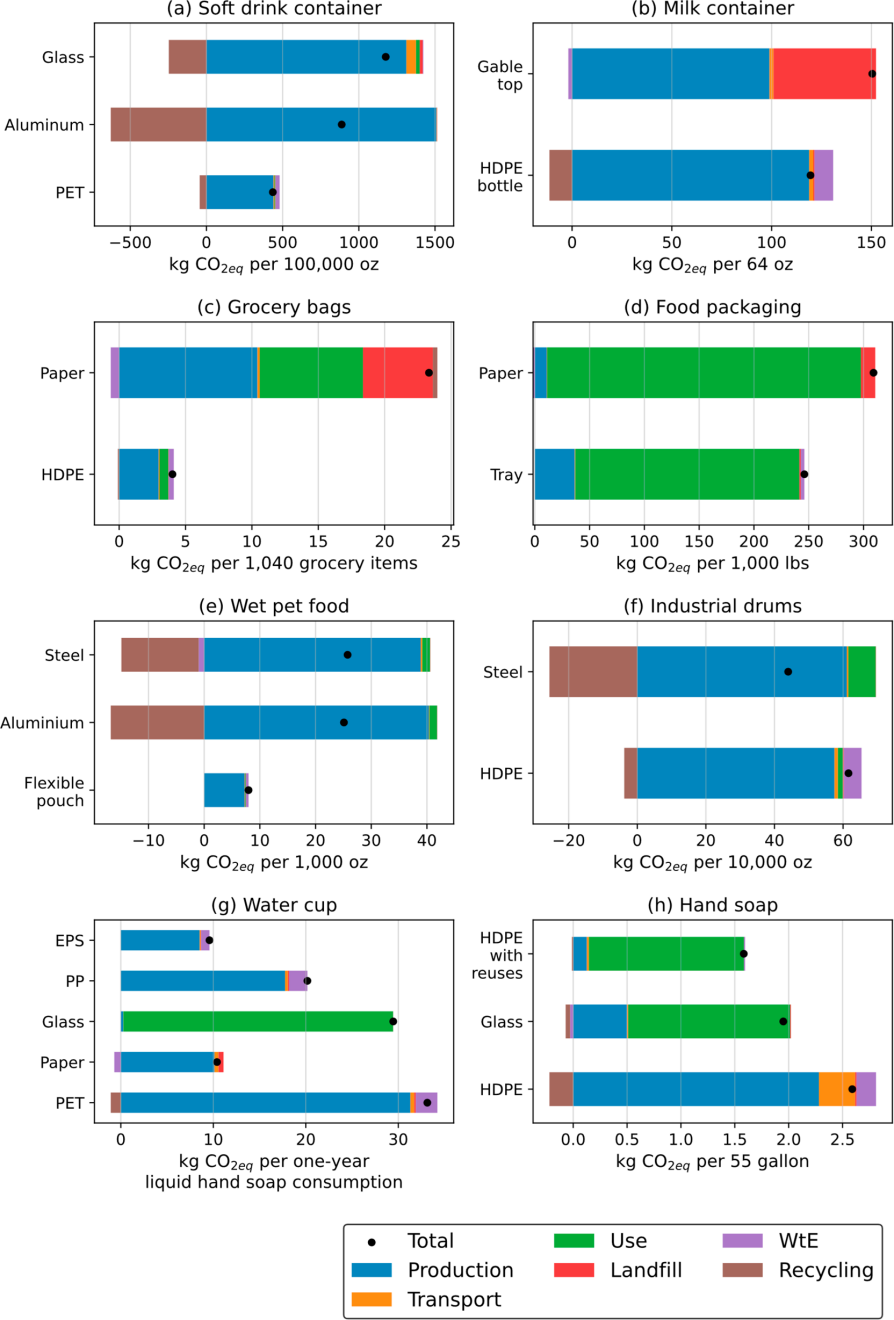
Total life-cycle GHG emissions (kgCO_2eq_ per functional unit) for all packaging plastics. The production stage includes emissions from raw material acquisition and manufacture as well as adjustments made to the functional unit for additional production of containers required to compensate for spoilage and breakage.

Currently, most soft drinks are packaged in poly(ethylene terephthalate) (PET) bottles, aluminum cans, or glass bottles. We base our analysis on 20-ounce PET bottles, 12-ounce aluminum cans, and 12-ounce (355 mL) glass bottles, which have 17%, 60%, and 0.3% of the carbonated soft drink market share in the United States, respectively (Figure 2a). These specific sizes are selected because they represent the most common beverage container sizes for their respective material substrates. Comparing a 20-ounce (591 mL) PET bottle with a 12-ounce aluminum could favor the PET bottle because the material-to-volume ratio is significantly higher for smaller containers, as it would require more plastics to distribute 100,000 fluid ounces (2.84 L) of soda in 12-ounce PET bottles than in 20-ounce PET bottles, which would increase the GHG emission profile. However, these sizes represent what consumers typically choose to purchase.

PET bottles have the lowest emissions impact because of their low weight and low energy intensity during production. In comparison, aluminum cans release twice the emissions of PET bottles, and glass bottles release three times the emissions. PET has the lowest recycling rate (Table S3) among the three alternative containers and the highest emissions when incinerated at end of life (WtE). However, in this case, the production stage dominates the overall emissions, and here, PET has a much lower impact than glass and aluminum (Figure S3). These results agree with the published literature.^30,31^

The average shelf life for PET bottles is approximately 13 weeks compared with 52 weeks for aluminum cans and glass bottles. PET bottles also have slightly higher spoilage rates (loss of carbonation) than aluminum and glass. However, glass bottles break more easily than PET and aluminum. In both cases, additional GHG emissions are incurred from soft drink and bottle production to compensate for the incremental spoilage and breakage of PET and glass bottles.

#### 3.1.2 Milk Containers (HDPE Milk Bottle vs Gable-Top Carton)

In the United States, refrigerated dairy milk is primarily sold in HDPE bottles and gable-top cartons, which are composed of 80% paper and 20% low-density polyethylene (LDPE) (Figure 2b). The 64-ounce (1.8l) HDPE milk bottles have a market share of approximately 75% in the United States, while gable-top cartons account for around 25%. This case is a comparison between plastic and plastic-enabled mixed materials, unlike the majority of applications selected in this study. Without the layer of LDPE, the paper would not be able to contain the milk; LDPE is extremely important, despite constituting only 20% of the carton weight.

Our analysis shows HDPE bottles have lower climate change impact than gable-top cartons in the United States (Figure S6). While gable-top cartons emit around one-third fewer GHGs than HDPE bottles during the production phase, EoL disposal emissions narrow the difference. Gable-top cartons contain paper that generates methane when landfilled, and the paper content is not recycled at scale in the United States. HDPE bottles have significant recycling rates (around 30%; see Table S13) which means that despite having higher emissions when incinerated, they generate lower GHG emissions overall at EoL. In a direct comparison, this has a different result as in the WRAP report.^32^ This is primarily due to the different designs (our study has a lightweight design in 2020 versus the WRAP study done in 2007–2009) and model assumptions. The weight and material composition of the milk packaging systems in our study are directly measured for HDPE bottles (47 g) (75% market share) and gable-top cartons (76 g) (25% market share) with the United States market in 2020 as shown in Table S14.

#### 3.1.3 Grocery Bags (HDPE vs Paper Bag)

A typical paper grocery bag has approximately 25% more carrying capacity but is around six times heavier than a typical HDPE bag (55g vs 8g).^33^ Paper grocery bags have three times the production emissions of HDPE bags due to the higher raw material usage and transportation emissions.^34^ The GHG emissions of paper bags versus HDPE widen further to five times when accounting for EoL disposition and impact in use (such as “double bagging”). In the United States, where landfill is more common than WtE (80% vs 20%), HDPE bags have a more favorable EoL climate impact than paper when landfilled. This is because landfilling paper results in significant methane emissions from anaerobic decomposition, whereas plastics remain almost completely inert in the ground.

Marine litter is excluded from the EoL scenario for grocery bags, as the US has a mature waste management system with minimal leakage to the environment. However, in countries with undeveloped waste management systems, significant leakage to water bodies can occur for consumer plastics, such as grocery bags. On average, 20% of plastic bags and 50% of paper bags are double bagged to compensate for breakage and leakage, increasing the emissions impact of paper bags (Figure 2c, Table S26, and Figure S9).

#### 3.1.4 Food Packaging (EPS Foam Tray + PVC Film vs Butcher Paper)

In the United States, the two most common fresh meat packaging options are expanded polystyrene (EPS) foam trays with poly(vinyl chloride) (PVC) film and butcher paper. We chose pork as a representative of meat products. EPS foam trays are closed cell with absorbent pads. Although EPS foam trays with PVC film have higher production emissions than butcher paper, the lower rates of spoilage for pork in EPS or PVC compared with butcher paper (approximately 5% vs 7%–10%) more than make up the difference. This results in around 35% lower overall climate impact for EPS or PVC than for butcher paper. Furthermore, the high landfill rate of fresh meat packaging in the United States favors plastics over paper because of methane emissions from the anaerobic decomposition of paper (Figure 2d and Figure S10).

#### 3.1.5 Wet Pet Food Containers (Multilayer Pouch vs Aluminum vs Steel Can)

The wet pet food market is dominated by plastic and metal packaging. Flexible multilayer pouches made from polypropylene (PP) (75%), aluminum foil (20%), and PET (5%), constitute approximately 30% of the US market share. Metal cans made from aluminum and steel make up 45% and 15% of the US market share, respectively. Compared to plastic pouches that are not recyclable because of the mixed materials used to produce them, aluminum and steel cans have recycling rates of around 50% and 70%, respectively. Despite higher recycling rates, metal cans tend to be heavier, with aluminum cans weighing 1.5 times and steel cans five times the plastic multilayer pouches, resulting in higher production emissions. These high production emissions counterbalance the avoided burdens from recycling metal cans, leading to overall GHG emissions that are about three times higher than those of plastic multilayer pouches (Figure 2e and Figure S11).

#### 3.1.6 Industrial Drums (HDPE vs Steel Drum)

The relative climate change impact of HDPE versus steel drums stems from differences in production emissions, durability, and recycling rates. The production emissions for steel drums are higher than those for HDPE drums. However, over a lifetime of 10 years, the higher durability of steel drums (10-year lifespan) compared with HDPE drums (five-year lifespan) more than negates the difference in per-drum production emissions. Furthermore, the higher recycling rate of steel drums and HDPE drums (80% and 20%, respectively) and the greater avoided emissions from using recycled rather than virgin steel ultimately tip the balance in favor of steel drums, even after accounting for higher levels of maintenance required to fix dents in steel drums. Overall, using a single steel drum instead of two HDPE drums over 10 years results in approximately 25% lower climate impact (Figure 2f and Figure S12).

#### 3.1.7 Water Cups (EPS vs PP vs PET vs Paper vs Reusable Glass Cup)

We assess the climate change impact of three types of plastic cups (EPS, PET, and PP) compared with paper and reusable glass cups. The EPS cups have the lowest GHG emissions because they have the lowest weight and production emissions. Paper cups have similar GHG emissions to EPS cups because of their low production emissions and because the WtE CO_2_ emissions from paper combustion can be excluded owing to neutral biogenic carbon. However, paper cups contain approximately 5% LDPE by weight and are considered a plastic-enabled mixed material. As with gable-top milk cartons, the LDPE lining enables paper cups to hold liquids. Emissions from reusable glass cups are highly sensitive to the washing process, especially the choice of water temperature (hot versus ambient). We estimate that one glass cup can be reused up to 500 times and can be washed with hot water in a commercial dishwasher in batches of 50.^35^ Using hot water results in five times the climate change impact of using ambient water because of the use of industrial gas boilers, which have a relatively high climate change impact. Thus, if reusable glass cups are washed with ambient water, they will have a lower GHG impact than both EPS and paper cups (Figure 2g and Figure S13).

#### 3.1.8 Hand Soap Bottles (HDPE vs Glass Hand Soap Bottle)

Our analysis of hand soap bottles clearly illustrates the climate change benefits of reuse. Refilling a glass bottle 15 to 20 times with the contents of flexible PP pouches results in an approximately 25% lower climate change impact than using 15 to 20 HDPE hand soap bottles. These figures are driven by lower production emissions of flexible PP refilling pouches compared to rigid HDPE bottles, even with the consideration of soap wastage when refilling. However, reusing HDPE bottles has the lowest GHG emissions, with 15% lower emissions than reusing glass bottles (Figure 2h and Figure S14).

### 3.2 Building and Construction Plastics

#### 3.2.1 Municipal Sewer Pipes (PVC vs Concrete Vs Ductile Iron)

**Figure 3:**
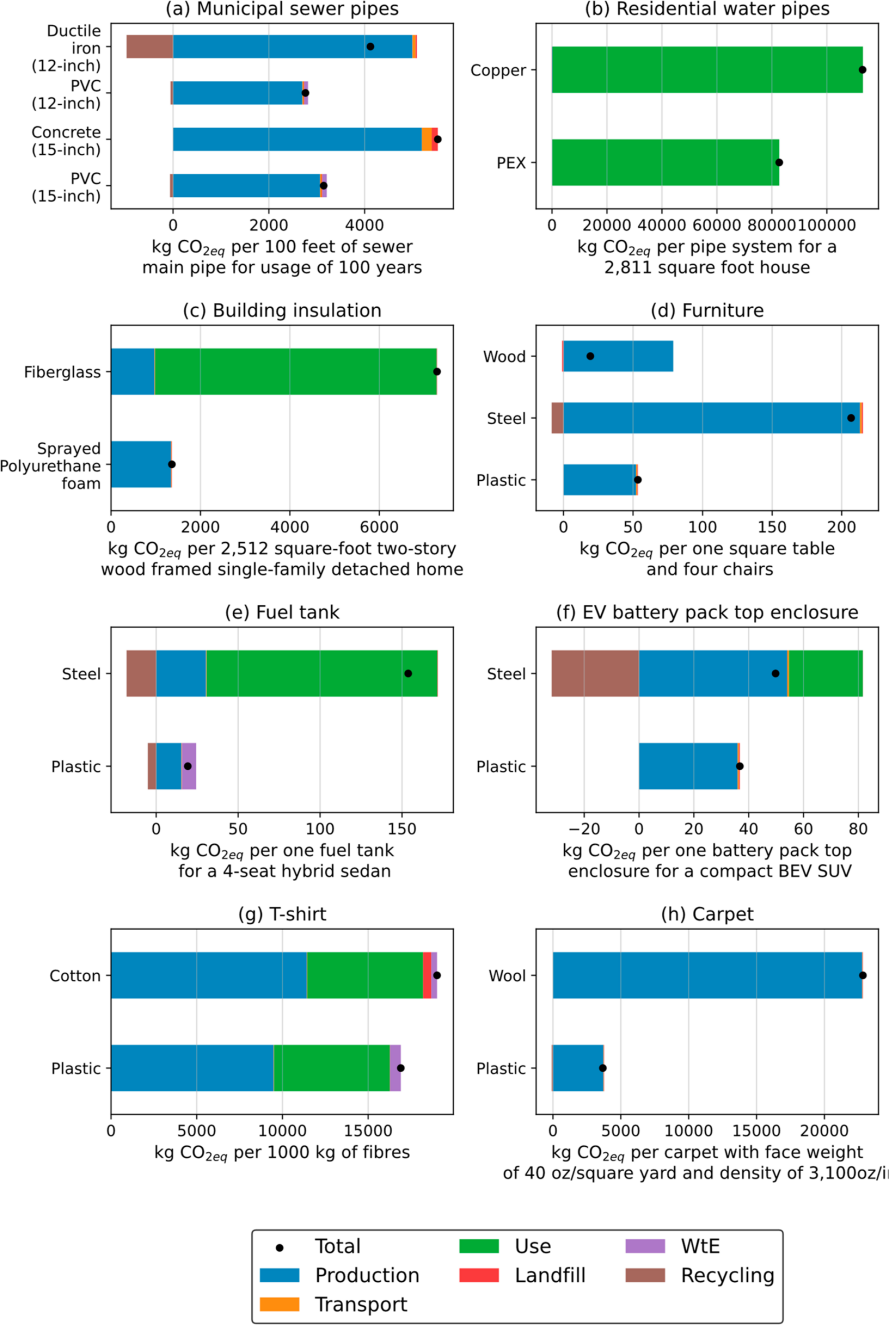
Total life-cycle GHG emissions (kgCO_2eq_ per functional unit for all building and construction plastics (a–c), all consumer goods plastics, represented by a furniture set (d), all automotive plastics (e, f), and all textile plastics (g, h). In (a), 15 in. is for sewer gravity main pipe, and 12 in. is for sewer force main pipe (see SI, section 10 for details).

There are two main types of sewer pipes: gravity pipes (with approximately 90% of the market share) and force main or pressure pipes (around 10%). PVC and reinforced concrete are the most common materials used in gravity pipes, while PVC and ductile iron are most prevalent in force main pipes. To ensure a fair comparison, we base our assessment on the pipe specifications that are the most comparable. For the 15 in. (375 mm) sewer gravity main pipe, we compare PVC with reinforced concrete. For the 12 in. (300 mm) sewer force main pipe, we compare PVC versus ductile iron. All four pipes are assumed to have a service life of 100 years.^36^ In both sewer pipe applications, PVC has the lowest climate change impact (approximately 45% lower than reinforced concrete and 35% lower than ductile iron) primarily because of its ability to achieve the same function with lighter weight. Concrete and ductile pipes also require more GHG-intensive transport and installation processes. It is noted that ductile iron pipes have comparatively higher recycling rates (around 30%) than PVC pipes (around 10%), but most pipes are not recovered from the ground at end of life. We have not been able to quantify pumping efficiency for force main pipes, but it would favor PVC, which is already the material with the lowest climate change impact (Figure 3a and Figure S15).

#### 3.2.2 Residential Water Pipes (PEX vs Copper)

Copper type L and PEX pipes are two common examples of residential water pipes. The most important factor when comparing the climate change impact of copper with PEX pipes is that copper has a higher thermal conductivity than plastics.^37^ We estimate that the climate change impact from incremental heat loss is around 35% higher in copper pipes than PEX pipes in a 2811 ft^2^ (261 m^2^) home where most water use for a family of four is concentrated in the mornings and evenings. The production emissions of copper pipes are also around 2.5 times those of PEX pipes because of their heavier weight and more energy-intensive production process. However, the difference in production emissions is minimized by the difference in the incremental heat loss. Although copper is highly recyclable, its potential is not fully captured, because small-scale residential demolition contractors often fail to remove and sort copper pipes for recycling. Hence, the US recycling rate is estimated to be only 30%. By contrast, PEX pipes are rarely recycled. Overall, PEX pipes have around 3% lower climate change impact than copper pipes (Figure 3b and Figure S16).

#### 3.2.3 Building Insulation (PU vs Fiberglass)

Our assessment of the climate impact of building insulation considers residential in-wall insulation for new buildings. The market share in the United States varies by region, but on average, fiberglass batt represents 60%–70% of the market, with spray polyurethane foam (SPF) making up the second-largest share (20%–30%). The remaining insulation types include foam boards (expanded polystyrene or polyisocyanurate), which are mostly used as continuous wall insulation, mineral wool, and blown cellulose, which are more commonly used for renovation rather than new buildings.

We base our analysis on a recent LCA with energy-modeling analysis published by the Spray Polyurethane Foam Alliance^38^ that analyzed external wall insulation requirements for a 2512 ft^2^ (233m^2^) two-storey wood-frame house in Richmond, Virginia. This region was selected because it represents a median US climate zone (IECC climate zones in the US mainland range from 1 to 7; Richmond is in zone 4). To reach the building code standard of R = 20 for external walls in Richmond, 360 kg of fiberglass batts and 330 kg of open-cell SPF are required, which are adopted in this study as reference flows. Both alternative materials are assumed to be landfilled at their end of life.

The main contribution to climate change is the use phase, which is driven by the permeability of fiberglass to air, in contrast to SPF, which is impermeable. The permeability of fiberglass also allows for greater heat transfer, which requires more heating and cooling throughout an insulation lifetime of 75 years. The overall result is that SPF has a higher initial impact at production, but its incremental GHG savings from the use phase lead to approximately 80% lower impact across the insulation’s lifetime when compared with fiberglass batt (Figure 3c and Figure S17).

### 3.3 Consumer Goods Plastics

#### 3.3.1 Furniture Set (PP vs Steel vs Wood)

We model furniture as a representative example of consumer durable goods and defined the functional unit as a set of one square table and four chairs with a lifespan of 10 years. For this analysis, we assess the climate change impact of three common furniture materials: PP, wood, and steel (Figure 3d). The PP furniture set has the lowest climate change impact, primarily because it requires less material to provide similar performance and functionality (around 20 kg for PP vs 40 kg for both wood and steel), which reduces the emissions associated with raw material acquisition, manufacturing, and transport.

### 3.4 Automotive Plastics

#### 3.4.1 Automotive Fuel Tanks (HDPE vs Steel Fuel Tank)

For vehicle automotive applications, most GHG impact stems from impacts of the use phase on a mass basis. We define the functional unit as a fuel tank for a midsized hybrid sedan in the United States with a lifetime mileage of 200,000 miles and compare HDPE and steel fuel tanks. The lighter weight of HDPE fuel tanks compared to steel results in approximately 14 times fewer GHG emissions overall. HDPE and steel have comparable GHG emissions at production and EoL, so the overall difference is primarily due to the greater fuel efficiency of the lighter HDPE tanks. The recycling rate of automotive steel, including fuel tanks, is about 95%, while the rates for HDPE fuel tanks are comparatively lower (at about 65%) (Figure 2e and Figure S19).

#### 3.4.2 Automotive Electric-Vehicle Battery Pack Top Enclosures (PP vs Steel Battery Enclosure)

We select battery pack top enclosures as a representative application in BEVs. The two most common material types are steel and a composite material composed of PP and fiberglass reinforced PP.

PP/fiberglass battery enclosures emit around 10% fewer emissions than steel enclosures over their lifetime mileage of 200,000 miles. EVs have not yet reached EoL at scale, so our recycling rates are estimated based on expert interviews. Composite PP/fiberglass enclosures emit less at the production stage, but their mixed-material nature presents a challenge for recycling. Plastic battery housing enclosures are also lightweight, providing an opportunity to reduce the battery size and avoid emissions associated with battery production. Reduction in battery size is possible from BEV light-weighting if BEVs can maintain a minimum acceptable range of 250–300 miles, achieve light-weighting at a reasonable cost, and achieve a material weight reduction of at least 20–30 kg, as the additional BEV weight can push a vehicle into the next weight class. Unlike plastics, steel enclosures are expected to have a high recycling rate of around 95% by participating in existing steel recycling flows. Still, they require more electricity consumption over their service life because of their heavier weight (Figure 3f and Figure S20).

### 3.5 Textile Plastics

#### 3.5.1 T-Shirts (PET vs Cotton)

Apparel contributes around 50% of the textile sector’s total 11 MMT plastic volume. We select t-shirts as a representative application, comparing the climate change impact of PET shirts with that of cotton t-shirts. Overall, PET t-shirts have a lower climate change impact than cotton t-shirts, primarily because of lower production emissions. Cotton emits a considerable volume of GHG emissions across the various stages of crop cultivation, such as in the use of agrochemicals and irrigation. Additionally, it is worth noting that t-shirts are not generally recycled,^39^ so EoL disposal is split almost equally between WtE and landfill (Figure 3g and Figure S21).

#### 3.5.2 Carpets (Synthetic vs Wool)

Carpet is another major textile category, corresponding to approximately 1 MMT (or 10%) of the total textile plastic volume. A majority (around 85%) of the carpet market is dominated by synthetic carpet (PET/nylon).^40^ The only nonplastic alternative is wool, which constitutes only 3%–5% of the US market share and is primarily used in high-end carpets. Synthetic carpet emits five times fewer GHGs than wool carpet due to significantly lower production emissions. Only around 5% of synthetic carpet is recycled in the United States, mainly in California. Further increases in carpet recycling rates would widen the difference in the climate change impact of PET/nylon versus wool since the latter cannot be recycled (Figure 3h and Figure S2).

### 3.6 Sensitivity Analysis: Opportunities to Reduce GHG Impact Across Materials

We perform a sensitivity analysis that extends our assessment to western Europe and China and project a scenario of a decarbonized, circular world in 2050. We model three main drivers: the energy mix, the EoL treatment mix, and the BEV versus ICEV mix for the transportation of plastic/plastic alternatives. The energy mix affects process energy, while the BEV versus ICEV mix impacts transport energy. Process nonenergy is assumed to be constant. This streamlined approach offers a high-level perspective of regional nuances and a scenario for 2050 to help identify the key abatement levers for each product analyzed. The sensitivity analysis focuses on soft drink and milk containers.

#### 3.6.1 Soft Drink Containers

**Figure 4:**
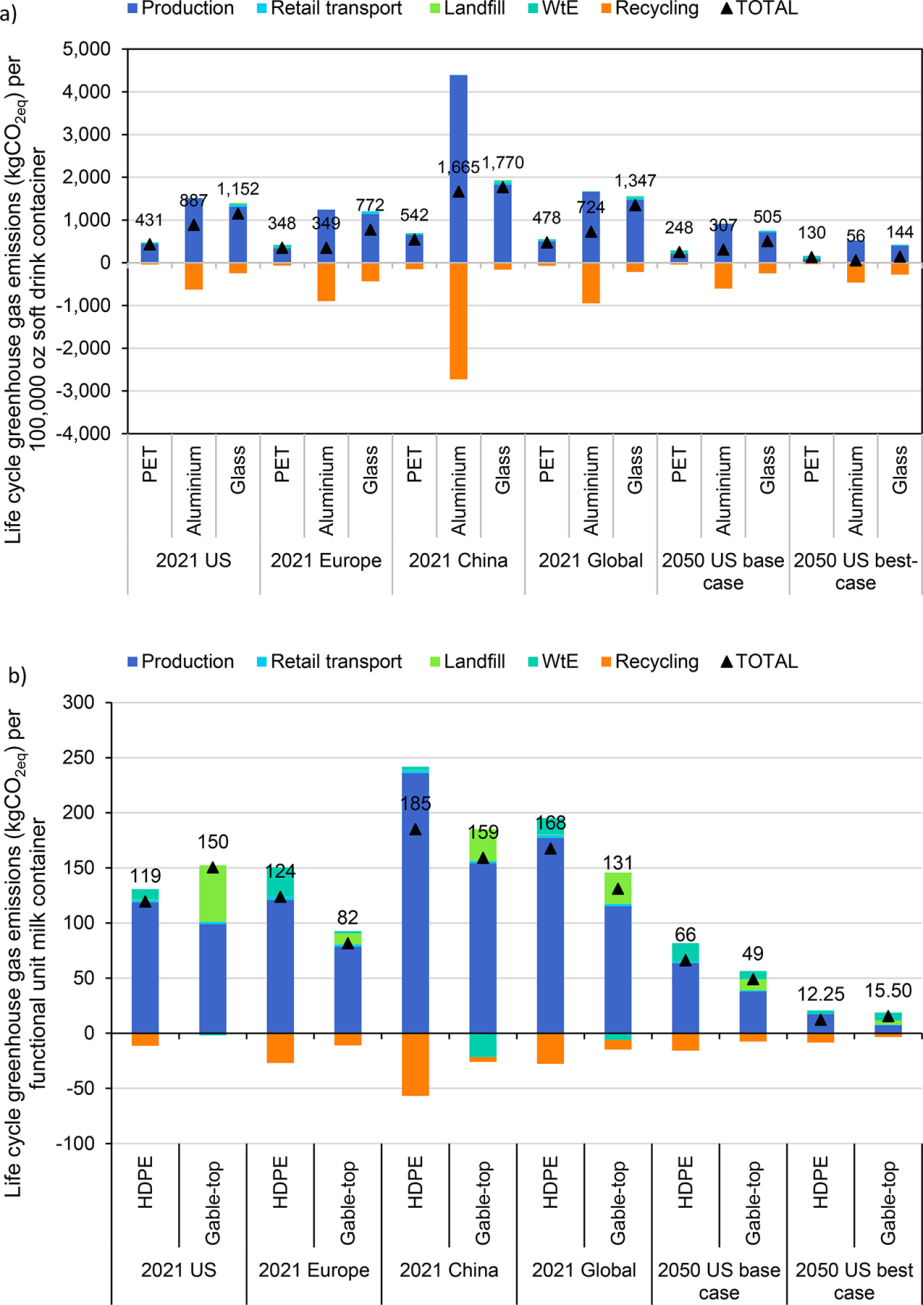
(a) Soft drink container regional 2020 and US 2050 scenarios: kgCO_2eq_ per 100,000 oz of soft drink. The aluminum can is competitive with PET bottles in western Europe but has a higher climate change impact in China. Aluminum and glass disproportionally benefit from decarbonizing the electric grid. (b) Milk container regional 2020 and US 2050 scenarios: Gable-top carton has a lower GHG impact than HDPE bottle in western Europe and China due to higher recycling/WtE vs landfill mix. In a decarbonized world in 2050, both HDPE bottles and gable-top cartons have low GHG emissions, with HDPE having a slight advantage due to a higher recycling rate.

The relative performance of PET, aluminum, and glass varies by region. Although PET bottles have the lowest climate change impact in the United States, aluminum cans have the lowest climate change impact in western Europe, while glass bottles still have the highest emissions (Figure 4a). This is because western Europe has a cleaner energy mix and higher recycling rates for aluminum cans (Figure S4). Unlike PET and glass, aluminum production uses a high share of hydropower in the United States and western Europe, and mostly coal in China (Figure S4).

Western Europe imports around 50% of its aluminum ingots from Iceland, Mozambique, Norway, and the United Arab Emirates, among others, suggesting that the true climate change impact may be higher than that calculated. By contrast, China has the highest overall impact for all materials because of its coal-reliant energy mix (Figure S4). China’s higher recycling rates of PET bottles and aluminum cans do not sufficiently compensate for this coal-heavy energy mix (Figure 4a and Figure S4).

In our 2050 base case, a cleaner energy mix, higher recycling rates, and greater commercial BEV penetration reduce the overall GHG emission impact of all three materials. The energy-intensive nature of production means both aluminum and glass experience particularly significant advantages when the grid is decarbonized (Figure S5). They also derive some benefits, although to a lesser degree, from reducing the need for new production through recycling. Moreover, as PET emissions in 2050 will be primarily driven by emissions from WtE, and since its production process is less energy-intensive, PET emissions will decrease relatively slowly compared with aluminum and glass, leading to a narrowing of the difference in climate change impact between PET and aluminum or glass. In fact, under the 2050 best-case scenario (a 1.5 °C pathway), aluminum cans have a lower climate change impact than PET bottles.

#### 3.6.2 Milk Containers

In all regions investigated, HDPE bottles are associated with a lower climate change impact relative to gable-top cartons because of their higher rates of recycling or WtE compared to landfills (Figure 4b). This is in line with findings from published reports in Europe,^41^ Australia, and New Zealand.^42^ The energy mix has similar impacts on both HDPE bottles and gable-top cartons, with overall emissions in western Europe being lower than those in the United States. Emissions are consistently high in China. However, it is worth noting that HDPE milk bottles tend to be much less common in the United States.

In 2050, a decarbonized US energy mix will significantly lower the GHG impact of both products. In the base case, for which there is an overall increase in WtE rates and a sizable increase in recycling rates for gable-top cartons, gable-top cartons outperform HDPE bottles. In the best-case scenario (100% renewable or nuclear energy and high recycling), both products generate low GHG emissions, but HDPE has a lower GHG impact because of its higher recycling rates (Figure 4b and Figures S7–S8).

### 3.7 Discussion

Plastics are ubiquitous across the global economy and the subject of frequent debate, from their contribution to marine pollution to recycling. This is because plastics do not break down in the environment, resulting in accumulation in waterways, agricultural soils, rivers, and the ocean over decades. More recently, that concern has expanded to the impact of plastics on ecosystems, food and water supplies, and human health, amidst emerging evidence that plastics are accumulating not only in our environment but also in our bodies. Calls to use less plastics have garnered popular appeal in the drive to combat climate change and ocean pollution. On the other hand, global demand for plastics is expected to triple between 2019 and 2060, from 460 to 1321 Mt.^43^ This anticipated growth of plastic production is of real concern, but we need to recognize that production is growing in response to the increasing global demand for enhancing fuel efficiencies from lightweighting and decreasing food spoilage in product packaging. All of these will play an important role in reducing GHG emissions and helping people live more sustainably around the world, which is often overlooked. We must be mindful not to fix a problem by removing one of the solutions.

This paper examines the climate change impact of plastics versus their alternatives over the full life cycle (cradle to grave). Our analysis is based on the United States in 2020 and includes a sensitivity analysis to illustrate the impact in other regions and show how results change as we move toward a decarbonized world in 2050. We look closely at examples from the five sectors with the highest plastic consumption—packaging, building and construction, automotive, textiles, and consumer durables—which represent around 90% of global plastic volume. This paper shows that in almost all cases switching out plastics for another material increases emissions by between 10% and 90%. We also select representative applications for which current large-scale, viable alternatives to plastics exist, thus avoiding unproven and infant solutions. Indirect value-chain impacts can be substantial. In both insulation and hybrid-vehicle fuel tanks, the indirect impact far outweighs the direct impact. In the former, polyurethane insulates better than glass fiber batt and thus reduces heating fuel consumption, while in the latter, plastic tanks reduce vehicle weights and thus improve fuel efficiency. These indirect impacts offset plastics’ generation of more GHG emissions than the nonplastic alternative in the production and disposal phases. This is not universal, however. The indirect impact in many applications is nonmaterial. For example, the indirect impact of decreased breakage in plastic bottles versus that in aluminum cans or glass bottles is insignificant.

Reducing the environmental impacts of plastics such as grocery bags is not just about choosing, banning, recommending, or prescribing specific materials or bags but also about changing consumer behavior to increase the reuse rate and avoid littering. Across most applications, simply switching from plastics to currently available nonplastic alternatives is not a viable solution for reducing GHG emissions. Therefore, care should be taken when formulating policies or interventions to reduce plastic demand that they result in the removal of the plastics from use rather than a switch to an alternative material. For example, removing the plastic wrappers from fruit and making use of the natural fruit skin for protection makes sense, but switching from plastic drinking straws to paper alternatives does not. Material choices should be grounded in scientific facts rather than influenced by popular beliefs.

We conclude that applying material substitution strategies to plastics never really makes sense. This is because plastics’ inherent properties—strong, lightweight, easy to shape, customizable, and comparatively low-GHG emissions—make it the preferred material for minimizing emissions across most products. If material substitution is not the answer, then what should we do to reduce emissions from plastics? Our 2050 base- and best-case scenarios suggest that policy actions should focus on promptly delivering the best-case scenario, including decarbonization of energy sources and material efficiency strategies, rather than continuing the current approach, which drives a shift from plastics to other materials. Greater leverage for reducing emissions is provided by alternative strategies that reduce plastic use by extending the lifetime of products. Doubling the lifetime of a plastic product, by, for example, using the product a second time, can give up halving emissions. This strategy works regardless of the material used. Ensuring plastics can be reused/recycled and are reused/recycled is another effective strategy. Every time a drinking cup is reused, the emissions are drastically reduced, even when washing the cup, which can be balanced against the reduction in waste management and transport for single-use products. The question becomes which material allows us to reuse the cup many times (i.e., plastics, ceramic, or metal) and how can we ensure that the cup is reused (i.e., price, avoiding breakage). Policies should focus on reducing demand for single-use products, regardless of the material and avoid singling out plastics. Robust regulations and policies play a crucial role in supporting such initiatives, which are essential for society to attain a truly circular and sustainable state.

This study is offered as a first step toward what must be a larger, urgent dialogue about the role of the plastic life cycle in the GHG emission impact. Future modeling can be expanded to include reusable bioplastics and compostable and biodegradable alternatives which are currently excluded in this study due to small market values and a lack of reliable data about reuse. It is crucial not to overlook the significant and unacceptable impact of plastics on marine ecosystems with potential impacts on human and ecological health that remain insufficiently comprehended. This complexity adds a layer of intricacy to the decision-making process when weighing the trade-offs between GHG emissions and marine pollution as well as considering the broader environmental and health implications in material selection. Subsequent endeavors should assess these trade-offs using additional environmental impact metrics/planetary boundary impacts.^44,45^ This includes factors such as non-GHG air emissions, plastic waste in waterways, toxicity, and microplastics from manufacturing, use phases, and recycling, enabling the development of integrated strategies for a sustainable plastic sector. Actions should be targeted to reduce these impacts, for example, by improving waste collection, especially in developing countries, removing toxic chemicals from plastic formulations, reducing the use of forever chemicals (i.e., perfluoroalkyl and polyfluoroalkyl substances), and bolstering recycling and recovery programs. However, any action taken or policy employed to reduce the impacts of plastics needs to be examined carefully to make sure that GHG emissions are not unintentionally increased through a shift to more emission-intensive alternative materials. Extending the lifetime of materials and products, through better design, reuse, and recycling, is a win–win strategy; it is effective at mitigating both carbon emissions and other environmental impacts. Switching to alternative materials is not.

## Supporting Information Available

Additional details of methods and assumptions of life-cycle assessment for each type of plastics including functional unit, life-cycle inventory over the life-cycle stages, greenhouse gas emissions results, sensitivity analysis method and results (PDF)

## Abbreviations

BEV: Battery electric vehicle
EoL: End of life
EPA: US Environmental Protection Agency
EPS: Expanded polystyrene
GHG: Greenhouse gas emissions
HDPE: High-density polyethylene
HDPE: High-density polyethylene
ICEV: Internal combustion engine vehicle
IEA: International Energy Agency
LCA: Life-cycle assessment
LDPE: Low-density polyethylene
PET: Polyethylene terephthalate
PEX: Cross-linked polyethylene
PP: Polypropylene
PU: Polyurethane
PVC: Polyvinyl chloride
SDS: Sustainable Development Scenario
STEPS: Stated Policies Scenario
WtE: Waste to energy

## References

[ref1] Breaking the Plastic Wave: A Comprehensive Assessment of Pathways towards Stopping Ocean Plastic Pollution, 2020. SYSTEMIQ and The Pew Charitable Trust. https://www.systemiq.earth/wp-content/uploads/2020/07/BreakingThePlasticWave_MainReport.pdf.

[ref2] Carbon Footprint Reference Values. Energy Efficiency and Greenhouse Gas Emissions in European Mineral Fertilizer Production and Use, 2011. Fertilizers Europe. https://www.fertilizerseurope.com/wp-content/uploads/2020/01/The-carbon-footprint-of-fertilizer-production_Regional-reference-values.pdf.

[ref3] Persson L.; Carney Almroth B. M.; Collins C. D.; Cornell S.; de Wit C. A.; Diamond M. L.; Fantke P.; Hassellöv M.; MacLeod M.; Ryberg M. W.; Søgaard Jørgensen P.; Villarrubia-Gómez P.; Wang Z.; Hauschild M. Z. Outside the Safe Operating Space of the Planetary Boundary for Novel Entities. Environ. Sci. Technol. 2022, 56 (3), 1510–1521. 10.1021/acs.est.1c04158. 35038861 PMC8811958

[ref4] Climate Change 2022: Mitigation of Climate Change (Working Group III Contribution to the Sixth Assessment Report of the Intergovernmental Panel on Climate Change), 2022. Intergovernmental Panel on Climate Change (IPCC). https://www.ipcc.ch/report/ar6/wg3/ (accessed 2022–04–21).

[ref5] Global Chemicals Outlook. UN Environment Programme. http://www.unep.org/explore-topics/chemicals-waste/what-we-do/policy-and-governance/global-chemicals-outlook (accessed 2022–04–20).

[ref6] Geyer R.; Jambeck J. R.; Law K. L. Production, Use, and Fate of All Plastics Ever Made. Sci. Adv. 2017, 3 (7), e1700782 10.1126/sciadv.1700782. 28776036 PMC5517107

[ref7] Historic day in the campaign to beat plastic pollution: Nations commit to develop a legally binding agreement. UN Environment Programme. http://www.unep.org/news-and-stories/press-release/historic-day-campaign-beat-plastic-pollution-nations-commit-develop (accessed 2023–03–13).

[ref8] Stegmann P.; Daioglou V.; Londo M.; van Vuuren D. P.; Junginger M. Plastic Futures and Their CO2 Emissions. Nature 2022, 612 (7939), 272–276. 10.1038/s41586-022-05422-5. 36477132

[ref9] Global Warming of 1.5 °C: An IPCC Special Report on the Impacts of Global Warming of 1.5 °C above Pre-Industrial Levels and Related Global Greenhouse Gas Emission Pathways, 2018. IPCC. https://www.ipcc.ch/sr15/.

[ref10] Far-reaching ban on single-use plastics in England. GOV.UK. https://www.gov.uk/government/news/far-reaching-ban-on-single-use-plastics-in-england (accessed 2023–03–13).

[ref11] Lau W. W. Y.; Shiran Y.; Bailey R. M.; Cook E.; Stuchtey M. R.; Koskella J.; Velis C. A.; Godfrey L.; Boucher J.; Murphy M. B.; Thompson R. C.; Jankowska E.; Castillo Castillo A.; Pilditch T. D.; Dixon B.; Koerselman L.; Kosior E.; Favoino E.; Gutberlet J.; Baulch S.; Atreya M. E.; Fischer D.; He K. K.; Petit M. M.; Sumaila U. R.; Neil E.; Bernhofen M. V.; Lawrence K.; Palardy J. E. Evaluating Scenarios toward Zero Plastic Pollution. Science 2020, 369 (6510), 1455–1461. 10.1126/science.aba9475. 32703909

[ref12] Communication from the Commission to the European Parliament, the Council, the European Economic and Social Committee and the Committee of the Regions a European - Strategy for Plastics in a Circular Economy, 2018. European Commission. https://eur-lex.europa.eu/resource.html?uri=cellar:2df5d1d2-fac7-11e7-b8f5-01aa75ed71a1.0001.02/DOC_1&format=PDF.

[ref13] European Platform on Life Cycle Assessment- Plastics LCA. European Commission. https://eplca.jrc.ec.europa.eu/plasticLCA.html (accessed 2022–10–18).

[ref14] ISO 14040 Environmental Management - Life Cycle Assessment - Principles and Framework; International Organization for Standardization (ISO): Geneve, 2006.

[ref15] ISO 14044 Environmental management - Life cycle assessment - Requirements and guidelines; International Organization for Standardization (ISO): Geneve, 2006.

[ref16] IEA World Energy Outlook; International Energy Agency: Paris, 2021.

[ref17] Waste Reduction Model (WARM), 2022. US EPA. https://www.epa.gov/warm (accessed 2023–12–22).

[ref18] Life Cycle Inventory Database, 2022. Ecoinvent. https://ecoinvent.org/.

[ref19] Transportation—Commodity Flow Survey. U.S. Census Bureau. https://www.census.gov/library/publications/2015/econ/ec12tcf-us.html (accessed 2023–12–22).

[ref20] Advancing Sustainable Materials Management: Facts and Figures Report, 2022. US EPA. https://www.epa.gov/facts-and-figures-about-materials-waste-and-recycling/advancing-sustainable-materials-management (accessed 2023–12–22).

[ref21] New Plastics Economy: Rethinking the Future of Plastics, 2016. McKinsey & Company. https://www.mckinsey.com/capabilities/sustainability/our-insights/the-new-plastics-economy-rethinking-the-future-of-plastics (accessed 2022–12–03).

[ref22] Annual Energy Outlook 2022, 2022 US Energy Information Administration. https://www.eia.gov/outlooks/aeo/IIF_carbonfee/.

[ref23] Inventory of U.S. Greenhouse Gas Emissions and Sinks, 2022. US EPA. https://www.epa.gov/ghgemissions/inventory-us-greenhouse-gas-emissions-and-sinks (accessed 2023–12–22).

[ref24] Emissions & Generation Resource Integrated Database (eGRID), 2022. US EPA. https://www.epa.gov/egrid.

[ref25] Cleansheet Solution. McKinsey & Company. https://www.mckinsey.com/capabilities/operations/how-we-help-clients/cleansheet (accessed 2022–12–03).

[ref26] Stocker T. F.; Qin D.; Plattner G.-K.; Tignor M. M.; Allen S. K.; Boschung J.; Nauels A.; Xia Y.; Bex V.; Midgley P. M. Climate Change 2013: The Physical Science Basis. Contribution of Working Group I to the Fifth Assessment Report of IPCC the Intergovernmental Panel on Climate Change; Intergovernmental Panel on Climate Change, 2014.

[ref27] Bioplastics Market Size & Share Analysis - Growth Trends & Forecasts (2023–2028), 2022. Mordor Intelligence. https://www.mordorintelligence.com/industry-reports/bioplastics-market (accessed 2023–09–28).

[ref28] Former Major OEM Product Designer with over 20 Years’ Experience + MCFM Experts Interview, McKinsey, 2022.

[ref29] Packaging in the US. Euromonitor. https://www.euromonitor.com/packaging-in-the-us/report (accessed 2022–10–20).

[ref30] wctLife-Cycle Inventory of Three Single-Serving Soft Drink Containers, 2009. PET Resin Association. https://legislature.maine.gov/testimony/resources/ENR20210322Augur132622006957081097.pdf.

[ref31] Jeswani H.; Krüger C.; Russ M.; Horlacher M.; Antony F.; Hann S.; Azapagic A. Life Cycle Environmental Impacts of Chemical Recycling via Pyrolysis of Mixed Plastic Waste in Comparison with Mechanical Recycling and Energy Recovery. Sci. Total Environ. 2021, 769, 144483 10.1016/j.scitotenv.2020.144483. 33486181

[ref32] Life Cycle Assessment of Example Packaging Systems for Milk, 2010. WRAP. https://kidv.nl/media/engelse_rapporten/life-cycle-assessment-of-example-packaging-systems-for-milk.pdf?1.2.2.

[ref33] Life Cycle Assessment of Supermarket Carrier Bags: A Review of the Bags Available in 2006, 2011. UK Environment Agency. https://assets.publishing.service.gov.uk/government/uploads/system/uploads/attachment_data/file/291023/scho0711buan-e-e.pdf (accessed 2022–10–17).

[ref34] Single-Use Plastic Bags and Their Alternatives: Recommendations from Life Cycle Assessments, 2020. United Nations Environment Programme. https://wedocs.unep.org/20.500.11822/31932.

[ref35] Environmental Footprint and Material Efficiency Support for Product Policy: Report on Benefits and Impacts/Costs of Options for Different Potential Material Efficiency Requirements for Dishwashers, 2015. European Comission. https://data.europa.eu/doi/10.2788/720546 (accessed 2022–10–18).

[ref36] Life Cycle Assessment of PVC Water and Sewer Pipe and Comparative Sustainability Analysis of Pipe Materials, 2017. Sustainable Solutions Corporation. https://www.uni-bell.org/files/Reports/Life_Cycle_Assessment_of_PVC_Water_and_Sewer_Pipe_and_Comparative_Sustainability_Analysis_of_Pipe_Materials.pdf (accessed 2022–10–17).

[ref37] Peer-Reviewed Life Cycle Inventory for the Production and Use of Installed Residential Piping Systems for Three House Layouts. Franklin Associates. https://cdn.ymaws.com/www.ppfahome.org/resource/resmgr/pdf/Peer_Reviewed_Pipe_Use_Phase.pdf (accessed 2022–10–17).

[ref38] Life Cycle Assessment of Spray Polyurethane Foam Insulation for Residential & Commercial Building Applications, 2020. SPFA. https://polo14.com/wp-content/uploads/2020/03/SPFA-LCA-Details.pdf.

[ref39] van der Velden N. M.; Patel M. K.; Vogtländer J. G. LCA Benchmarking Study on Textiles Made of Cotton, Polyester, Nylon, Acryl, or Elastane. Int. J. Life Cycle Assess. 2014, 19 (2), 331–356. 10.1007/s11367-013-0626-9.

[ref40] CARE 2019 Annual Report, 2020. Carpet America Recovery Effort (CARE). https://carpetrecovery.org/wp-content/uploads/2020/06/CARE-2019-Annual-Report-6-7-20-FINAL-002.pdf.

[ref41] Comparative Life Cycle Assessment of Tetra Pak Carton Packages and Alternative Packaging Systems for Beverages and Liquid Food on the European Market, 2020. Tetra Pak. https://www.tetrapak.com/content/dam/tetrapak/publicweb/uk/en/sustainability/2020-lca-tetra-pak-european-market.pdf.

[ref42] LCA of Beverage and Food Packaging in Australia and New Zealand, 2021. Tetra Pak. https://www.thinkstep-anz.com/assets/Whitepapers-Reports/Tetra-Pak-Oceania-LCA-Final-Report-Complete-Reformatted.pdf.

[ref43] Global Plastics Outlook: Policy Scenarios to 2060; OECD, 2022. 10.1787/aa1edf33-en.

[ref44] Bachmann M.; Zibunas C.; Hartmann J.; Tulus V.; Suh S.; Guillén-Gosálbez G.; Bardow A. Towards Circular Plastics within Planetary Boundaries. Nat. Sustain. 2023, 6, 599–610. 10.1038/s41893-022-01054-9.

[ref45] Hellweg S.; Benetto E.; Huijbregts M. A. J.; Verones F.; Wood R. Life-Cycle Assessment to Guide Solutions for the Triple Planetary Crisis. Nat. Rev. Earth Environ. 2023, 4 (7), 471–486. 10.1038/s43017-023-00449-2.

