# Supplementary material for: Replacing Plastics with Alternatives Is Worse for Greenhouse Gas Emissions in Most Cases
Source: Environ Sci Technol. 2024 Jan 31;58(6):2716–27. doi: 10.1021/acs.est.3c05191 (PMC10867844; doi:10.1021/acs.est.3c05191)
Supplement: Supplementary file 1 — es3c05191_si_001.pdf [file es3c05191_si_001.pdf]

Supporting information for

Replacing plastics with alternatives is worse for greenhouse gas emissions in most cases

Fanran Meng[1], Miguel Brandão[2*], Jonathan M Cullen[3*]

[1]Chemical & Biological Engineering, Faculty of Engineering, Sir Robert Hadfield Building, Mappin Street, Sheffield, S1 3JD, United Kingdom

[2]KTH Royal Institute of Technology, SE-100 44 Stockholm, Sweden

[3]Department of Engineering, University of Cambridge, Trumpington Street, Cambridge CB2 1PZ, United Kingdom

[*]Corresponding author: jmc99@cam.ac.uk; miguel.brandao@abe.kth.se

This PDF file includes:

Materials and Methods
Figs. S1 to S22
Tables S1 to S138

TABLE OF CONTENTS

1 GENERAL ASSUMPTIONS ... 6

2 SOFT DRINK CONTAINERS ... 10

2.1 Functional unit ... 10

2.2 Raw material acquisition, manufacture and transport ... 10

2.3 Retail transport ... 11

2.4 End-of-life disposition ... 11

2.5 Use phase/indirect impacts ... 12

2.6 Life cycle greenhouse gas emissions ... 13

2.7 Sensitivity analysis assumptions ... 13

2.8 Sensitivity analysis results ... 15

3 MILK CONTAINERS ... 18

3.1 Functional unit ... 18

3.2 Raw material acquisition, manufacture and transport ... 18

3.3 Retail transport ... 18

3.4 End-of-life disposition ... 18

3.5 Use phase/indirect impacts ... 19

3.6 Life cycle greenhouse gas emissions ... 19

3.7 Sensitivity analyses assumptions ... 20

3.8 Sensitivity analysis results ... 22

4 GROCERY BAGS ... 23

4.1 Functional unit ... 23

4.2 Raw material acquisition, manufacture and transport ... 23

4.3 Retail transport ... 23

4.4 End-of-life disposition ... 23

4.5 Use phase/indirect impacts ... 24

4.6 Life cycle greenhouse gas emissions ... 24

5 FRESH MEAT PACKAGING ... 26

5.1 Functional unit ... 26

5.2 Raw material acquisition, manufacture and transport ... 26

5.3 Retail transport ... 26

5.4 End-of-life disposition ... 26

5.5 Use phase/indirect impacts ... 27

5.6 Life cycle greenhouse gas emissions ... 28

6 WET PET FOOD PACKAGING ... 29

6.1 Functional unit ... 29

6.2 Raw material acquisition, manufacture and transport *29*
6.3 Retail transport *30*
6.4 End-of-life disposition *30*
6.5 Use phase/indirect impacts *31*
6.6 Life cycle greenhouse gas emissions *31*

7 INDUSTRIAL DRUMS 33
7.1 Functional unit *33*
7.2 Raw material acquisition, manufacture and transport *33*
7.3 Retail transport *33*
7.4 End-of-life disposition *33*
7.5 Use phase/indirect impacts *34*
7.6 Life cycle greenhouse gas emissions *34*

8 WATER CUPS 36
8.1 Functional unit *36*
8.2 Raw material acquisition, manufacture and transport *36*
8.3 Retail transport *36*
8.4 End-of-life disposition *37*
8.5 Use phase/indirect impacts *38*
8.6 Life cycle greenhouse gas emissions *38*

9 HANDSOAP BOTTLES 40
9.1 Functional unit *40*
9.2 Raw material acquisition, manufacture and transport *40*
9.3 Retail transport *40*
9.4 End-of-life disposition *41*
9.5 Use phase/indirect impacts *41*
9.6 Life cycle greenhouse gas emissions *42*

10 MUNICIPAL SEWER PIPES 43
10.1 Functional unit *43*
10.2 Raw material acquisition, manufacture and transport *43*
10.3 Retail transport *43*
10.4 End-of-life disposition *44*
10.5 Use phase/indirect impacts *44*
10.6 Life cycle greenhouse gas emissions *44*

11 RESIDENTIAL WATER PIPES 46
11.1 Functional unit *46*
11.2 Raw material acquisition, manufacture and transport *46*

11.3 Retail transport *46*
11.4 End-of-life disposition *46*
11.5 Use phase/indirect impacts *47*
11.6 Life cycle greenhouse gas emissions *47*
12 BUILDING INSULATION 49
12.1 Functional unit/use phase scenario *49*
12.2 Raw material acquisition, manufacture and transport *49*
12.3 Retail transport *49*
12.4 End-of-life disposition *49*
12.5 Use phase/indirect impacts *50*
12.6 Life cycle greenhouse gas emissions *50*
13 FURNITURE SET 52
13.1 Functional unit *52*
13.2 Raw material acquisition, manufacture and transport *52*
13.3 Retail transport *52*
13.4 End-of-life disposition *52*
13.5 Use phase/indirect impacts *53*
13.6 Life cycle greenhouse gas emissions *53*
14 AUTOMOTIVE FUEL TANKS 55
14.1 Functional unit *55*
14.2 Raw material acquisition, manufacture and transport *55*
14.3 Retail transport *55*
14.4 End-of-life disposition *56*
14.5 Use phase/indirect impacts *56*
14.6 Life cycle greenhouse gas emissions *56*
15 AUTOMOTIVE ELECTRIC-VEHICLE BATTERY PACK TOP ENCLOSURES 58
15.1 Functional unit *58*
15.2 Raw material acquisition, manufacture and transport *58*
15.3 Retail transport *58*
15.4 End-of-life disposition *59*
15.5 Use phase/indirect impacts *59*
15.6 Life cycle greenhouse gas emissions *59*
16 T-SHIRTS 61
16.1 Functional unit *61*
16.2 Raw material acquisition, manufacture and transport *61*
16.3 Retail transport *61*

16.4 End-of-life disposition.....61
16.5 Use phase/indirect impacts.....62
16.6 Life cycle greenhouse gas emissions.....62
17 CARPETS.....64
17.1 Functional unit.....64
17.2 Raw material acquisition, manufacture and transport.....64
17.3 Retail transport.....64
17.4 End-of-life disposition.....65
17.5 Use phase/indirect impacts.....65
17.6 Life cycle greenhouse gas emissions.....65
References.....66

1 GENERAL ASSUMPTIONS

(1) Assume US landfill vs. waste-to-energy (WtE) mix of 80% vs. 20%, unless otherwise stated.

(2) End-of-life recycling refers to mechanical recycling, unless otherwise stated.

(3) Average US energy grid emission factor is 0.368 $kgCO_{2eq}/kWh$

(4) Retail transport mileage assumption (Table S1)

Table S1 Retail transport mileage assumption [1]

	Mileage	Comment
Plastics	497	
Metals	331	
Glass	356	
Office paper	257	Proxy paper material for milk carton and paper bag
Corrugated container	675	Proxy paper material for paper cup
Wood	246	
Fibreglass	356	

In selecting applications for our analysis, we first segmented global plastic demand into sectors. In 2020, global plastic demand was approximately 300 million metric tonnes (MMT), of which the top five sectors with the highest plastic consumption - packaging, building and construction, consumer goods, automotive, and textiles - comprised of 270 MMT or around 90% of total volume (Figure S1). We evaluated plastics applications in those sectors and compared with non-plastic alternatives in each category in terms of GHG emissions.

For some application categories, such as automotive interiors, caps and closures, appliances, and electronics, there are few, if any, plastic alternatives currently present in the market. These categories constitute approximately 45 MMT of total plastics volume. We have excluded these alternatives for which there are few alternatives to plastic: agricultural packaging and caps and closures in the packaging sector, appliances and electronics in the consumer-goods sector, and interior and exterior in the automotive sector. In addition, we excluded “other” (which includes floor, fixture, liner and frame applications) in the building-and-construction sector, chassis and electronics and accessories in the automotive sector, as well as household and furnishing and “other” in the textile sector.

For the remaining categories where there is a realistic choice between plastic and non-plastic alternatives, such as rigid food packaging, pipes, and automotive powertrains, we selected one or more representative applications. The choice of which products and materials to assess was based on market share (particularly in the United States) of products used at scale today and excluded nascent or niche solutions.

In some categories, there were applications with majority market shares; for example, in building insulation, we compared polyurethane with glass fibre because they cover approximately 80% of new-build construction. However, in many other categories, there is no dominant application, and we selected examples deemed representative, such as a refillable soap bottle as an example of rigid non-food packaging. While a soap bottle does not represent the entire sector of rigid non-food packaging, it

provides an example of the space. In the automotive sector, we chose to consider the fuel tanks in hybrid vehicles, rather than in ICEV, as the hybrid market is expected to grow in the coming years relative to that of ICEV, and fuel tank sizes on hybrids are similar to ICEV fuel tanks.

We cover a range of both plastics and non-plastic materials. We compare plastic to non-bio-based alternatives such as steel, glass, aluminium, glass fibre, copper, concrete, and ductile iron, and bio-based alternatives such as paper, wood, cotton, and wool. Necessarily, other materials and comparisons are excluded due to their low market share or the availability of reliable use data. For example, we chose to focus on plastic and paper grocery bags, excluding reusable grocery bags due to the wide array of volumes and materials used and a lack of reliable reuse data that critically impacts the life cycle of these alternatives. We also chose to exclude compostable and biodegradable alternatives as, while we acknowledge that they may be promising for reducing GHG emissions, they currently occupy a small fraction of the market, at approximately 2 million tonnes annually or less than 1% of the plastics market. Importantly, none of the applications were chosen to favour plastics. Instead, they were selected to cover the full range of plastic uses before performing any analysis, as it was unknown whether plastics would be the most favourable material a priori.

Finally, we included two additional applications where plastic competes with plastic-enabled alternatives: water cups and milk containers. In both applications, the alternative to plastic is a mix of approximately 80% paper and 20% plastic. Although this is not a pure comparison between plastic and non-plastic materials, we have included them, as these applications were deemed to be good examples to be included.

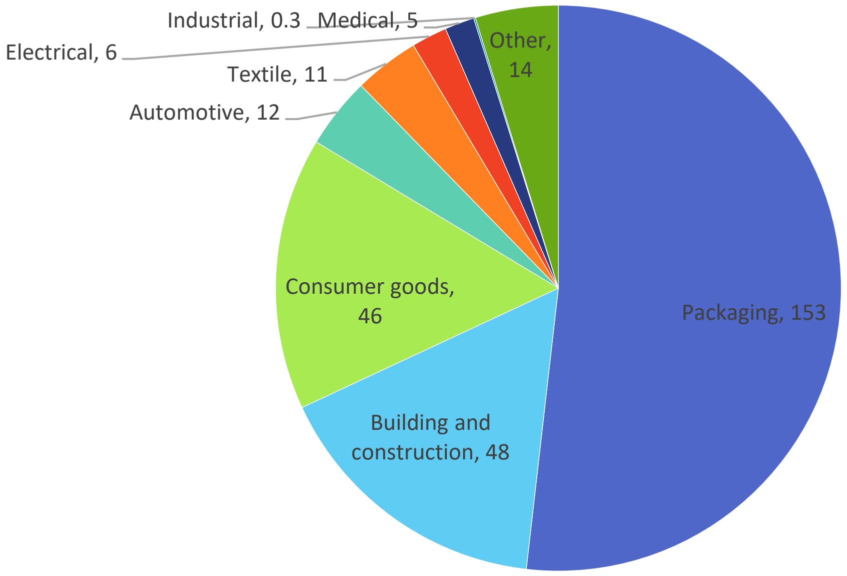

Figure S1 Global plastic demand in 2020 (MMT).

Table S 2. Selected alternatives for the different plastic applications assessed. Acronyms: EPS (expanded polystyrene), HDPE (high density polyethylene), PET (polyethylene terephthalate), PEX (cross-linked polyethylene), PP (polypropylene), PU (polyurethane), (PVC (polyvinyl chloride).

Sector and	Plastic vs. non-plastic	Functional unit

categories	alternative applications	
**Packaging**		
Rigid food packaging	Soft drinks: PET vs. glass bottle vs. aluminum can	2957 liter (100,000 oz) soft drinks
	Milk: HDPE milk bottle vs. gable top carton	1.89 liter (64 oz) of refrigerated milk
Flexible non-food packaging	Grocery bag: HDPE vs. paper bag	Carrying capacity for 1,040 grocery items purchased from a supermarket and transported to consumer’s home (assume 20 items purchased per week for a household of four for one year)
Flexible food packaging	Pet food: multi-layer pouch vs. aluminum vs. steel can	2.95 liter (1,000 oz) of wet pet food
	Fresh meat packaging: EPS foam tray + PVC film vs. butcher paper	Packaging capacity for 454 kg (1000 lbs) of pork (assuming 1 lb of pork in each package)
Industrial packaging	Industrial drums: HDPE vs. steel drum	One 250 liter (55 gallon) drum per 10-year usage
Rigid non-food packaging	Hand soap bottle: HDPE vs glass hand soap bottle	One-year liquid hand soap consumption for a household of four (assume each person washes hands three times per day)
**Building and construction**		
Pipe application	Municipal sewer pipes: PVC vs. concrete vs. ductile iron	30 meter (100 feet) of sewer main pipe for usage of 100 years; consider both gravity (38-cm (15-inch) common) and force main (30-cm (12-inch) common) pipes
	Residential water pipes: PEX vs. copper	Hot and cold-water distribution pipe system for a 261 m^2 (2,811 square foot) house (excluding watermain pipes and main wastewater line), with usage modelled in the clustered use phase (peaks of usage in morning and night for family of 4); lifetime of 50 years for pipes
Insulation	Building insulation: PU vs. fiberglass	Insulation required to fill a 2x6 cavity on a 233 m^2 (2512 square foot) two-story wood house, Richmond, VA, R=49 insulation in attic, R=20 insulation in walls, gas-fired air furnace/AC, gas-fired water heater, life of 75 years
**Consumer goods**		
Consumer durable	Furniture: PP vs. steel vs. wood furniture set	Furniture set of one square table and four chairs, with lifespan of 10 years
Consumer non-durable	Water cups: EPS vs. PP vs. PET vs. paper vs. reusable glass cup	10,000 oz (283 kg) water served at cafe in 500 20 oz (0.57 kg) cups
**Automotive**		
Powertrain	Hybrid vehicle fuel tank: HDPE vs. steel	One fuel tank for a four-seat hybrid sedan with a lifespan of 200,000 miles (~322,000 km); assume no difference in weight of vehicle in operation other than fuel tank material
	Automotive electric vehicle battery pack top enclosure: PP vs. steel	One battery pack top enclosure for a compact BEV SUV with a lifespan of 200,000 miles (~322,000 km); assume no difference in weight of vehicle in operation other than battery top enclosure

**Textile**		
Apparel	T-shirt: PET vs. cotton	1000 kg of fibers, regardless of discrete numbers of shirts (because volume of fiber and thus raw material varies significantly with variables like t-shirt size and brand)
Floor cover	Carpet: Synthetic (nylon) vs. wool	A carpet with face weight of 40 oz/square yard (1356 gram/m^2) and density of 3,100 oz/inch (3,460 kg/m)

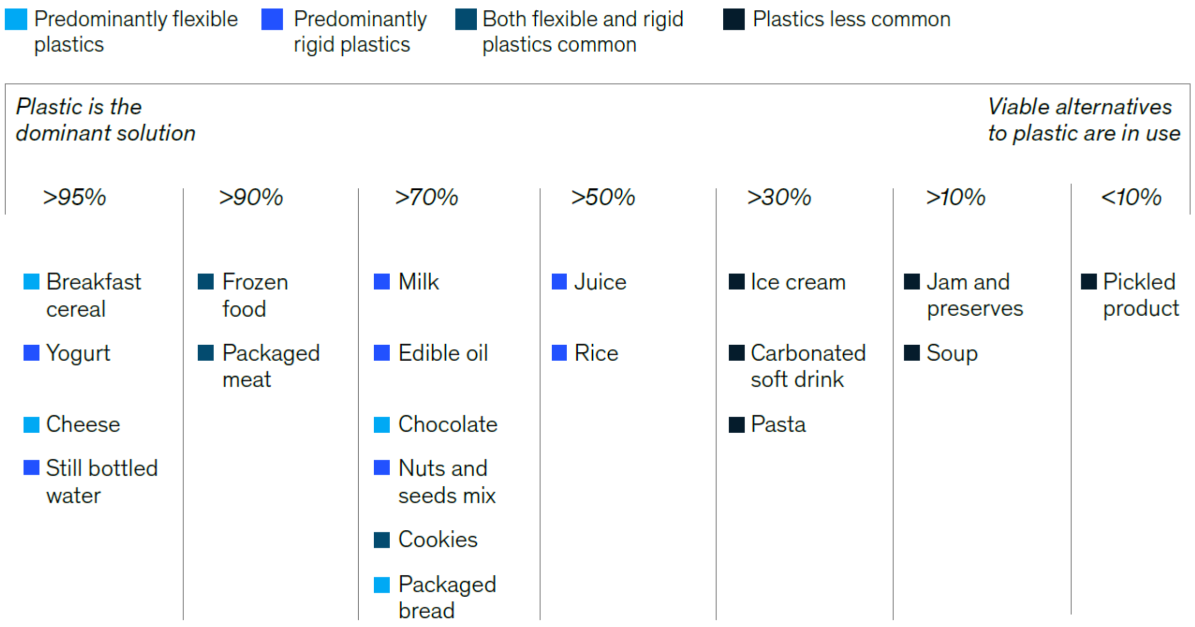

Figure S 2 Products with plastic packaging showing viable alternatives to plastic.

2 SOFT DRINK CONTAINERS

2.1 Functional unit

100,000 oz soft drinks packaged in:

- 5,000 20 oz PET bottles,
- 8,334 12 oz Aluminium cans, or
- 8,334 12 oz glass bottles

Table S3 Soft drink container US 2020 scenario: reference flows and EoL disposition

	Reference flow*		EoL pathway (%)			
	Quantity	Weight (kg)	Recycling	WtE	Landfill	Reuse
PET bottle	5,000	140	29.0	14.2	56.8	0.0
Aluminium can	8,333	125	50.0	10.0	40.0	0.0
Glass bottle	8,333	1,910	40.0	11.9	47.7	0.4

* for 100,000 oz functional unit

2.2 Raw material acquisition, manufacture and transport

Table S4 Weight of one container (source: actual measurement)

	Size, oz	Weight, g
PET bottle	20	27
Aluminium can	12	15

Glass bottle	12	230

Table S 5 GHG emissions for this life cycle stage

Input	$kgCO_{2eq}$ per functional unit	Source	Comment
PET bottle	441	EPA WARM, Ecoinvent	Include emissions from converting plastic pellets to bottle
Aluminium can	1,510	EPA WARM	
Glass bottle	1,312	EPA WARM	

2.3 Retail transport

Table S6 GHG emissions for this life cycle stage [1]

Input	$kgCO_{2eq}$ per functional unit	Comment
PET bottles	6	Average miles per shipment is 497
Aluminium cans	4	Average miles per shipment is 331
Glass bottles	63	Average miles per shipment is 356

2.4 End-of-life disposition

Table S7 GHG emissions for recycling [1,2]

Input	Recycling rate, %	$kgCO_{2eq}$ per functional unit	Comment
PET bottle	29	-44	Mass allocation ratios from EPA model
Aluminium can	50	-628	Mass allocation ratios from EPA model
Glass bottle	40	-247	Mass allocation ratios from EPA model

Table S8 GHG emissions for landfill [1]

Input	Landfill rate, %	$kgCO_{2eq}$ per functional unit	Comment
PET bottle	57	1.7	Assume US landfill vs. WtE mix of 80% vs. 20% for non- recycled
Aluminium can	40	1.1	Assume US landfill vs. WtE mix of 80% vs. 20% for non- recycled
Glass bottle	48	20.0	Assume US landfill vs. WtE mix of 80% vs. 20% for non- recycled

Table S9 GHG emissions for WtE [1]

Input	Incineration/waste-to-energy rate, %	$kgCO_{2eq}$ per functional unit	Comment
PET bottle	14	26.6	Assume US landfill vs. WtE mix of 80% vs. 20% for non-recycled
Aluminium can	10	0.55	Assume US landfill vs. WtE mix of 80% vs. 20% for non-recycled
Glass bottle	12	3.9	Assume US landfill vs. WtE mix of 80% vs. 20% for non-recycled

Table S10 GHG emissions for reuse

Input	Recycling rate, %	$kgCO_{2eq}$ per functional unit	Source
PET bottle	0		
Aluminium can	0		
Glass bottle	0.4	-3.2	Container Recycling Institute, EPA WARM, IEA World Energy Outlook

2.5 Use phase/indirect impacts

Table S 11 Breakage and spoilage rates of bottles

	Input	%	Source	Comment
Breakage rate	Glass bottle	2	Beer industry report, Expert interview	
Spoilage rate	PET bottle	1	Expert interview	Shelf life for PET ~13 weeks vs. Aluminium and glass ~52 weeks

2.6 Life cycle greenhouse gas emissions

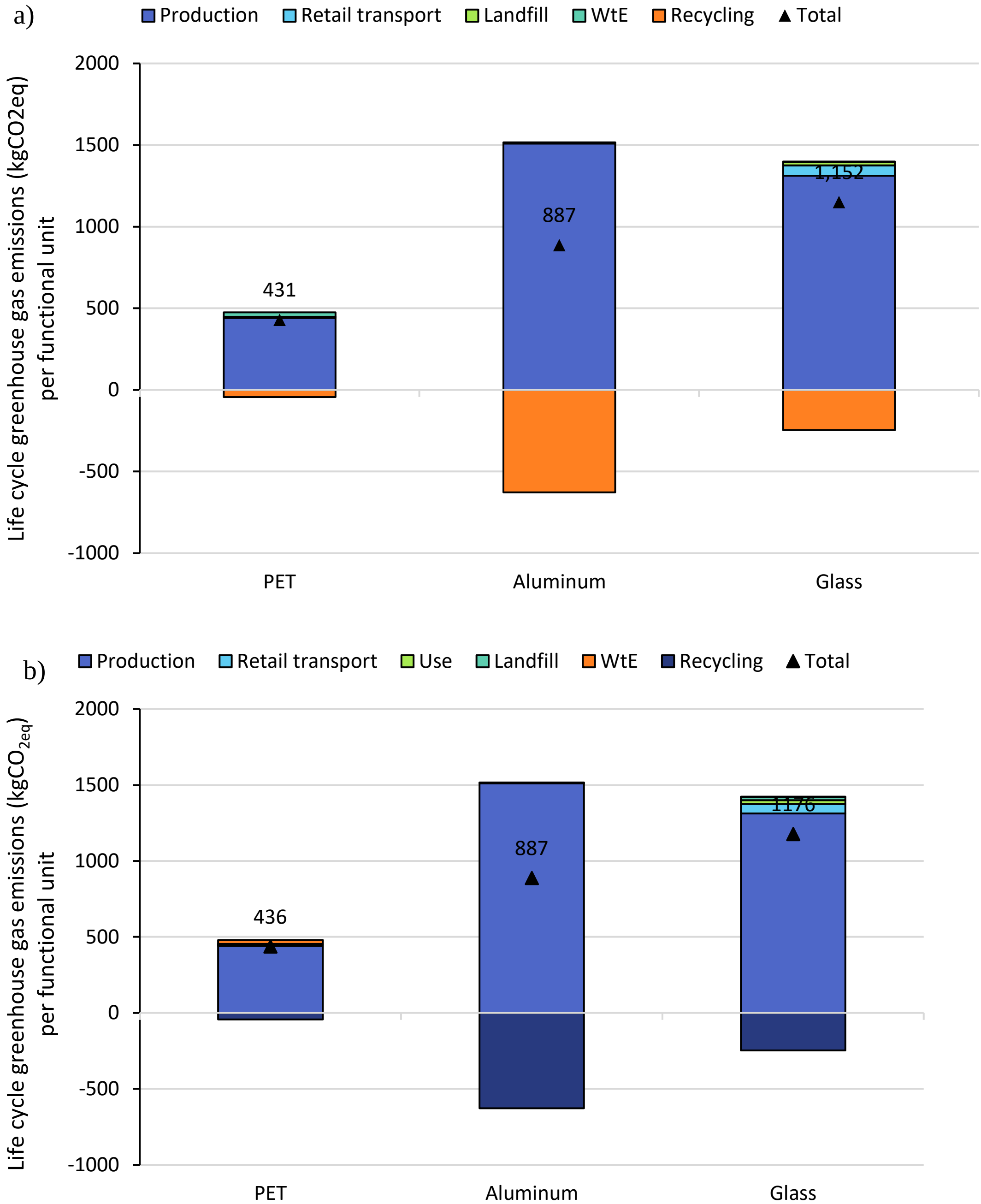

Figure S3 a) Life cycle GHG emissions excluding use phase emissions and b) life cycle GHG emissions including use phase emissions ($kgCO_{2eq}$ per 100,000 oz of soft drink). The production stage includes emissions from raw material acquisition, manufacture as well as adjustments made to the functional unit for additional production of container required to compensate for soilage and breakage.

2.7 Sensitivity analysis assumptions

Table S12 Industrial energy mix [3,4]

	US 2020 (%)			Europe 2020 (%)			China (%)			Global 2020 (%)			US 2050 base[1] (%)			US 2050 best[1] (%)		
	C	G	R	C	G	R	C	G	R	C	G	R	C	G	R	C	G	R
PET bottle	8	77	15	10	45	45	59	10	31	27	44	29	1	31	68	0	0	100
Aluminium can[2]	5	46	49	6	27	67	92	2	6	16	26	58	1	21	78	0	0	100
PET bottle	13	62	25	9	52	39	58	12	30	30	37	33	1	31	68	0	0	100

Note: C: coal; G: natural gas and oil; R: renewables and nuclear

Table S13 End-of-life disposition mix [2] (Source: EPA Advancing Sustainable Materials Management (2020), Expert interview, industry reports)

	Recycling (%)	Landfill (%)	Waste-to-Energy (%)	Reuse (%)
US 2020				
PET bottle	29	57	14	0
Aluminium can	50	40	10	0
Glass bottle	40	48	12	0.4
Europe 2020				
PET bottle	56	13	31	0
Aluminium can	85	5	11	0
Glass bottle	78	5	12	5
China				
PET bottle	68	6	26	0
Aluminium can	80	10	10	0
Glass bottle	20	40	40	1
Global 2020				
PET bottle	42	35	23	0
Aluminium can	69	19	12	0
Glass bottle	32	40	27	1
US 2050				
PET bottle	54	18	28	0
Aluminium can	75	10	15	0
Glass bottle	60	16	24	0.4

[1] Assume boilers are fully electrified in 2050

[2] Majority of renewables in aluminium production is hydropower in Europe and US, while aluminum production in China is coal intensive

US 2050 best				
PET bottle	71	9	20	0
Aluminium can	90	3	7	0
Glass bottle	90	3	7	0.4

Table S14 Commercial battery electric vehicle (BEV) vs. internal combustion engine (ICE) mix [4]

	US 2020	Europe 2020	China 2020	Global 2020	US 2050 base	US 2050 best
PET bottle	0% BEV<br>100% ICE	0% BEV<br>100% ICE	0% BEV<br>100% ICE	0% BEV<br>100% ICE	52% BEV<br>48% ICE	80% BEV<br>20% ICE
Aluminium can	0% BEV<br>100% ICE	0% BEV<br>100% ICE	0% BEV<br>100% ICE	0% BEV<br>100% ICE	52% BEV<br>48% ICE	80% BEV<br>20% ICE
Glass bottle	0% BEV<br>100% ICE	0% BEV<br>100% ICE	0% BEV<br>100% ICE	0% BEV<br>100% ICE	52% BEV<br>48% ICE	80% BEV

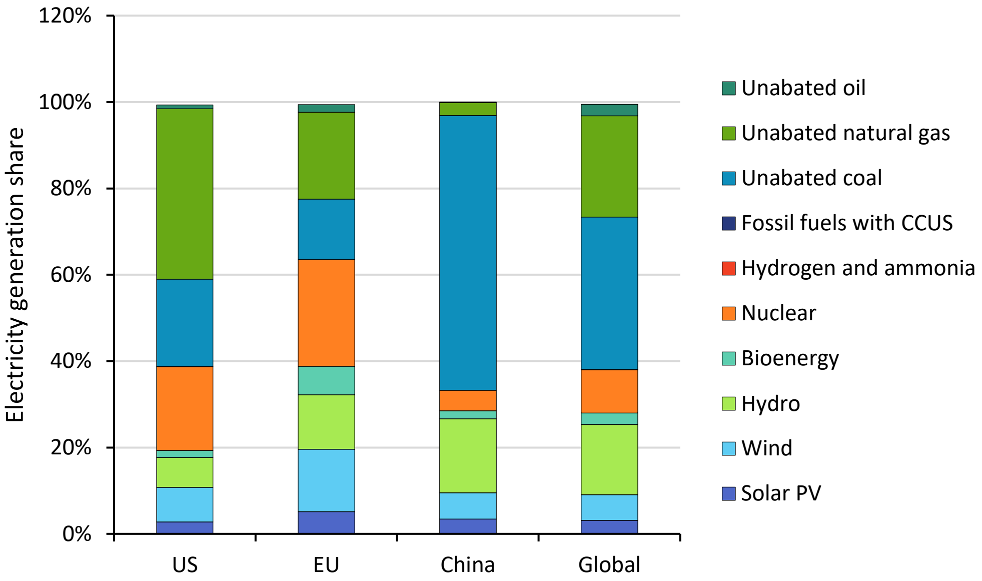

Figure S4 2020 electricity mix in the US, EU, China and Global [3]

2.8 Sensitivity analysis results

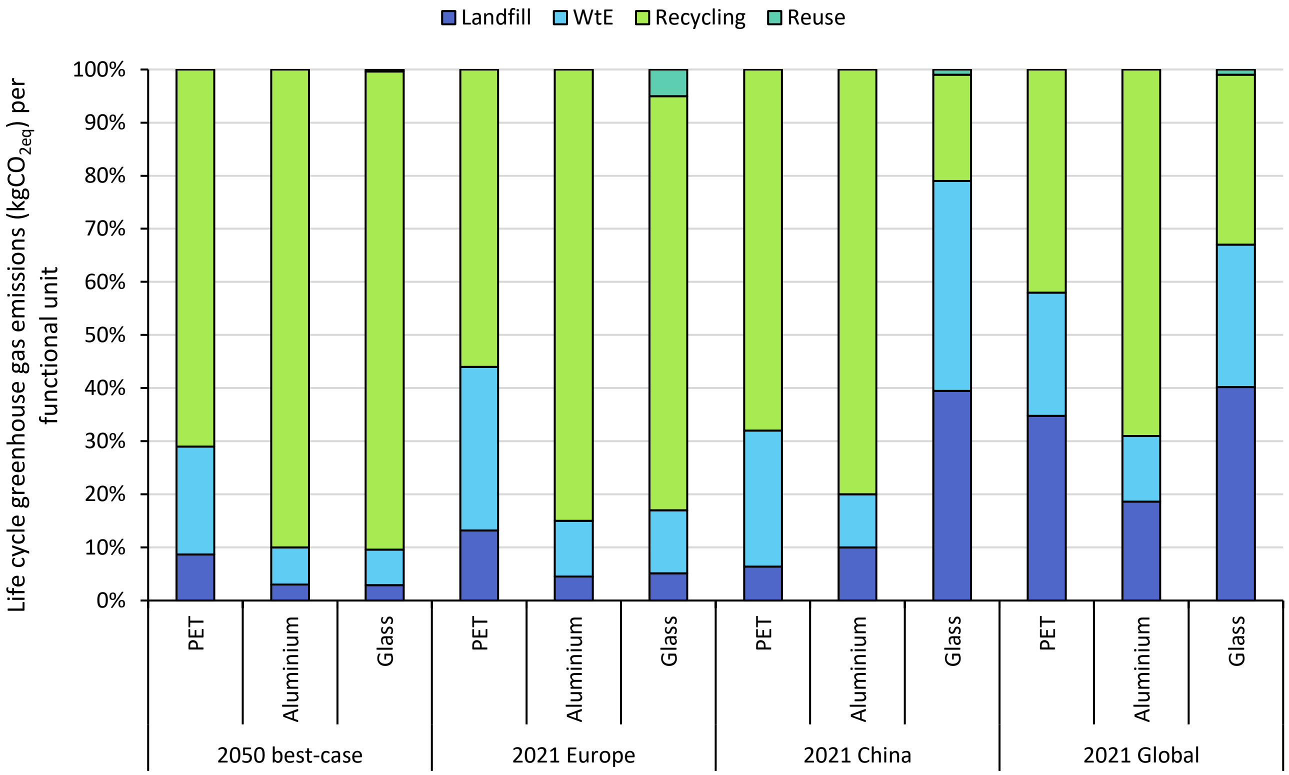

Figure S5 2020 EoL disposition in Western Europe, China and Global and US 2050 best-case scenario

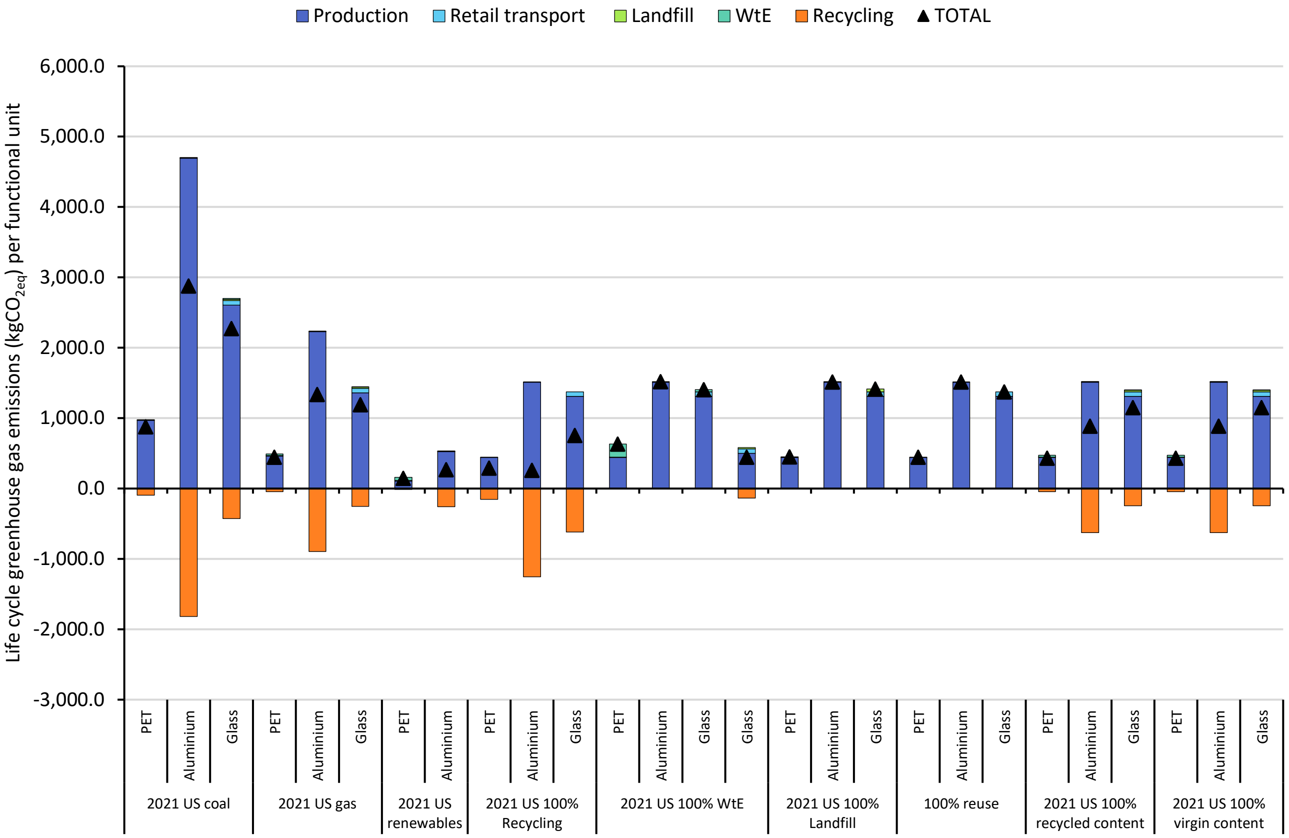

Figure S 6 Additional soft drink containers regional 2020 and US 2050 scenarios.

3 MILK CONTAINERS

3.1 Functional unit

1.89 L/64 oz of refrigerated milk packaged in HDPE bottle and gable top carton (80% paper and 20% LDPE)

3.2 Raw material acquisition, manufacture and transport

Table S15 Weight of one container (Source: actual measurement)

	Specification	Weight, g	Comment
HDPE bottle	64 oz	47	Include HDPE cap (~2g)
Gable top carton	64 oz (80% paper cardboard, 20% LDPE)	76	Include HDPE cap (~2g)

Table S16 Milk container US 2020 scenario: reference flows and EoL disposition

	Reference flow*		EoL pathway (%)			
	Quantity	Weight (g)	Recycling	WtE	Landfill	Reuse
HDPE bottle	1	47	29.3	14.1	56.6	0.0
Gable top carton	1	76	0.0	20.0	80.0	0.0

* for 64 oz functional unit

Table S17 GHG emissions [1,5]

	gCO_2eq per functional unit
HDPE bottle	119
Gable top carton	99

3.3 Retail transport

Table S18 GHG emissions for this life cycle stage [1]

	gCO_2eq per functional unit	Comment
HDPE bottle	2	Average miles per shipment is 497 for plastics
Gable top carton	2	Average miles per shipment is 497 for plastics and 257 for paper

3.4 End-of-life disposition

Table S19 GHG emissions for recycling

	Recycling rate, %	g CO_2e per functional unit	Source	Comment

HDPE bottle	29	-11	EPA Advancing Sustainable Materials Management (2020)	US 2018 data Mass allocation ratios from EPA model
Gable top carton	0	0	Expert interview	No appreciable recycling in the US today

Table S20 GHG emissions for landfill

	Landfill rate, %	g CO_2e per functional unit	Comment
HDPE bottle	57	1	Assume US landfill vs. WtE mix of 80% vs. 20% for non-recycled material
Gable top carton	80	51	Assume US landfill vs. WtE mix of 80% vs. 20% for non-recycled material

Table S21 GHG emissions for landfill

	Incineration/waste-to- energy rate, %	g CO_2e per functional unit	Comment
HDPE bottle	14	9	Assume US landfill vs. WtE mix of 80% vs. 20% for non-recycled material
Gable top carton	20	-2	Assume US landfill vs. WtE mix of 80% vs. 20% for non-recycled material

Table S22 GHG emissions for advanced recycling

	Recycling rate, %	g CO_2e per functional unit	Comment
HDPE bottle	0	0	No at-scale advanced recycling currently
Gable top carton	0	0	No at-scale advanced recycling currently

3.5 Use phase/indirect impacts

No measurable Use phase/indirect impacts since no appreciable difference in shelf life

3.6 Life cycle greenhouse gas emissions

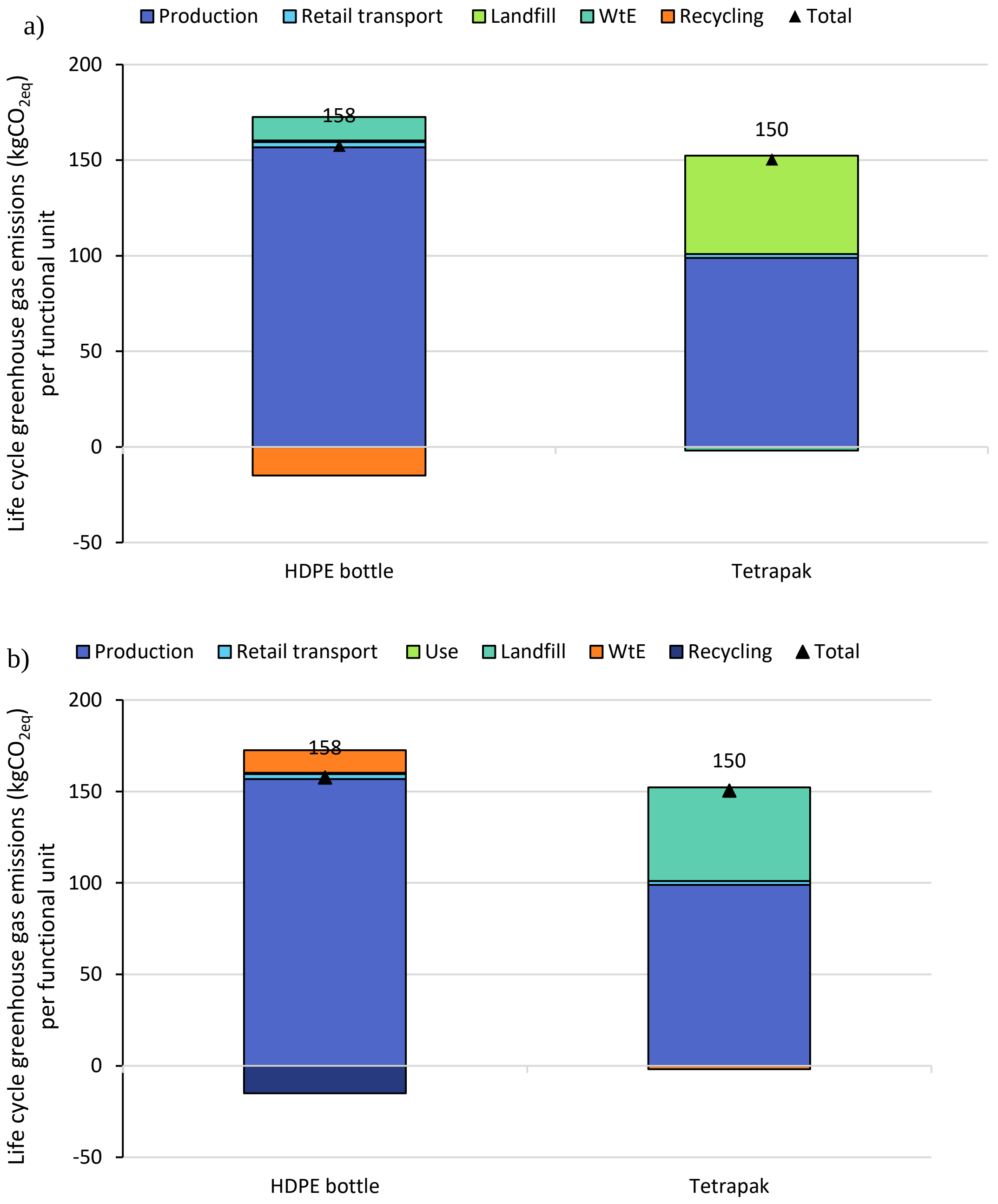

Figure S7 a) Life cycle GHG emissions excluding use phase emissions and b) life cycle GHG emissions including use phase emissions ($kgCO_{2eq}$ per 64 oz of refrigerated dairy milk). The production stage includes emissions from raw material acquisition, manufacture. HDPE bottles and gable top cartons have a similar shelf life and overall climate-change impact.

3.7 Sensitivity analyses assumptions

Table S23 Industrial energy mix [3,4]. Note: C=coal, G=natural gas and oil, R=renewables.

	US 2020 (%)			Europe 2020 (%)			China (%)			Global 2020 (%)			US 2050 base[3] (%)			US 2050 best[1] (%)[3]		
	C	G	R	C	G	R	C	G	R	C	G	R	C	G	R	C	G	R
HDPE bottle	8	77	15	10	45	45	59	10	31	27	44	29	1	31	68	0	0	100
Gable top carton	13	62	25	9	52	39	58	12	30	30	37	33	1	31	68	0	0	100

Table S24 End-of-life disposition mix[2,6] (Source: EPA Advancing Sustainable Materials Management (2020), Expert interview, industry reports)

	Recycling (%)	Landfill (%)	Waste-to-Energy (%)	Advanced recycling (%)
US 2020				
HDPE bottle	29	57	14	0
Gable top carton	0	80	20	0
Europe 2020				
HDPE bottle	67	10	23	0
Gable top carton	48	16	36	0
China				
HDPE bottle	74	13	13	0
Gable top carton	10	45	45	0
Global 2020				
HDPE bottle	48	31	21	0
Gable top carton	26	44	30	0
US 2050				
HDPE bottle	65	7	11	17
Gable top carton	60	16	24	0
US 2050 best				
HDPE bottle	81	1	1	17
Gable top carton	80	6	14	0

Table S25 Commercial BEV vs. ICE mix [4]

	US 2020	Europe 2020	China 2020	Global 2020	US 2050 base	US 2050 best
HDPE bottle	0% BEV<br>100% ICE	0% BEV<br>100% ICE	0% BEV<br>100% ICE	0% BEV<br>100% ICE	52% BEV<br>48% ICE	80% BEV<br>20% ICE
Gable top	0% BEV	0% BEV	0% BEV	0% BEV	52% BEV	80% BEV

[3] Assume boilers are fully electrified in 2050

carton	100% ICE	100% ICE	100% ICE	100% ICE	48% ICE	20% ICE

3.8 Sensitivity analysis results

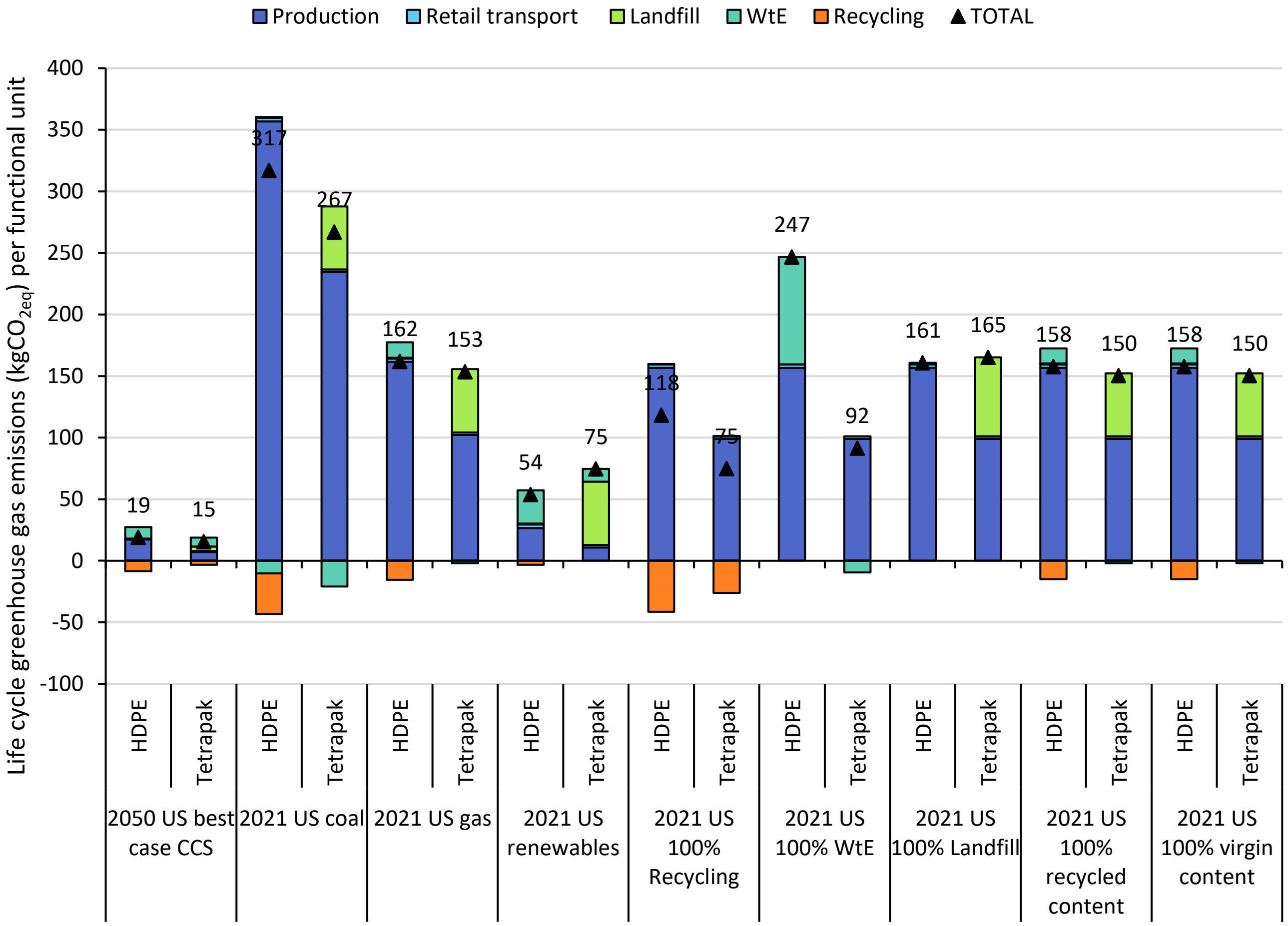

Figure S 8Additional milk container regional 2020 and US 2050 scenarios.

4 GROCERY BAGS

4.1 Functional unit

Carrying capacity for 1,040 grocery items purchased from a supermarket and transported to consumer's home (assume 20 items purchased per week for a household of four for one year)

4.2 Raw material acquisition, manufacture and transport

Table S26 Weight of one container [7]

	Items per bag	Weight per bag, g
HDPE bag	6	8
Paper bag	7	55

Table S27 Grocery bag US 2020 scenario: reference flows and EoL disposition

	Reference flow*		EoL pathway (%)			
	Quantity	Weight (kg)	Recycling	WtE	Landfill	Reuse
HDPE bags	177	1.5	8.0	18.4	73.6	0.0
Paper bags	140	7.7	21.0	15.8	63.2	0.0

* for 1,040 items of grocery per year (assuming weekly shopping of 20 items for a year). For both types of bags, 100% virgin production was assumed.

Table S28 GHG emissions

	$kgCO_{2eq}$ per functional unit	Source	Comment
HDPE bag	3	EPA WARM,<br>Ecoinvent	Include conversion emissions from plastic pellets to final product
Paper bag	10	EPA WARM,<br>Ecoinvent, EUROSAC	Include conversion emissions to final<br>product (e.g., forming, gluing, cutting, and pressing)

4.3 Retail transport

Table S29 GHG emissions for retail transport [1]

	$kgCO_{2eq}$ per functional unit	Comment
HDPE bag	0.06	Average miles per shipment is 497
Paper bag	0.17	Average miles per shipment is 257

4.4 End-of-life disposition

Table S30 GHG emissions for recycling [2]

	Recycling rate, %	$kgCO_{2eq}$ per functional unit	Comment
HDPE bag	8	-0.10	Mass allocation ratios from EPA model
Paper bag	21	0.34	Mass allocation ratios from EPA model

Table S31 GHG emissions for landfill

	Landfill rate, %	$kgCO_{2eq}$ per functional unit	Comment
HDPE bag	74	0.02	Assume US landfill vs. WtE mix of 80% vs. 20% for non-recycled material
Paper bag	63	5.26	Assume US landfill vs. WtE mix of 80% vs. 20% for non-recycled material

Table S32 GHG emissions for WtE

	Incineration/waste-to-energy rate, %	$kgCO_{2eq}$ per functional unit	Comment
HDPE bag	18	0.38	Assume US landfill vs. WtE mix of 80% vs. 20% for non-recycled material
Paper bag	16	-0.63	Assume US landfill vs. WtE mix of 80% vs. 20% for non-recycled material

4.5 Use phase/indirect impacts

Table S33 Breakage rate (Source: expert interview)

	%	Comment
Paper bag	50	Use double bagging rate as proxy for breakage rate
HDPE bag	20	Use double bagging rate as proxy for breakage rate

4.6 Life cycle greenhouse gas emissions

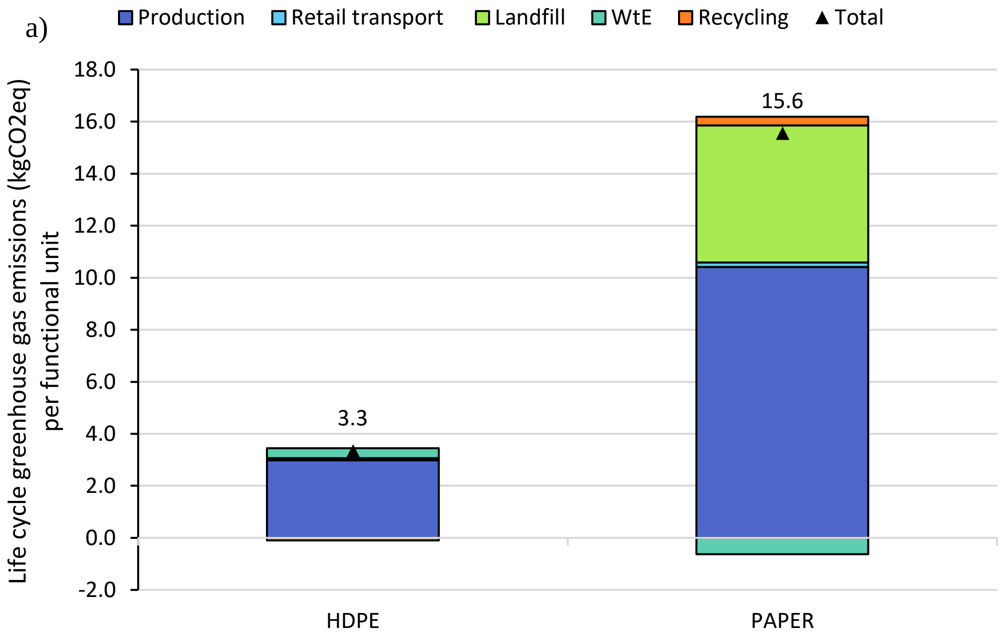

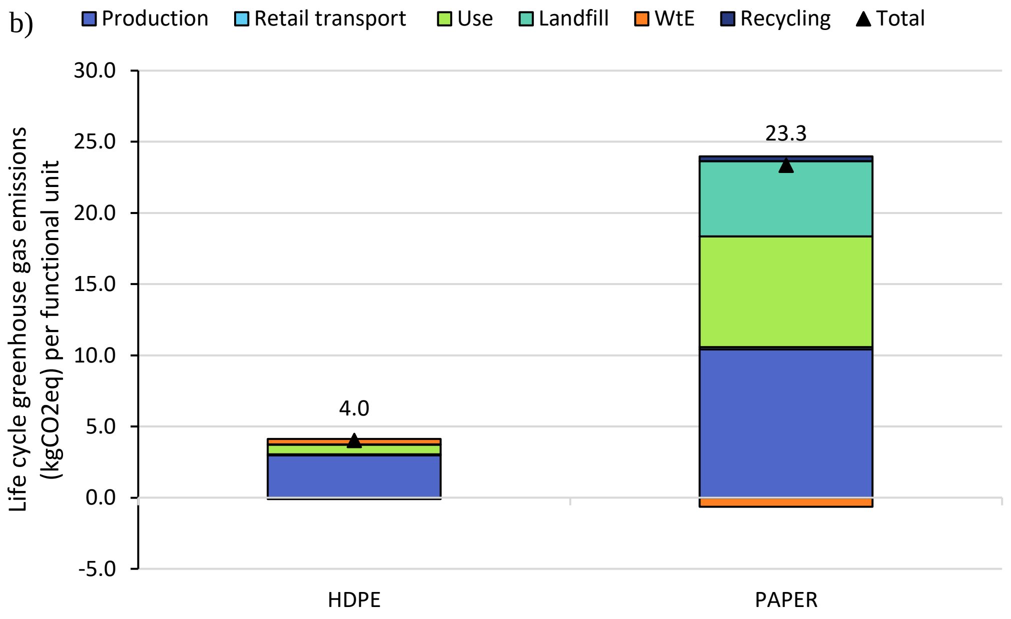

Figure S 9 a) Life cycle GHG emissions excluding use phase emissions and b) life cycle GHG emissions including use phase emissions ($kgCO_{2eq}$ per 1,040 items of grocery). The production stage includes emissions from raw material acquisition, manufacture. Climate impacts from deforestation, landfill carbon storage and paper combustion are excluded.

5 FRESH MEAT PACKAGING

5.1 Functional unit

Packaging capacity for 1000 lbs of pork (assume 1 lb of pork in each package)

5.2 Raw material acquisition, manufacture and transport

Table S34 Weight of one container

	Weight, g	Source	Comment
Foam tray + PVC film	10.8	National Life Cycle Carbon Footprint Study for Production of US Swine	
Butcher paper	7.6	[8]	40# white butcher paper (8.5 x 8.5 inches)

Table S35 Fresh meat packaging US 2020 scenario: reference flows and EoL disposition

	Reference flow*		EoL pathway (%)			
	Quantity	Weight (kg)	Recycling	WtE	Landfill	Reuse
EPS tray and PVC film	1,000	10.8	0.0	20.0	80.0	0.0
Butcher paper	1,000	15.2	0.0	20.0	80.0	0.0

* for 1,000 lb of pork. 1000 PS foam trays each with the volume of 1L and holds 1 lb pork; 1000 butcher paper that holds 1lb pork per paper. Two butcher papers are needed to wrap 1lb of meat. Incremental spoilage rate of paper vs. plastic is assumed at 2-5% (shelf life of 3-4 days for paper vs. 5 days for plastic; 90% in refrigerator vs 10% pork stored in freezer).

Table S36 GHG emissions for this life cycle stage

Input	$kgCO_{2eq}$ per functional unit	Source	Comment
EPS foam tray and PVC film	36	EPA WARM, Ecoinvent	Include absorption pad
Butcher paper	11	EPA WARM, Ecoinvent, EUROSAC	Assume two butcher papers per 1 lb of pork, per Expert interview

5.3 Retail transport

Table S37 GHG emissions for this life cycle stage [1]

	$kgCO_{2eq}$ per functional unit	Comment
EPS foam tray and PVC film	0.5	Average miles per shipment is 497
Butcher paper	0.3	Average miles per shipment is 257

5.4 End-of-life disposition

Table S38 Recycling rate and associated GHG emissions (Source: expert interview)

Input	Recycling rate, %	$kgCO_{2eq}$ per functional unit	Comment
EPS foam tray and PVC film	0	0	No at-scale recycling of EPS and PVC film currently in the US
Butcher paper	0	0	Butcher paper likely contaminated after use

Table S39 Landfill rate and associated GHG emissions

	Landfill rate, %	$kgCO_{2eq}$ per functional unit	Comment
EPS foam tray and PVC film	80	1.3	Assume US landfill vs. WtE mix of 80% vs. 20% for non-recycled material
Butcher paper	80	13.1	Assume US landfill vs. WtE mix of 80% vs. 20% for non-recycled material

Table S40 WtE rate and associated GHG emissions

	Incineration/waste-to-energy rate, %	$kgCO_{2eq}$ per functional unit	Comment
EPS foam tray and PVC film	20	3.3	Assume US landfill vs. WtE mix of 80% vs. 20% for non-recycled material
Butcher paper	20	-1.6	Assume US landfill vs. WtE mix of 80% vs. 20% for non-recycled material

5.5 Use phase/indirect impacts

Table S41 Spoilage rate at consumer stage (Source: expert interview)

Input	%
EPS foam tray and PVC film	5
Butcher paper	7-10

5.6 Life cycle greenhouse gas emissions

a)

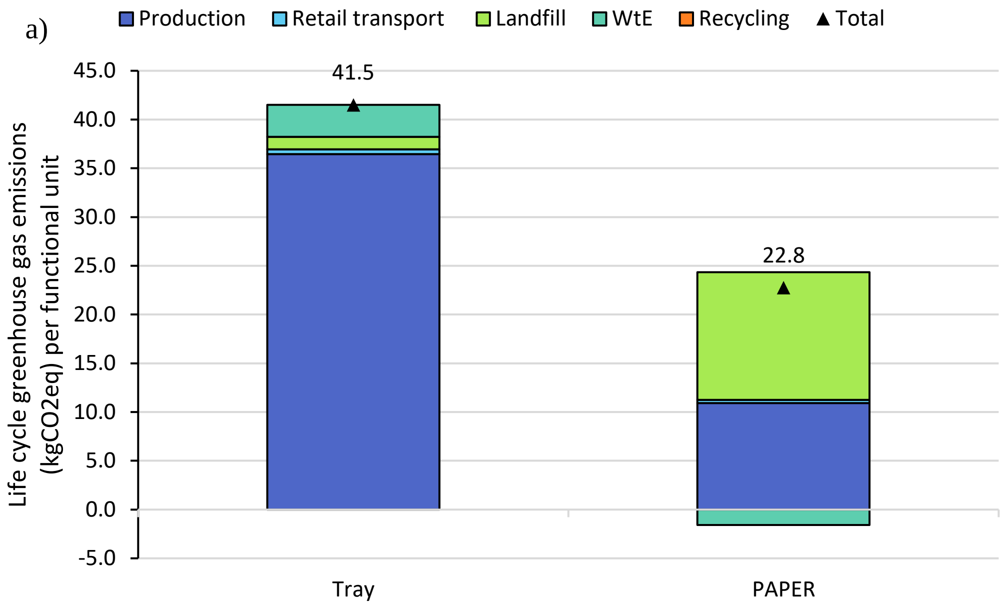

b)

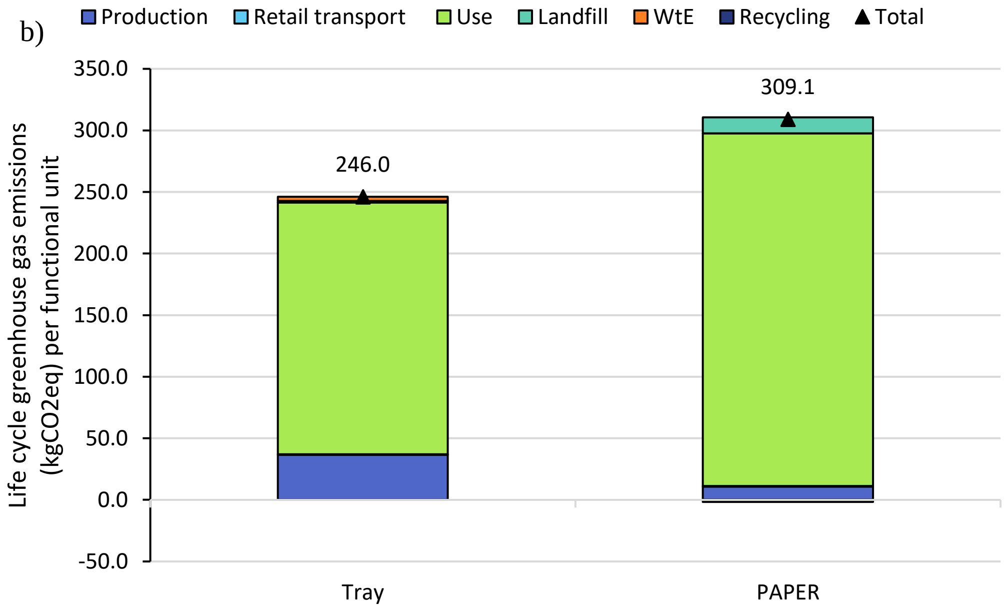

Figure S10 a) Life cycle GHG emissions excluding use phase emissions and b) life cycle GHG emissions including use phase emissions ($kgCO_{2eq}$ per 1,000 lb of pork). Climate impacts from deforestation, landfill carbon storage and paper combustion are excluded.

6 WET PET FOOD PACKAGING

6.1 Functional unit

1,000 oz of wet pet food packaged in 3 oz packaging size (e.g., Multi-layer pouch, Aluminium can, steel can)

6.2 Raw material acquisition, manufacture and transport

Table S42 Weight of one container

	Weight, g	Source	Comment
Multi-layer pouch	6	Actual measurement	
Aluminium can	10	Actual measurement	
Steel can	29		No available 3 oz steel can for pet food for direct weight measurement – assume same wall thickness as Aluminium can and approximate steel can weight using density ratio of steel to Aluminium<br>Steel density: 7.85 g/cm3 Aluminium density: 2.7 g/cm3

Table S43 Weight breakdown of multi-layer pouch (Source: expert interview)

	Share of total weight, %
PET layer	5
Aluminium layer	20
PP layer	75

Table S44 Wet pet food packaging US 2020 scenario: reference flows and EoL disposition

	Reference flow*		EoL pathway (%)			
	Quantity	Weight (kg)	Recycling	WtE	Landfill	Reuse
Flexible pouch	1 (3 oz size)	2.0	20.0	0.0	80.0	0.0
Aluminium can	1 (3 oz size)	3.3	50.0	10.0	40.0	0.0
Steel can	1 (3 oz size)	10.0	71.0	5.8	23.2	0.0

* for 1,000 oz of wet pet food

Table S45 GHG emissions [1,5]

	$kgCO_{2eq}$ per functional unit
Multi-layer pouch	7
Aluminium can	40
Steel can	40

6.3 Retail transport

Table S46 Retail transport [1]

	$kgCO_{2eq}$ per functional unit	Comment
Multi-layer pouch	0.1	Average miles per shipment is 497
Aluminium can	0.1	Average miles per shipment is 331
Steel can	0.3	Average miles per shipment is 331

6.4 End-of-life disposition

Table S47 Recycling rate and associated GHG emissions

	Recycling rate, %	$kgCO_{2eq}$ per functional unit
Multi-layer pouch	0	0
Aluminium can	50	-17
Steel can	71	-14

Table S48 Landfill rate and associated GHG emissions

	Landfill rate, %	$kgCO_{2eq}$ per functional unit	Comment
Multi-layer pouch	80	0.04	Assume US landfill vs. WtE mix of 80% vs. 20% for non-recycled material
Aluminium can	40	0.03	Assume US landfill vs. WtE mix of 80% vs. 20% for non-recycled material
Steel can	23	0.05	Assume US landfill vs. WtE mix of 80% vs. 20% for non-recycled material

Table S49 WtE rate and associated GHG emissions

	Incineration/waste-to-energy rate, %	$kgCO_{2eq}$ per functional unit	Comment
Multi-layer pouch	20	0.46	Assume US landfill vs. WtE mix of 80% vs. 20% for non-recycled material
Aluminium can	10	0.01	Assume US landfill vs. WtE mix of 80% vs. 20% for non-recycled material
Steel can	6	-0.99	Assume US landfill vs. WtE mix of 80% vs. 20% for non-recycled material

6.5 Use phase/indirect impacts

Table S50 Wastage and dented damage rates (Source: expert interview)

	%	Comment
Multi-layer pouch	0.1	Residual (cat) pet food left over after use
Aluminium can and steel can	1	

6.6 Life cycle greenhouse gas emissions

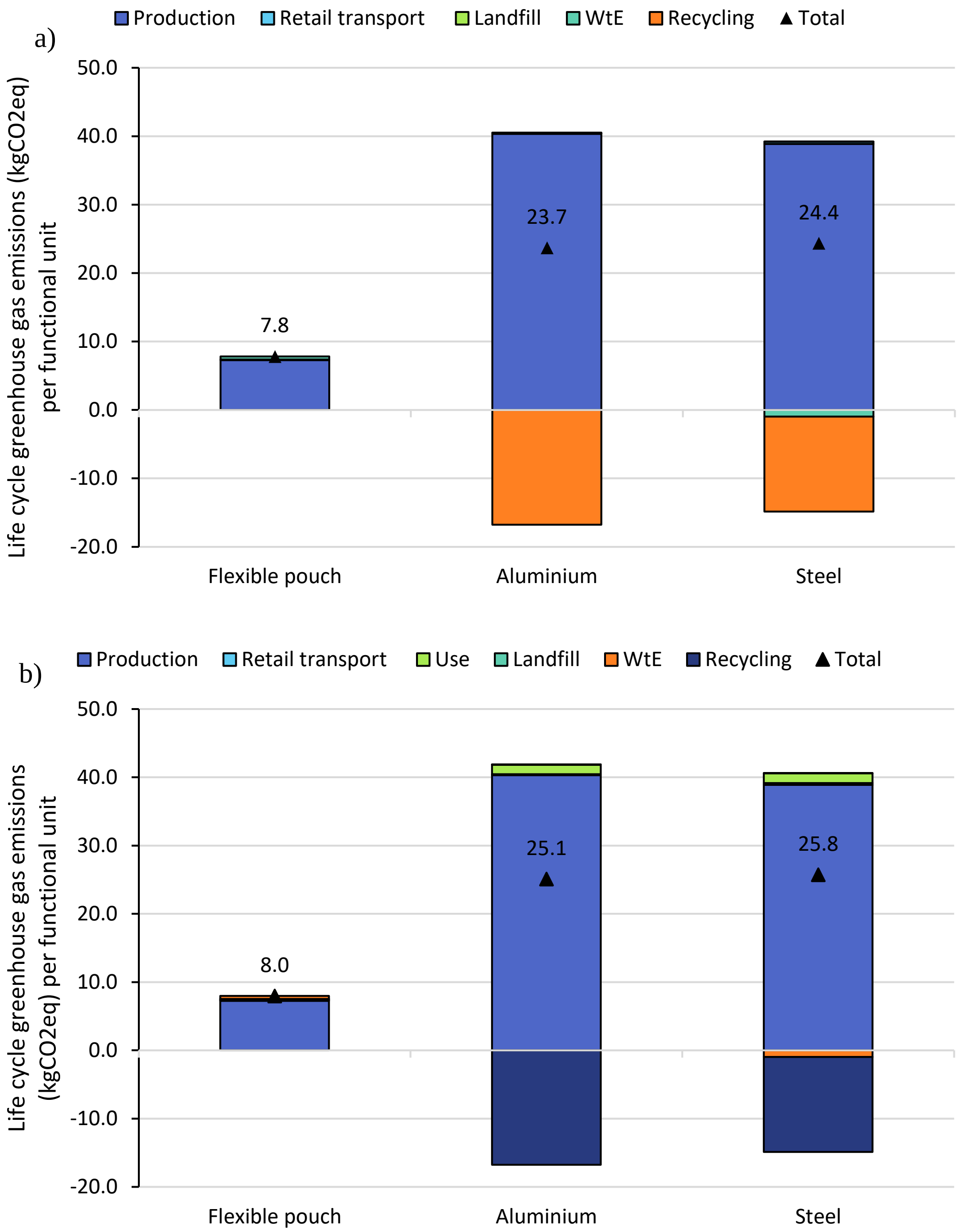

Figure S11 a) Life cycle GHG emissions excluding use phase emissions and b) life cycle GHG emissions including use phase emissions (kgCO_{2eq} per 1,000 oz of wet pet food). The production stage includes emissions from raw material acquisition, manufacture. It was assumed that 0.1% of residual food is left in multi-layer pouch after use, and 1% dented damage rate for Aluminium and steel cans.

7 INDUSTRIAL DRUMS

7.1 Functional unit

One 55-gallon drum per 10-year usage

7.2 Raw material acquisition, manufacture and transport

Table S51 Lifespan and weight of one container (Source: expert interview)

	Lifespan, years	Weight per drum, lbs
Tight head HDPE drum	5	25
Tight head steel drum	10	25

Table S52 Industrial drum US 2020 scenario: reference flows and EoL disposition

	Reference flow*		EoL pathway (%)			
	Quantity	Weight (kg)	Recycling	WtE	Landfill	Reuse
HDPE drum	2	23	20.0	16.0	64.0	0.0
Steel drum	1	16	80.0	0.0	20.0	0.0

* for 55 gal volume over 10 usage years. Maintenance is required to keep drums functional – 1% annual maintenance for HDPE drums vs. 4% for steel drums.

Table S53 GHG emissions for this life cycle stage

	$kgCO_{2eq}$ per functional unit	Source	Comment
Tight head HDPE drum	58	EPA WARM, Ecoinvent	Include conversion emissions from plastic pellets to final product
Tight head steel drum	61	EPA WARM, Ecoinvent, EUROSAC	Include conversion emissions from plastic pellets to final product

7.3 Retail transport

Table S54 GHG emissions for this life cycle stage [1]

	$kgCO_{2eq}$ per functional unit	Comment
Tight head HDPE drum	1	Average miles per shipment is 497
Tight head steel drum	0.5	Average miles per shipment is 331

7.4 End-of-life disposition

Table S55 Recycling rates and associated GHG emissions (Source: expert interview)

	Recycling rate, %	$kgCO_{2eq}$ per functional unit

Tight head HDPE drum	20	-3.8
Tight head steel drum	80	-25.6

Table S56 Landfill rates and associated GHG emissions

	Landfill rate, %	$kgCO_{2eq}$ per functional unit	Comment
Tight head HDPE drum	64	0.32	Assume US landfill vs. WtE mix of 80% vs. 20% for non-recycled material
Tight head steel drum	20	0.07	Assume US landfill vs. WtE mix of 80% vs. 20% for non-recycled material

Table S57 WtE rates and associated GHG emissions

	Incineration/waste-to-energy rate, %	$kgCO_{2eq}$ per functional unit	Comment
Tight head HDPE drum	16	5.2	Assume US landfill vs. WtE mix of 80% vs. 20% for non-recycled material
Tight head steel drum	0	0	Assume US landfill vs. WtE mix of 80% vs. 20% for non-recycled material

7.5 Use phase/indirect impacts

Table S58 Maintenance requirement (Source: expert interview)

	$kgCO_{2eq}$ per functional unit	Comment
HDPE drum	1	Repair 1 drum every year with lining for 10 years
Steel drum	4	Repair 1 drum every year with lining for 10 years

7.6 Life cycle greenhouse gas emissions

a)

b)

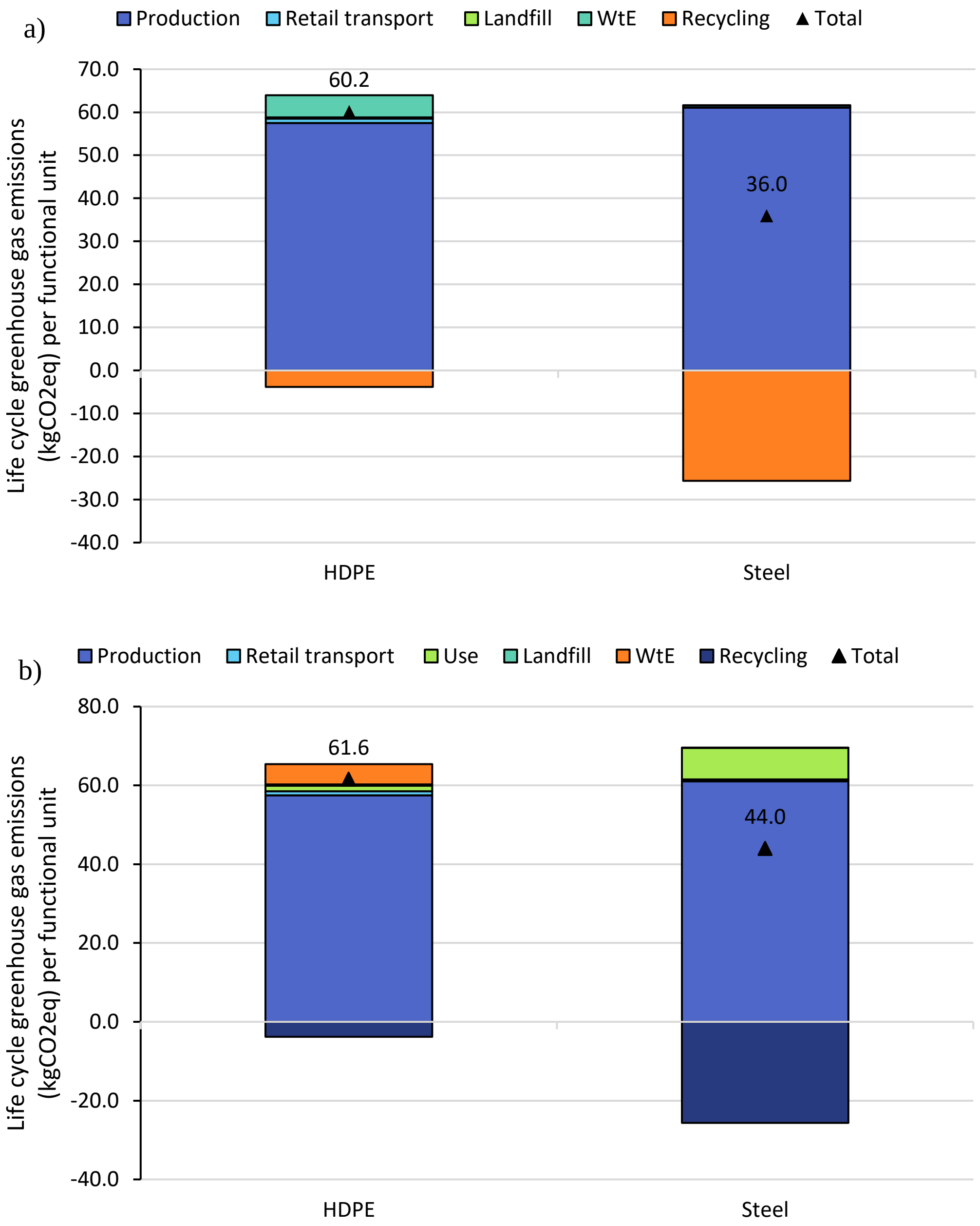

Figure S12 a) Life cycle GHG emissions excluding use phase emissions and b) life cycle GHG emissions including use phase emissions ($kgCO_{2eq}$ per drum over 10 usage years).

8 WATER CUPS

8.1 Functional unit

10,000 oz water served at cafe in 500 and 20 oz cups

8.2 Raw material acquisition, manufacture and transport

Table S59 Weight of one cup (Source: actual measurement)

	Size	Weight, g
PET cup	20 oz	19
PP cup	20 oz	14
PS foam cup	20 oz	5
Paper cup	20 oz	16
Glass cup	20 oz	400

Table S60 Water cup US 2020 scenario: reference flows and EoL disposition

	Reference flow*		EoL pathway (%)		
	Quantity	Weight (kg)	Recycling	WtE	Landfill
PET cups	500	10.0	10	18	72
Paper cups	500	8.0	0	20	80
Glass cups	1	0.4	0	20	80
EPS cups	500	2.5	0	20	80
PP cups	500	7.0	0	20	80

* Functional unit defined as 20 oz size cups, used for 10,000 oz of water

Table Ss61 GHG emissions for this life cycle stage

	$kgCO_{2eq}$ per functional unit	Source	Comment
PET cup	31	[1,5]	Include conversion emissions from plastic pellets to final product
PP cup	18	[1,5]	Include conversion emissions from plastic pellets to final product
PS foam cup	9	[1,5]	Include conversion emissions from plastic pellets to final product
Paper cup	10	[1,5]	Include LDPE lining
Glass cup	0.3	[1]	Assume 500 reuses

8.3 Retail transport

Table S62 GHG emissions for this life cycle stage [1]

	$kgCO_{2eq}$ per functional unit	Comment
PET cup	0.4	Average miles per shipment is 497
PP cup	0.3	Average miles per shipment is 497
PS foam cup	0.1	Average miles per shipment is 497
Paper cup	0.4	Average miles per shipment is 675
Glass cup	0.01	Average miles per shipment is 356

8.4 End-of-life disposition

Table S63 Recycling rates and associated GHG emissions

	Recycling rate, %	$kgCO_{2eq}$ per functional unit	Source	Comment
PET cup	10	-1.1	[9]	Mass allocation following EPA model
PP cup	0	0	Expert interview	
PS foam cup	0	0	Expert interview	
Paper cup	0	0	Expert interview	
Glass cup	0	0	Expert interview	

Table S64 Landfill rates and associated GHG emissions

	Landfill rate, %	$kgCO_{2eq}$ per functional unit	Comment
PET cup	72	0.2	Assume US landfill vs. WtE mix of 80% vs. 20% for non-recycled material
PP cup	80	0.1	Assume US landfill vs. WtE mix of 80% vs. 20% for non-recycled material
PS foam cup	80	0.04	Assume US landfill vs. WtE mix of 80% vs. 20% for non-recycled material
Paper cup	80	0.5	Assume US landfill vs. WtE mix of 80% vs. 20% for non-recycled material
Glass cup	80	0.01	Assume US landfill vs. WtE mix of 80% vs. 20% for non-recycled material

Table S65 WtE rates and associated GHG emissions

	Incineration/ waste-to-energy rate, %	$kgCO_{2eq}$ per functional unit	Comment
PET cup	18	2.3	Assume US landfill vs. WtE mix of 80% vs. 20% for non-recycled material

PP cup	20	2.0	Assume US landfill vs. WtE mix of 80% vs. 20% for non-recycled material
PS foam cup	20	0.9	Assume US landfill vs. WtE mix of 80% vs. 20% for non-recycled material
Paper cup	20	-0.7	Assume US landfill vs. WtE mix of 80% vs. 20% for non-recycled material
Glass cup	20	0.003	Assume US landfill vs. WtE mix of 80% vs. 20% for non-recycled material

8.5 Use phase/indirect impacts

Table S66 Breakage rate and GHG emissions associated with washing with hot water

	Breakage rate, %	$kgCO_{2eq}$ per functional unit	Source	Comment
Glass cups	2	29	[10,11]	Assume 50 cups per wash in a commercial dishwasher with dishwashing pod and hot-water

8.6 Life cycle greenhouse gas emissions

a)

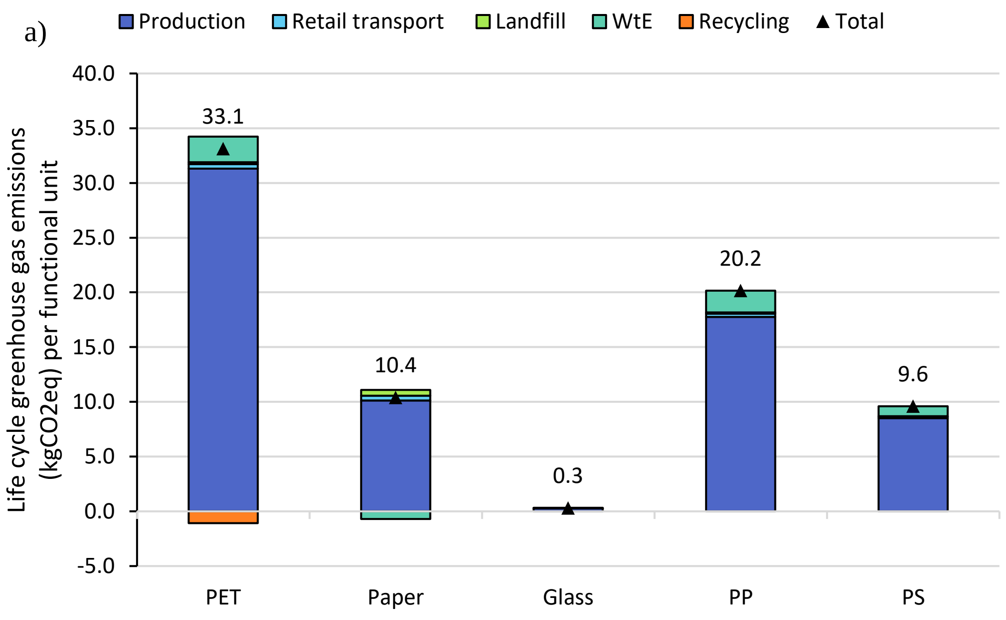

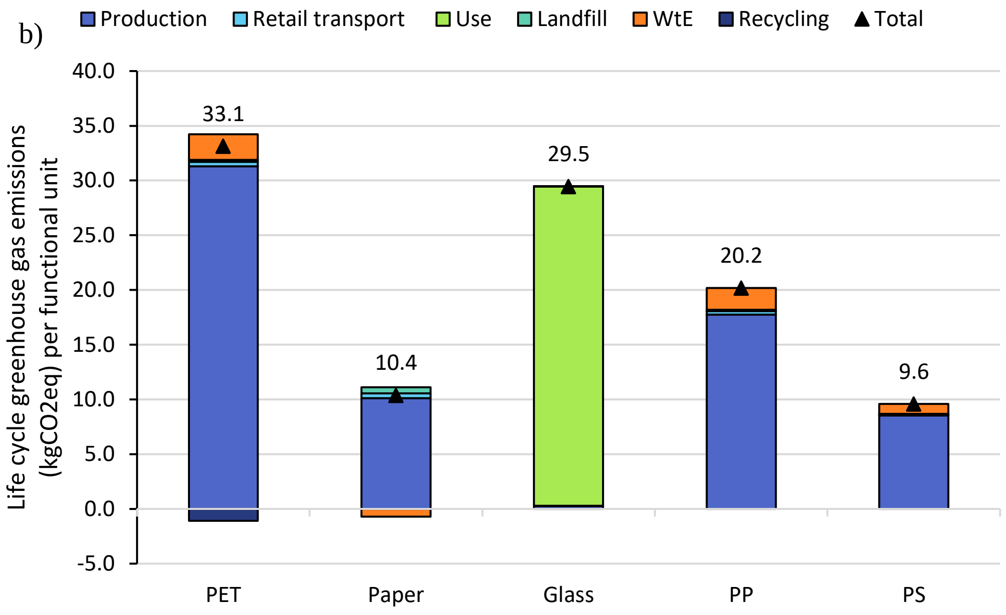

Figure S13 a) Life cycle GHG emissions excluding use phase emissions and b) life cycle GHG emissions including use phase emissions for water cups, US 2020 scenario (kgCO_{2eq} per 10,000 oz of water). Wood offers small carbon sink in landfill due to captured cellulose. GHG emissions from deforestation, landfill carbon storage and paper combustion are excluded. Negligible breakage rate for EPS, PP, PET and paper (~0%); 2% breakage rate for glass with new glass cup production as replacement.

9 HANDSOAP BOTTLES

9.1 Functional unit

One-year liquid hand soap consumption for a household of 4 (assume each person washes hand 3 times per day and each soap bottle can be reused 18 times)

9.2 Raw material acquisition, manufacture and transport

Table S67 Weight of one container (Source: actual measurement)

	Size, oz	Weight, g
HDPE bottle	12.5	50
Glass bottle	12	350

Table S68 Hand soap bottle US 2020 scenario: reference flows and EoL disposition

	Reference flow*		EoL pathway (%)		
	Quantity	Weight (kg)	Recycling	WtE	Landfill
HDPE bottles (not reused)**	18*1*12.5 oz	7.70	29	14	57
Glass bottle (reused)	1*18*12.5 oz	0.40	15	17	68
HDPE bottle (reused)	1*18*12.5 oz	0.43	29	14	57

* for 222 oz of soap.

** While the HDPE and glass bottle can be reused 18 times, in one of our alternatives 18 HDPE bottles are not reused. The functional unit is soap consumption of a household of 4 in a year, each person washes hand with soap 3 times a day. 5% wastage rate from refilling of reusable bottles (e.g. thrown away residual soap in pouch, accidental spillage of soap during transfer). Negligible breakage rate for HDPE; 2% breakage rate for glass, accounting for additional production of glass bottles.

Table S69 GHG emissions for this life cycle stage [1,5]

	$kgCO_{2eq}$ per functional unit	Comment
HDPE bottle (disposable; not reused)	2.28	Include conversion emissions from plastic pellets to final product
Glass bottle (reused 18 times)	0.50	
HDPE bottle (reused 18 times)	0.13	Include conversion emissions from plastic pellets to final product

9.3 Retail transport

Table S70 GHG emissions for this life cycle stage [1]

	$kgCO_{2eq}$ per functional unit	Comment
HDPE bottle (disposable; not reused)	0.3	Average miles per shipment is 497
Glass bottle (reused 18 times)	0.01	Average miles per shipment is 356
HDPE bottle (reused 18 times)	0.02	Average miles per shipment is 497

9.4 End-of-life disposition

Table S71 Recycling rates and associated GHG emissions [2]

	Recycling rate, %	$kgCO_{2eq}$ per functional unit	Comment
HDPE bottle (disposable; not reused)	29	-0.2	Mass allocation ratios from EPA model
Glass bottle (reused 18 times)	15	-0.04	Mass allocation ratios from EPA model
HDPE bottle (reused 18 times)	29	-0.01	Mass allocation ratios from EPA model

Table S72 Landfill rates and associated GHG emissions

	Landfill rate, %	$kgCO_{2eq}$ per functional unit	Comment
HDPE bottle (disposable; not reused)	57	0.012	Assume US landfill vs. WtE mix of 80% vs. 20% for non-recycled material
Glass bottle (reused 18 times)	68	0.007	Assume US landfill vs. WtE mix of 80% vs. 20% for non-recycled material
HDPE bottle (reused 18 times)	57	0.001	Assume US landfill vs. WtE mix of 80% vs. 20% for non-recycled material

Table S73 WtE rates and associated GHG emissions

	Incineration/waste-to-energy rate, %	$kgCO_{2eq}$ per functional unit	Comment
HDPE bottle (disposable; not reused)	14	0.18	Assume US landfill vs. WtE mix of 80% vs. 20% for non-recycled material
Glass bottle (reused 18 times)	17	-0.03	Assume US landfill vs. WtE mix of 80% vs. 20% for non-recycled material
HDPE bottle (reused 18 times)	14	0.01	Assume US landfill vs. WtE mix of 80% vs. 20% for non-recycled material

9.5 Use phase/indirect impacts

Table S74 Breakage and wastage rate

	%	Source	Comment
Glass bottle (reused 18 times)	2	Industry report, Expert interview	
Glass bottle (reused 18 times) and HDPE bottle (reused 18 times)	5	Expert interview	Wastage from refilling of reusable bottles (e.g., thrown away residual soap in pouch, accidental spillage of soap during transfer)

Table S75 Soap wastage from refilling and associated GHG emissions [1,12]

	$kgCO_{2eq}$ per functional unit	Comment
Glass bottle (reused 18 times)	1.00	
HDPE bottle (reused 18 times)	0.94	Exclude first HDPE bottle for consistency with glass bottle comparison

9.6 Life cycle greenhouse gas emissions

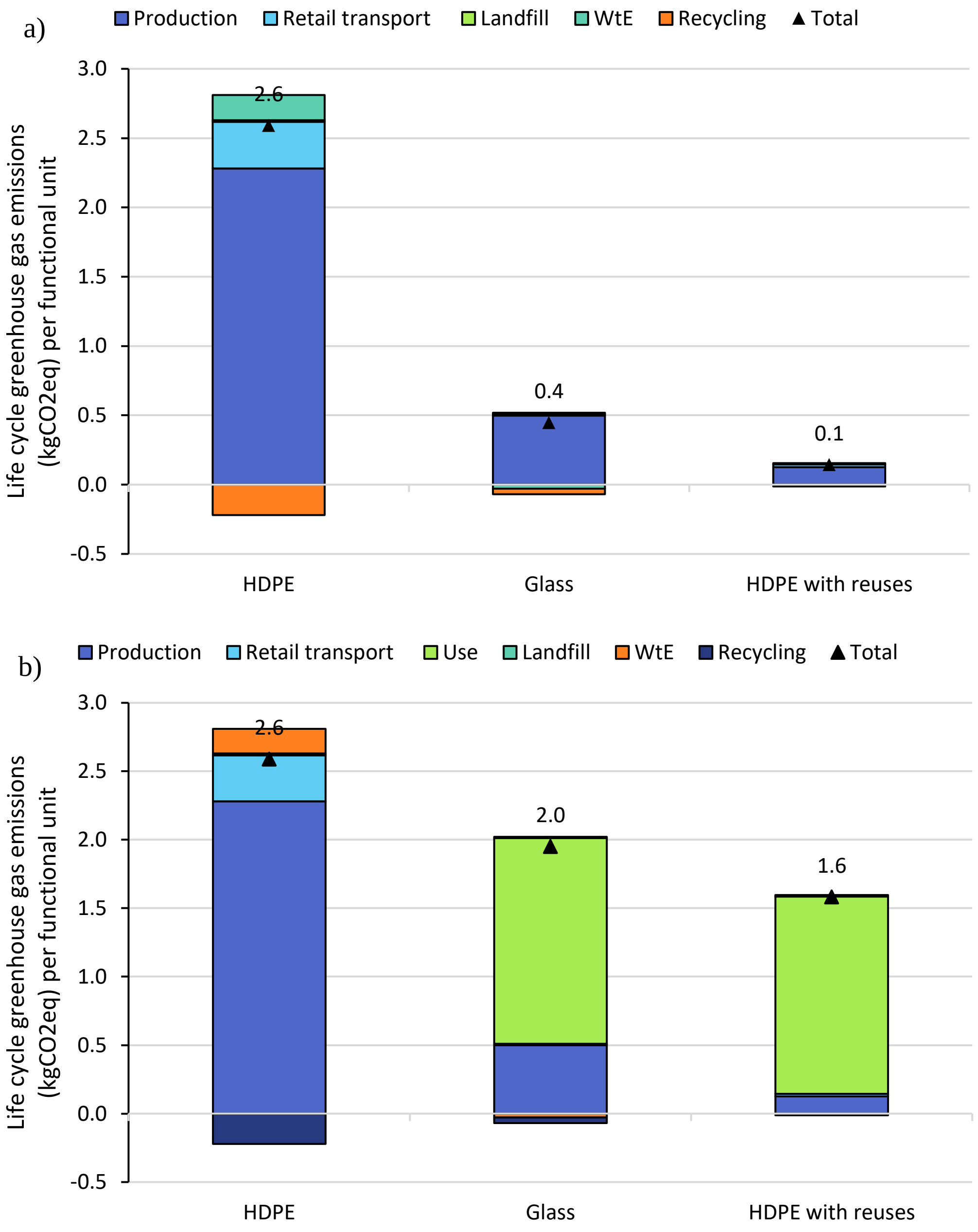

Figure S14 a) Life cycle GHG emissions excluding use phase emissions and b) life cycle GHG emissions including use phase emissions ($kgCO_{2eq}$ per 222 oz of soap).

10 MUNICIPAL SEWER PIPES

10.1 Functional unit

100 feet of 12-inch gravity sewer main pipes over a service life of 100 years

10.2 Raw material acquisition, manufacture and transport

Table S76 Weight of functional unit

	Specification	Weight per foot (lbs/ft)	Lifespan, years	Source
PVC pipe	ASTM D3034 or ASTM D2241	18.9	100	Product website
Reinforced concrete pipe	ASTM C76 Wall B	127	100	American Concrete Pipe Association
Ductile iron pipe	ASTM A746	40	100	Product website

Table S77 Municipal sewer pipe – gravity and force main US 2020 scenario: reference flows and EoL disposition

	Reference flow*		EoL pathway (%)		
	Quantity	Weight (kg)	Recycling	WtE	Landfill
PVC pipe (15”)	100 ft	860	9	15	76
Reinforced concrete pipe (15”)	100 ft	5,570	0	0	100
PVC pipe (12”)	100 ft	720	9	15	76
Ductile iron pipe (12”)	100 ft	1,820	30	0	70

* for usage of 100 years.

Table S78 GHG emissions for this life cycle stage

	$kgCO_{2eq}$ per functional unit	Source
PVC pipe	2,270	EPA WARM, Ecoinvent
Reinforced concrete pipe	4,173	Ecoinvent
Ductile iron pipe	3,977	McKinsey MetalSpans, Energy Saving using 7Epsilon, Arjunwadkar et al, 2015, Estimate of energy consumption and CO2 emission associated with the production, use and final disposal of PVC, HDPE, PP, ductile iron and concrete pipes, Recio et al., 2015

10.3 Retail transport

Table S79 GHG emissions for this life cycle stage [13]

	$kgCO_{2eq}$ per functional unit	Comment
PVC pipe	31	Assume same transport distance and hence same EF
Reinforced concrete pipe	206	Assume same transport distance and hence same EF
Ductile iron pipe	65	Assume same transport distance and hence same EF

10.4 End-of-life disposition

Table S80 Recycling rates and associated GHG emissions

	Recycling rate, %	$kgCO_{2eq}$ per functional unit	Source
PVC pipe	9	-64	McKinsey Chemical Insights Circular model
Reinforced concrete pipe	0	0	n/a
Ductile iron pipe	30%	-971	Expert interview (Former CEO of a pipe supplier company; currently sitting on three different sewage pipe boards)

Table S81 Landfill rates and associated GHG emissions

	Landfill rate, %	$kgCO_{2eq}$ per functional unit	Source	Comment
PVC pipe	76	14	McKinsey Chemical Insights Circular model	
Reinforced concrete pipe	100	127		Assume all pipes are landfilled
Ductile iron pipe	70	28	Expert interviews	

Table S82 WtE rates and associated GHG emissions

	Incineration/waste-to-energy rate, %	$kgCO_{2eq}$ per functional unit	Source
PVC pipe	15	94	McKinsey Chemical Insights Circular model
Reinforced concrete pipe	0	0	
Ductile iron pipe	0	0	

Table S83 Advanced recycling rates and associated GHG emissions

	$kgCO_{2eq}$ per functional unit	Source
PVC pipe	0	McKinsey Chemical Insights Circular model
Reinforced concrete pipe	0	
Ductile iron pipe	0	

10.5 Use phase/indirect impacts

No measurable indirect impact – same service life of 50 years due to corrosive nature of sewage.

10.6 Life cycle greenhouse gas emissions

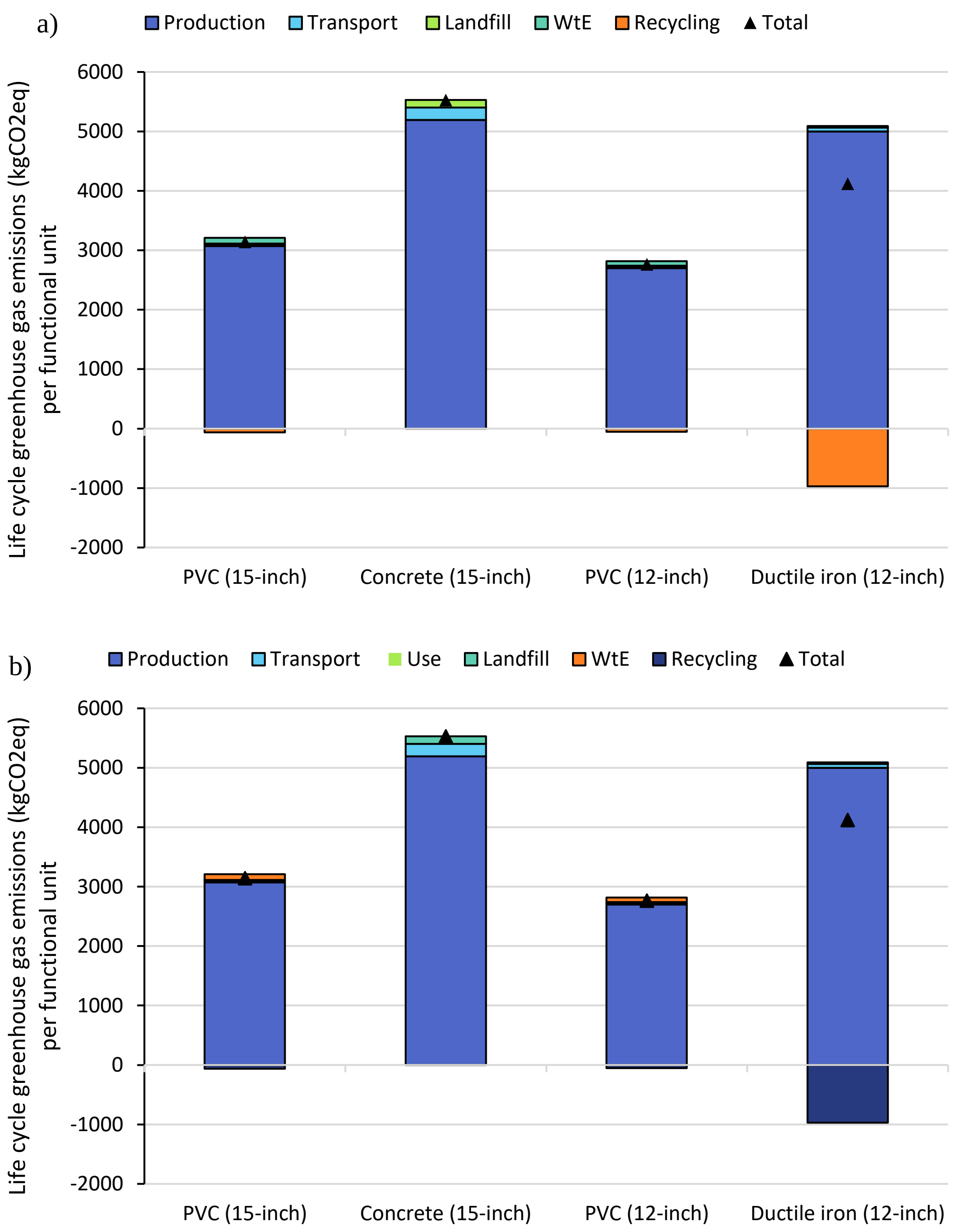

Figure S15 a) Life cycle GHG emissions excluding use phase emissions and b) life cycle GHG emissions including use phase emissions ($kgCO_{2eq}$ per 100 ft of pipe). Installation process includes use of excavator, skid steer, disinfection and bedding.

11 RESIDENTIAL WATER PIPES

11.1 Functional unit

Hot and cold-water distribution pipe system for a 2,811 square foot house (excluding watermain pipes and main wastewater line), with usage modelled in the clustered use phase as described in the source LCA (peaks of usage in morning and night for family of 4); lifetime of 50 years for pipes

11.2 Raw material acquisition, manufacture and transport

Table S84 Weight of functional unit [14]

	Weight, kg
PEX pipes with polysulfone and brass fitting	18
Copper pipes	77

Table S85 Residential water pipe US 2020 scenario: reference flows and EoL disposition

	Reference flow*		EoL pathway (%)		
	Quantity	Weight (kg)	Recycling	WtE	Landfill
PEX pipe with polysulfone and brass fittings	431 ft	19	0	0	100
Copper type L pipes	431 ft	77	30	0	70

* per 2,811 ft^2 home for usage of 50 years

Table S86 GHG emissions for this life cycle stage [1,14]

	$kgCO_{2eq}$ per functional unit
PEX pipes with polysulfone and brass fitting	78
Copper pipes	209

11.3 Retail transport

Table S87 GHG emissions for this life cycle stage [1,14]

	$kgCO_{2eq}$ per functional unit
PEX pipes with polysulfone and brass fitting	2
Copper pipes	6

11.4 End-of-life disposition

Table S88 Recycling rates and associated GHG emissions (Source: expert interview)

	Recycling rate, %	$kgCO_{2eq}$ per functional unit
PEX pipes with polysulfone and brass fitting	0	0
Copper pipes	21	-24.4

Table S89 Landfill rates and associated GHG emissions (Source: expert interview)

	Landfill rate, %	$kgCO_{2eq}$ per functional unit
PEX pipes with polysulfone and brass fitting	100	0.4
Copper pipes	79	1.34

Table S90 WtE rates and associated GHG emissions (Source: expert interview)

	Incineration/waste- to-energy rate, %	$kgCO_{2eq}$ per functional unit
PEX pipes with polysulfone and brass fitting	0	0
Copper pipes	0	0

11.5 Use phase/indirect impacts

Table S91 Heat loss from water pipes and associated GHG emissions [1,14]

	$kgCO_{2eq}$ per functional unit
PEX pipe with polysulfone and brass fittings	82,602
Copper type L pipes	112,716

11.6 Life cycle greenhouse gas emissions

a)

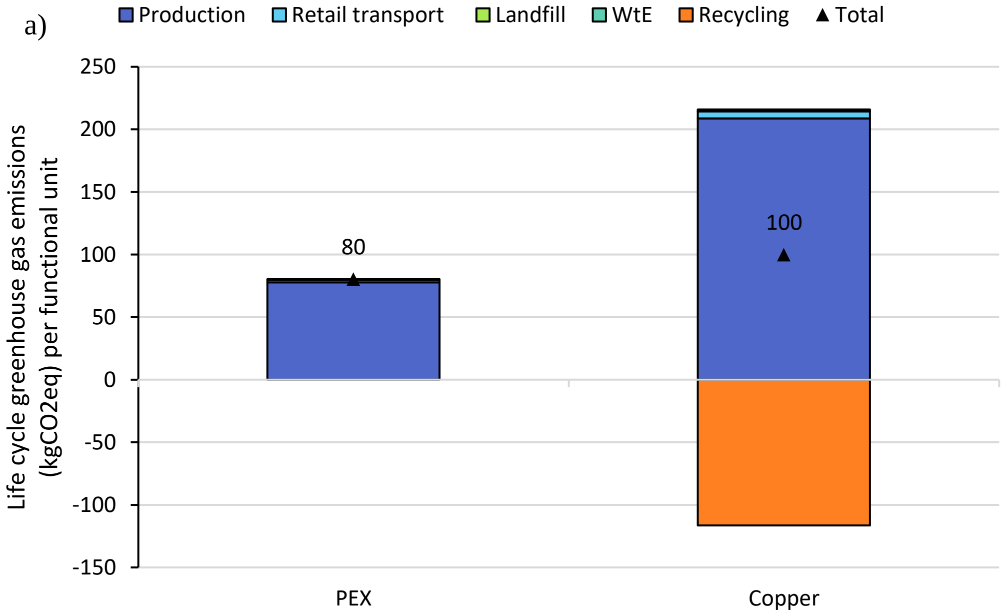

b)

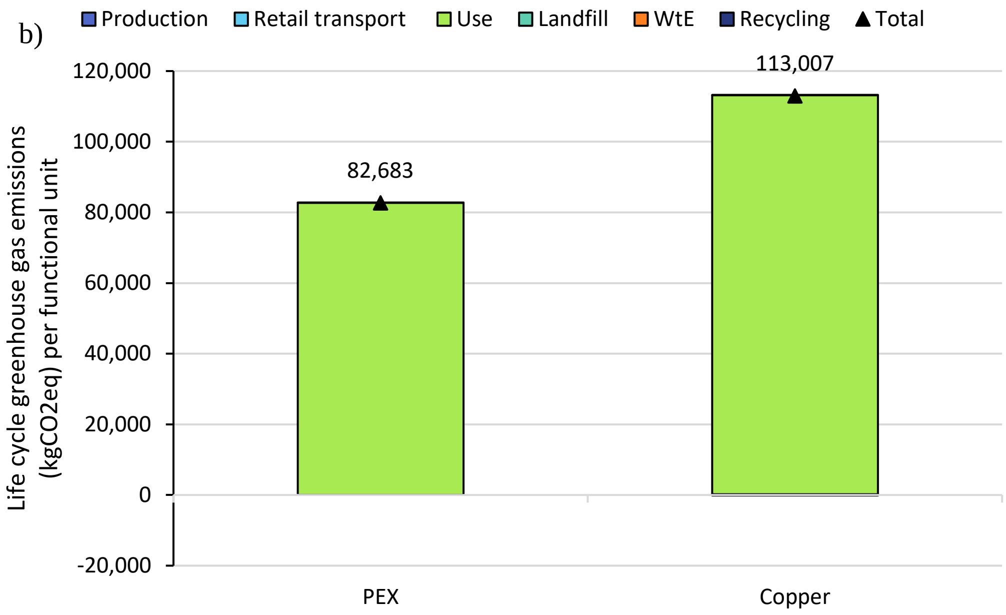

Figure S16 a) Life cycle GHG emissions excluding use phase emissions and b) life cycle GHG emissions including use phase emissions ($kgCO_{2eq}$ per 2,811 ft^2 home). Emissions from incremental heat loss in pipes based on clustered use phase (i.e., most water use concentrated in mornings and evenings for a family of 4).

12 BUILDING INSULATION

12.1 Functional unit/use phase scenario

Insulation required in 2x6 inches external walls (5.5 inches cavity) on a c in Richmond, VA; insulated in compliance with regional standards: attic (R=49) and walls (R=20); gas-fired air furnace and AC, and gas-fired water heater; insulation service life of 75 years

12.2 Raw material acquisition, manufacture and transport

Table S92 Weight of functional unit

	Total wall insulation functional units	Weight, kg	Source
Spray polyurethane foam (SPF), open cell	822	330	SPF Residential Energy Modelling Analysis 2021
Fibreglass batt	822	360	National Institute of Standards and Technology

Table S93 Insulation US 2020 scenario: reference flows and EoL disposition

	Reference flow*		EoL pathway (%)		
	Quantity	Weight (kg)	Recycling	WtE	Landfill
SPF	~5.5 inches, R=20	330	0	0	100
Fibreglass batt	~5.5 inches, R=20	360	0	0	100

* Functional unit defined as insulation required on 2x6in external walls (5.5in cavity) on a 2,512 square-foot two-story wood framed single-family detached home in Richmond, VA (SPFA, 2021), insulated in compliance with regional standards: Attic (R-49) and walls (R-20), gas-fired air furnace and AC, and gas-fired water heater.

Table S94 GHG emissions for this life cycle stage [15]

	$kgCO_{2eq}$ per functional unit
Spray polyurethane foam (SPF), open cell	1.6
Fibreglass batt	1.2

12.3 Retail transport

Table S95 GHG emissions for this life cycle stage [1]

	$kgCO_{2eq}$ per functional unit	Comment
Spray polyurethane foam (SPF), open cell	0.01	Average miles per shipment is 356
Fibreglass batt	0.01	Average miles per shipment is 356,

12.4 End-of-life disposition

Table S96 Recycling rates and associated GHG emissions (Source: expert interview)

	Recycling rate, %	$kgCO_{2eq}$ per functional unit
Spray polyurethane foam (SPF), open cell	0	0
Fibreglass batt	0	0

Table S97 Landfill rates and associated GHG emissions (Source: expert interview)

	Landfill rate, %	$kgCO_{2eq}$ per functional unit
Spray polyurethane foam (SPF), open cell	100	0.01
Fibreglass batt	100	0.01

Table S98 WtE rates and associated GHG emissions (Source: expert interview)

	Incineration/waste-to-energy rate, %	$kgCO_{2eq}$ per functional unit
Spray polyurethane foam (SPF), open cell	0	0
Fibreglass batt	0	0

12.5 Use phase/indirect impacts

Table S99 GHG emissions during the use phase

	$kgCO_{2eq}$ per functional unit	Comment
Spray polyurethane foam (SPF), open cell	7.7	Building insulation service life assumed to be 75 years according to the North American PCR (Product Category Rules)

12.6 Life cycle greenhouse gas emissions

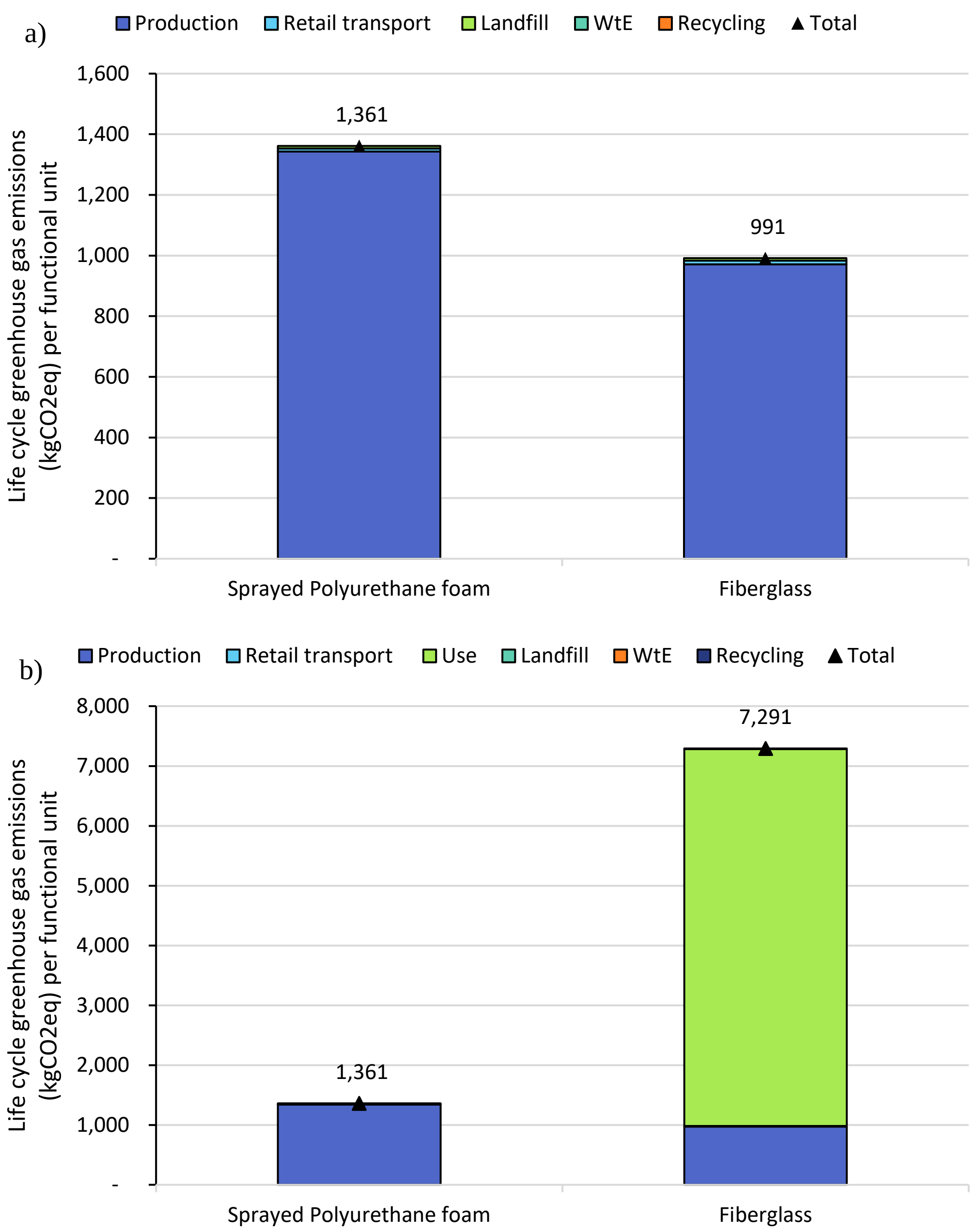

Figure S17 a) Life cycle GHG emissions excluding use phase emissions and b) life cycle GHG emissions including use phase emissions for building insulation, US 2020 scenario (kgCO_{2eq} per 2,512 ft^2 home). The production stage includes emissions from raw material acquisition and manufacture. Spray polyurethane foam has the lowest overall GHG impact despite higher production emissions due to its air impermeability which saves heating and cooling energy, and even when installed to the same R-value as fibreglass insulation.

13 FURNITURE SET

13.1 Functional unit

Furniture set of one square table and four chairs, with lifespan of 10 years; models used for each material are selected from real life examples

13.2 Raw material acquisition, manufacture and transport

Table S100 Weight of functional unit

	Weight, lb	Lifespan	Source
PP furniture	45	10	Manufacturer website, Expert interview
Steel furniture	88	10	Manufacturer website, Expert interview
Wood furniture	85	10	Manufacturer website, Expert interview

Table S101 Furniture US 2020 scenario: reference flows and EoL disposition

	Reference flow*		EoL pathway (%)		
	Quantity	Weight (kg)	Recycling	WtE	Landfill
PP table and chairs	1 set	20	0	0	100
Steel table and chairs	1 set	40	10	0	90
Wood table and chairs	1 set	39	0	0	100

* Functional unit defined as a set of square table and four chairs, used over 10-year lifespan Table S102 GHG emissions from this life cycle stage [1]

	$kgCO_{2eq}$ per functional unit
PP furniture	52
Steel furniture	13
Wood furniture	79

13.3 Retail transport

Table S103 GHG emissions from this life cycle stage [1]

	$kgCO_{2eq}$ per functional unit	Comment
PP furniture	0.9	Average miles per shipment is 497
Steel furniture	1.32	Average miles per shipment is 331
Wood furniture	0.02	Average miles per shipment is 246

13.4 End-of-life disposition

Table S104 Recycling rates and associated GHG emissions (Source: expert interview)

	Recycling rate, %	$kgCO_{2eq}$ per functional unit
PP furniture	0	0
Steel furniture	10	-8
Wood furniture	0	0

Table S105 Landfill rates and associated GHG emissions

	Landfill rate, %	$kgCO_{2eq}$ per functional unit	Comment
PP furniture	100	0.45	Assume all non-recycled furniture is landfilled
Steel furniture	90	0.79	Assume all non-recycled furniture is landfilled
Wood furniture	100	-1.01	Assume all non-recycled furniture is landfilled

Table S106 WtE rates and associated GHG emissions

	Incineration/waste-to-energy rate, %	$kgCO_{2eq}$ per functional unit
PP furniture	0	0
Steel furniture	0	0
Wood furniture	0	0

13.5 Use phase/indirect impacts

No measurable indirect impact.

13.6 Life cycle greenhouse gas emissions

a)

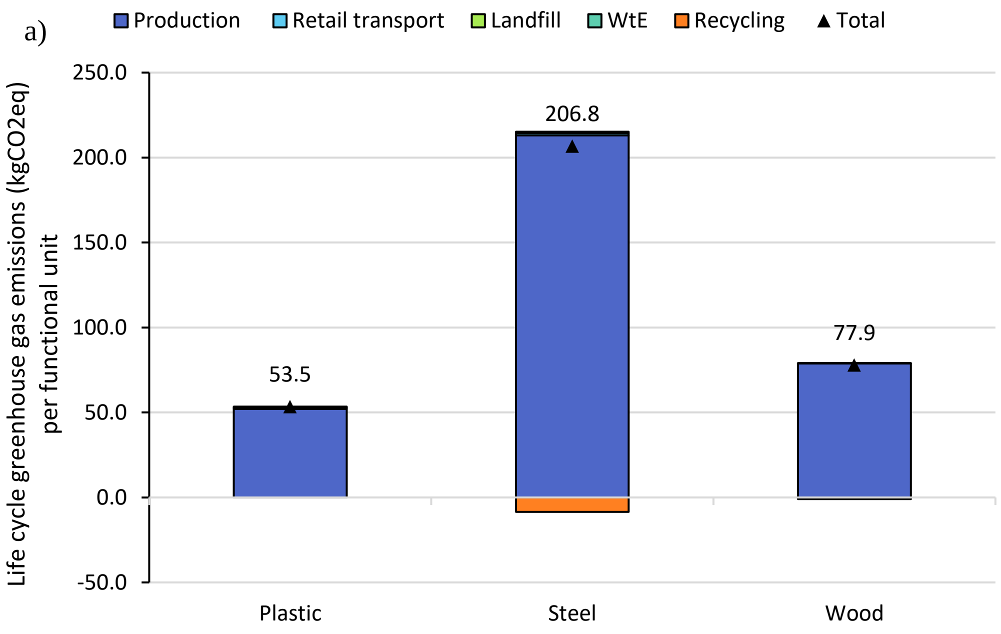

b)

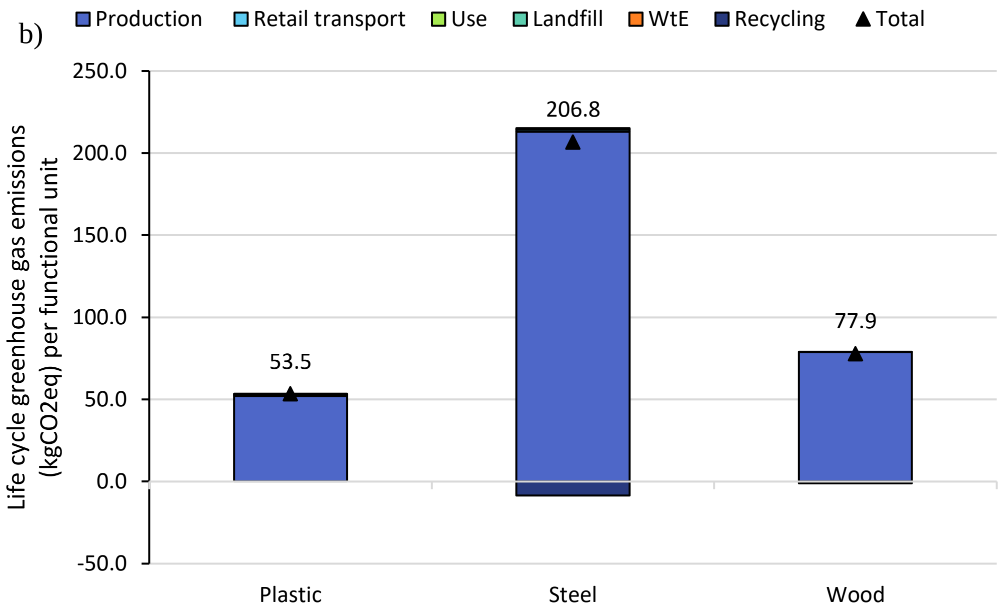

Figure S18 a) Life cycle GHG emissions excluding use phase emissions and b) life cycle GHG emissions including use phase emissions for outdoor furniture, US 2020 scenario (kgCO_{2eq} per set of table and four chairs). Wood offers small carbon sink in landfill due to captured cellulose.

14 AUTOMOTIVE FUEL TANKS

14.1 Functional unit

One fuel tank for a 4-seat hybrid sedan with a lifespan of 200,000 miles; assume no difference in weight of vehicle in operation other than fuel tank material.

We select battery pack top enclosures as a representative application in BEVs. The two most common material types are steel and a composite material comprised of PP and fiberglass reinforced PP. As with hybrid vehicle fuel tank application, the weight of battery packs is a consideration in the use phase. BEVs are also more energy efficient than hybrid vehicles, and energy requirements can be met with lower-carbon energy; the share of renewables or nuclear energy in the United States is significant, at around 40% of the total power mix.

14.2 Raw material acquisition, manufacture and transport

Table S107 Weight of functional unit

	Vehicle model	Weight, kg	Source
HDPE gas tank	Honda Accord Hybrid	6.5	A2Mac1
Steel gas tank	Kia Optima Hybrid	11.5	A2Mac1

Table S108 HEV fuel tank US 2020 scenario: reference flows and EoL disposition

	Reference flow*		EoL pathway (%)		
	Quantity	Weight (kg)	Recycling	WtE	Landfill
HDPE fuel tank	1	6.5	65	0	35
Steel fuel tank	1	11.5	95	0	5

* Functional unit defined as one battery pack top-enclosure for a compact BEV SUV, driven 200,000 miles. It was assumed recycling process loss rates of 23% and 2% for HDPE and steel, respectively.

Table S109 GHG emissions for this life cycle stage [1,5]

	$kgCO_{2eq}$ per functional unit
HDPE gas tank	25
Steel gas tank	36

14.3 Retail transport

Table S110 GHG emissions for this life cycle stage [1]

	$kgCO_{2eq}$ per functional unit	Comment
HDPE gas tank	0.2	Average miles per shipment is 497
Steel gas tank	0.4	Average miles per shipment is 331

14.4 End-of-life disposition

Table S111 Recycling rates and associated GHG emissions (Source: expert interview)

	Recycling rate, %	$kgCO_{2eq}$ per functional unit
HDPE gas tank	52	-3.3
Steel gas tank	93	-19.7

Table S112 Landfill rates and associated GHG emissions (Source: expert interview)

	Landfill rate, %	$kgCO_{2eq}$ per functional unit
HDPE gas tank	48	0.08
Steel gas tank	7	0.02

Table S113 WtE rates and associated GHG emissions (Source: expert interview)

	Incineration/waste-to-energy rate, %	$kgCO_{2eq}$ per functional unit
HDPE gas tank	0	0
Steel gas tank	0	0

14.5 Use phase/indirect impacts

Table S114 Fuel efficiency improvement

	gal/mile/kg	Source	Comment
HDPE gas tank, steel gas tank	0.000015	[16]	Every 100 kg weight reduction will achieve a reduction of<br>0.69 L/100 km in fuel consumption<br>Hybrid vehicles have on average two times better gas mileage than ICE vehicles

14.6 Life cycle greenhouse gas emissions

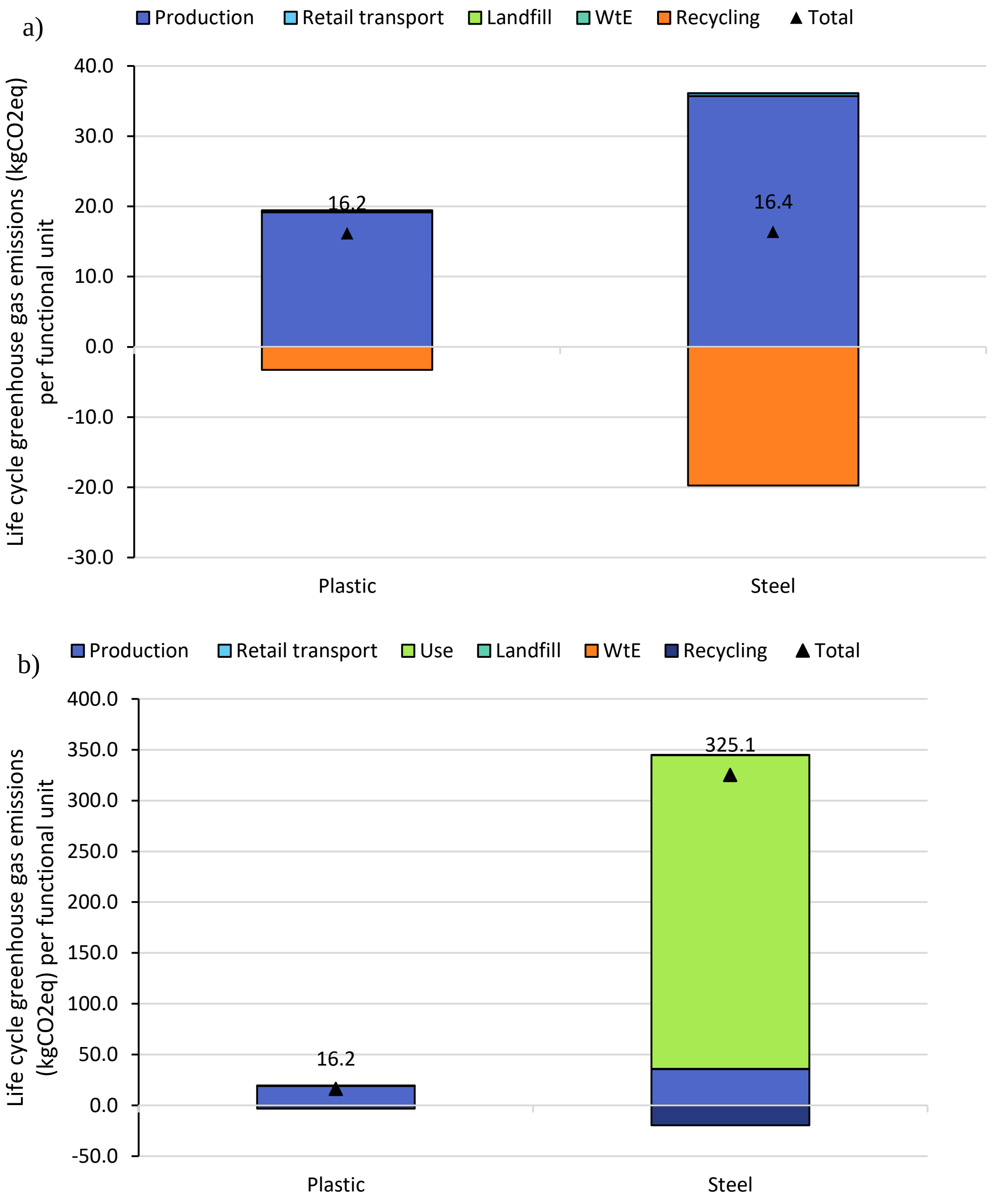

Figure S19 a) Life cycle GHG emissions excluding use phase emissions and b) life cycle GHG emissions including use phase emissions for HEV fuel tank, US 2020 scenario ($kgCO_{2eq}$ per gas tank).

15 AUTOMOTIVE ELECTRIC-VEHICLE BATTERY PACK TOP ENCLOSURES

15.1 Functional unit

One battery pack top enclosure for a compact BEV SUV with a lifespan of 200,000 miles; assume no difference in weight of vehicle in operation other than battery top enclosure

15.2 Raw material acquisition, manufacture and transport

Table S115 Weight of one function unit

	Vehicle model	Weight, kg	Source
Glass fibre reinforced PP enclosure	Ford Mach-E	15.7	A2Mac1
Steel enclosure	Volkswagen ID.4	21.8	A2Mac1

Table S116 Material breakdown for glass fibre reinforced PP

	Proportion of total weight, %
PP	75
Glass fibre	25

Table S117 Battery pack enclosure US 2020 scenario: reference flows and EoL disposition

	Reference flow*		EoL pathway (%)		
	Quantity	Weight (kg)	Recycling	WtE	Landfill
PP/glass fibre enclosure	1	15.7	0	0	100
Steel enclosure	1	21.8	95	0	5

* Functional unit defined as one battery pack top-enclosure for a compact BEV SUV, driven 200,000 miles

Table S118 GHG emissions for this life cycle stage [1,5]

	$kgCO_{2eq}$ per functional unit
Glass fibre reinforced PP enclosure	36
Steel enclosure	54

15.3 Retail transport

Table S119 GHG emissions for this life cycle stage [1]

	$kgCO_{2eq}$ per functional unit	Comment
Glass fibre reinforced PP enclosure	0.5	Average miles per shipment is 497 for plastics and 356 for glass
Steel enclosure	0.7	Average miles per shipment is 331

15.4 End-of-life disposition

Table S120 Recycling rates and associated GHG emissions (Source: expert interview)

	Recycling rate, %	$kgCO_{2eq}$ per functional unit
Glass fibre reinforced PP enclosure	0	0
Steel enclosure	95	-31.9

Table S121 Landfill rates and associated GHG emissions (Source: expert interview)

	Landfill rate, %	$kgCO_{2eq}$ per functional unit
Glass fibre reinforced PP enclosure	100	0.35
Steel enclosure	5	0.03

Table S122 WtE rates and associated GHG emissions (Source: expert interview)

	Incineration/waste-to- energy rate, %	$kgCO_{2eq}$ per functional unit
Glass fibre reinforced PP enclosure	0	0
Steel enclosure	0	0

15.5 Use phase/indirect impacts

Table S123 Fuel efficiency improvement from light-weighting [17] (Source: EV database, Expert interview)

	kWh/mile/kg	Comment
Glass fibre reinforced PP enclosure; steel enclosure	0.000048	Average efficiency = 0.157 kWh/km<br>Average BEV weight = 2,100 kg<br>Rolling resistance = 40% of total resistance<br>Efficiency improvement = 0.157 / 2,100 * 40% = 0.00003 kWh/km/kg = 0.000048 kWh/mile/kg

15.6 Life cycle greenhouse gas emissions

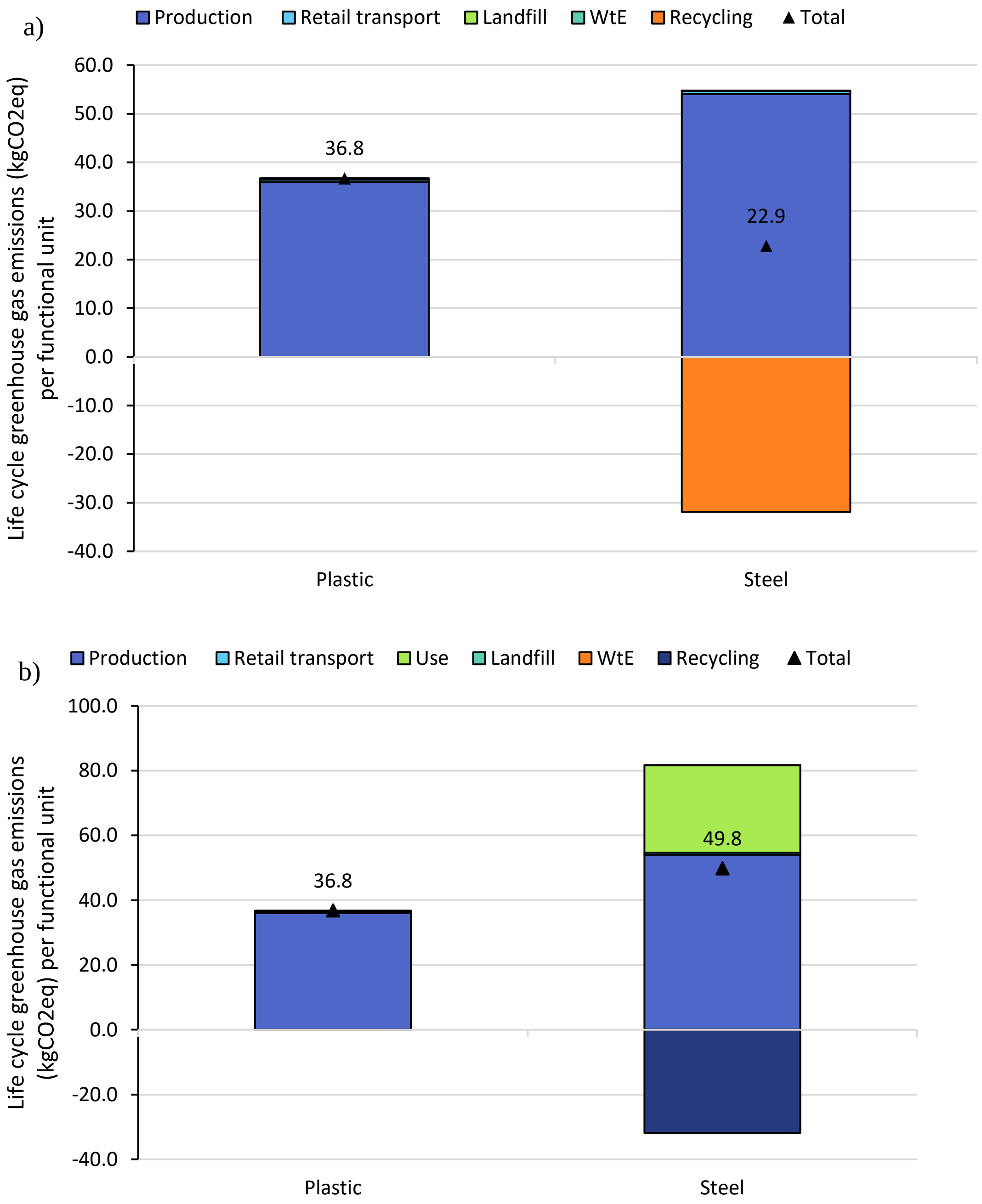

Figure S20 a) Life cycle GHG emissions excluding use phase emissions and b) life cycle GHG emissions including use phase emissions for battery pack enclosure, US 2020 scenario (kgCO_{2eq} per enclosure).

16 T-SHIRTS

16.1 Functional unit

1000 kg of fibres, regardless of discrete numbers of shirts (because volume of fibre and thus raw material varies significantly with variables like t-shirt size and brand)

Table S124 T-shirt, US 2020 scenario: reference flows and EoL disposition

	Reference flow*		EoL pathway (%)		
	Quantity	Weight (kg)	Recycling	WtE	Landfill
PET t-shirt	4,651	1,000	0	46	54
Cotton t-shirt	4,651	1,000	0	46	54

* Functional unit defined as 1,000 kg of t-shirts

16.2 Raw material acquisition, manufacture and transport

Table S125 GHG emissions for this life cycle stage

	$kgCO_{2eq}$ per functional unit	Source
Polyester t-shirt	9,488	[1,5,18,19]
Cotton t-shirt	11,423	[1,5,19]

16.3 Retail transport

Table S126 GHG emissions for this life cycle stage [1]

	$kgCO_{2eq}$ per functional unit	Comment
Polyester t-shirt	22	Average miles per shipment is 497
Cotton t-shirt	22	Assume same as polyester t-shirt

16.4 End-of-life disposition

Table S127 Recycling rates and associated GHG emissions (Source: Expert interview)

	Recycling rate, %	$kgCO_{2eq}$ per functional unit
Polyester t-shirt	0	0
Cotton t-shirt	0	0

Table S128 Landfill rates and associated GHG emissions [19]

	Landfill rate, %	$kgCO_{2eq}$ per functional unit	Comment
Polyester t-shirt	54	12.0	Assume similar end-of-life share as cotton t- shirts
Cotton t-shirt	54	879.0	Landfill vs. WtE mix based on end-of-life mix in Cotton Industry Association report

Table S129 WtE rates and associated GHG emissions [19]

	Incineration/waste-to-energy rate, %	$kgCO_{2eq}$ per functional unit	Comment
Polyester t-shirt	46	623.4	Assume similar end-of-life share as cotton t-shirts
Cotton t-shirt	46	740.0	Landfill vs. WtE mix based on end-of-life mix in Cotton Industry Association report

16.5 Use phase/indirect impacts

Table S130 GHG emissions from use of t-shirt (average washing pattern) [19]

	$kgCO_{2eq}$ per functional unit	Comment
Polyester t-shirt	6,760	Assume same usage and wash patterns for cotton and polyester t-shirts
Cotton t-shirt	6,760	Assume same usage and wash patterns for cotton and polyester t-shirts

16.6 Life cycle greenhouse gas emissions

a)

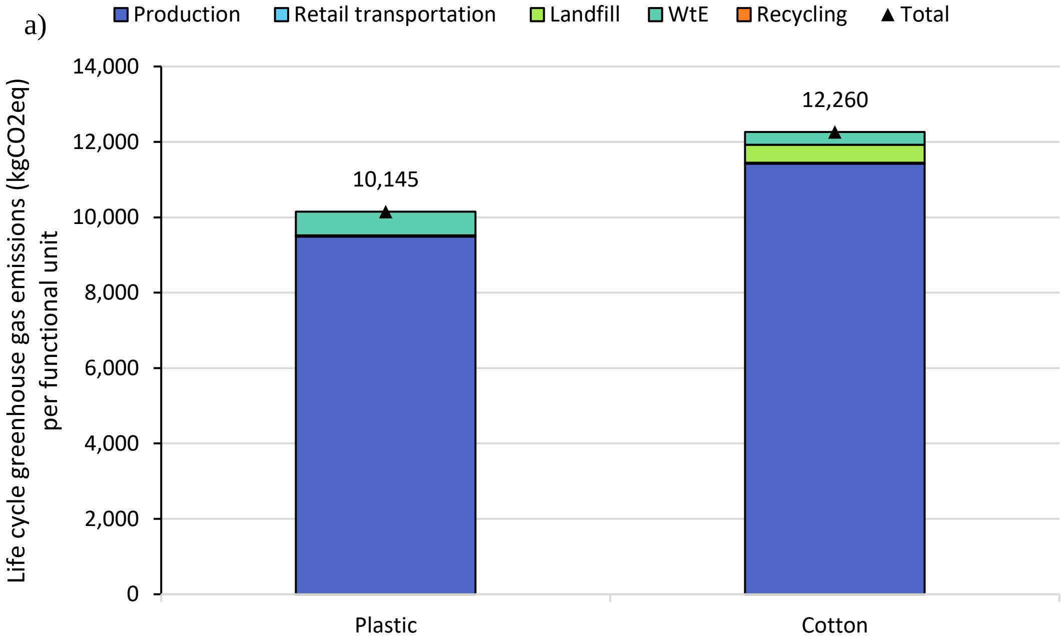

b)

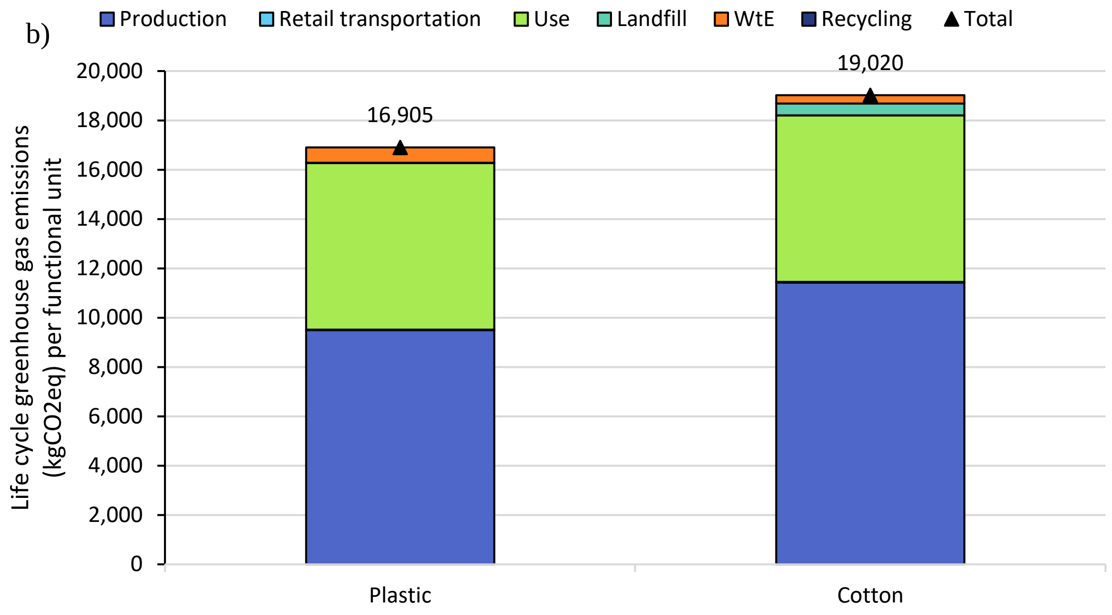

Figure S21 a) Life cycle GHG emissions excluding use phase emissions and b) life cycle GHG emissions including use phase emissions for t-shirts, US 2020 scenario (kgCO_{2eq} per 1,000kg of t-shirts). No emissions for land-use change were considered, since total global area for cotton cultivation has remained constant at 30-35 Mha since 1960 (USDA). GHG emissions from crop rotation, fertilizer, irrigation, pesticide, ginning, packaging, field fuel use, transportation, knitting, knit preparation, batch dyeing, knit finishing, compaction, and cut and sew are included in the production stage.

17 CARPETS

17.1 Functional unit

For a carpet with face weight of 40 oz/square yard and density of 3,100oz/inch

17.2 Raw material acquisition, manufacture and transport

Table S131 Material breakdown for Synthetic (nylon/PET) carpet [1]

Synthetic (nylon/PET) carpet breakdown	Proportion of total weight, %
Face fibre	45
Woven backing	15
Carpet backing adhesive	8
Filler in latex adhesive	32

Table S132 Material breakdown for wool carpet (Source: Expert interview)

Wool carpet breakdown	Proportion of total weight, %
Face fibre	45
Backing, adhesive, and filler	55

Table S133 Carpet, US 2020 scenario: reference flows and EoL disposition

	Reference flow*		EoL pathway (%)		
	Quantity	Weight (kg)	Recycling	WtE	Landfill
Synthetic (nylon/PET) carpet	1 short ton	920	5	0	95
Wool (100% merino) carpet	1 short ton	920	0	0	100

* Functional unit defined by weight as 1 short tone residential, broadloom carpet, since carpet is usually measured by face-weight and nylon, PET and wool have similar densities.

Table S134 GHG emissions for this life cycle stage

	$kgCO_{2eq}$ per functional unit	Source
Synthetic (nylon/PET) carpet	3,723	[1]
Wool carpet	22,789	[1,20]

17.3 Retail transport

Table S135 GHG emissions for this life cycle stage (Source (EPA WARM)

	$kgCO_{2eq}$ per functional unit	Comment

Synthetic (nylon/PET) carpet	27	Average miles per shipment is 497, per EPA WARM
Wool carpet	27	Assume same as synthetic (nylon/PET) carpet

17.4 End-of-life disposition

Table S136 Recycling rates and associated GHG emissions

	Recycling rate, %	$kgCO_{2eq}$ per functional unit	Source
Synthetic (nylon/PET) carpet	5	-93	[21]
Wool carpet	0	0	Expert interview

Table S137 Landfill rates and associated GHG emissions

	Landfill rate, %	$kgCO_{2eq}$ per functional unit	Source	Comment
Synthetic (nylon/PET) carpet	95	19	[21]	Assume non-recycled carpet is all landfilled
Wool carpet	100	20	Expert interview	Assume all wool carpet is landfilled

Table S138 WtE rates and associated GHG emissions

	Incineration/waste-to-energy rate, %	$kgCO_{2eq}$ per functional unit	Source	Comment
Synthetic (nylon/PET) carpet	0	0	[21]	Assume non-recycled carpet is all landfilled
Wool carpet	0	0	Expert interview	

17.5 Use phase/indirect impacts

No measurable indirect impact.

17.6 Life cycle greenhouse gas emissions

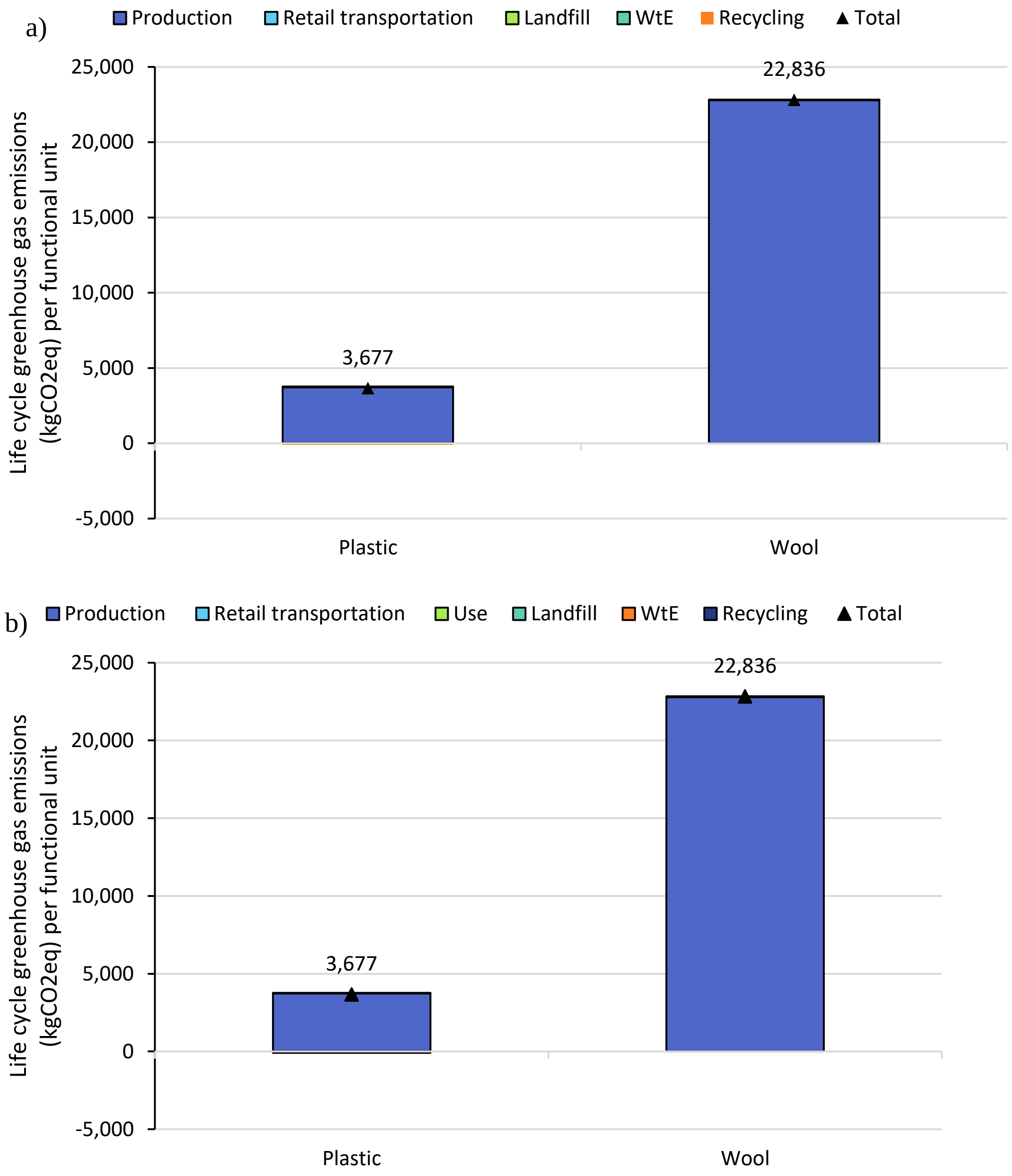

Figure S22 a) Life cycle GHG emissions excluding use phase emissions and b) life cycle GHG emissions including use phase emissions for t-shirts, US 2020 scenario ($kgCO_{2eq}$ per 1 short tone of carpet). Similar durability of fibres over time was assumed.

REFERENCES

(1) US EPA. *Waste Reduction Model (WARM)*; 2022. https://www.epa.gov/warm#:~:text=EPA%20created%20the%20Waste%20Reduction,several%20different%20waste%20management%20practices.

(2) US EPA. *Advancing Sustainable Materials Management: Facts and Figures Report*; 2022. https://www.epa.gov/facts-and-figures-about-materials-waste-and-recycling/advancing-sustainable-materials-management.
(3) I.E.A. *IEA World Energy Outlook*; International Energy Agency: Paris, 2021.
(4) McKinsey. *Global Energy Perspectives*; McKinsey & Company, 2021.
(5) ecoinvent. *The ecoinvent Database Version 3*. https://www.ecoinvent.org/database/database.html (accessed 2021-06-06).
(6) Jeswani, H.; Krüger, C.; Russ, M.; Horlacher, M.; Antony, F.; Hann, S.; Azapagic, A. Life Cycle Environmental Impacts of Chemical Recycling via Pyrolysis of Mixed Plastic Waste in Comparison with Mechanical Recycling and Energy Recovery. *Science of The Total Environment* **2021**, *769*, 144483. https://doi.org/10.1016/j.scitotenv.2020.144483.
(7) UK Environment Agency. *Life Cycle Assessment of Supermarket Carrier Bags: A Review of the Bags Available in 2006*; 2011. https://assets.publishing.service.gov.uk/government/uploads/system/uploads/attachment_data/file/291023/scho0711buan-e-e.pdf (accessed 2022-10-17).
(8) WebstaurantStore. *What Is Butcher Paper?*. WebstaurantStore. https://www.webstaurantstore.com/blog/92/butcher-paper-faqs.html (accessed 2022-10-21).
(9) US EPA. *Plastics: Material-Specific Data*. https://www.epa.gov/facts-and-figures-about-materials-waste-and-recycling/plastics-material-specific-data (accessed 2022-10-18).
(10) MASITEK. *The top 4 breweries in the world use smart in-line sensors*. https://mmaazz.org/industry/beer/ (accessed 2022-10-18).
(11) European Comission. *Environmental Footprint and Material Efficiency Support for Product Policy: Report on Benefits and Impacts/Costs of Options for Different Potential Material Efficiency Requirements for Dishwashers.*; Publications Office: LU, 2015. https://data.europa.eu/doi/10.2788/720546 (accessed 2022-10-18).
(12) Sulistiawati, E.; Astuti, E.; Santosa, I. The Influence of Strong Reduced Water and Fresh Coconut Oil in Viscosity of Liquid Hand Soap. *IOP Conf. Ser.: Mater. Sci. Eng.* **2019**, *543* (1), 012022. https://doi.org/10.1088/1757-899X/543/1/012022.
(13) Sustainable Solutions Corporation. *Life Cycle Assessment of PVC Water and Sewer Pipe and Comparative Sustainability Analysis of Pipe Materials*; 2017. https://accesswater.org/publications/-300020/life-cycle-assessment-of-pvc-water-and-sewer-pipe-and-comparative-sustainability-analysis-of-pipe-materials (accessed 2022-10-17).
(14) Franklin Associates. *Peer-Reviewed Life Cycle Inventory for the Production and Use of Installed Residential Piping Systems for Three House Layouts*; 2011. https://cdn.ymaws.com/www.ppfahome.org/resource/resmgr/pdf/Peer_Reviewed_Pipe_Use_Phase.pdf (accessed 2022-10-17).
(15) SPFA. *Life Cycle Assessment of Spray Polyurethane Foam Insulation for Residential & Commercial Building Applications*; 2020. https://polo14.com/wp-content/uploads/2020/03/SPFA-LCA-Details.pdf.
(16) *On the Road in 2035: Reducing Transportation's Petroleum Consumption and GHG Emissions*; Massachusetts Institute of Technology: Cambridge, Mass, 2008.
(17) EV Database UK. *Compare electric vehicles*. https://ev-database.org/imp/ (accessed 2022-10-18).
(18) van der Velden, N. M.; Patel, M. K.; Vogtländer, J. G. LCA Benchmarking Study on Textiles Made of Cotton, Polyester, Nylon, Acryl, or Elastane. *Int J Life Cycle Assess* **2014**, *19* (2), 331–356. https://doi.org/10.1007/s11367-013-0626-9.
(19) Cotton Incorporated. *LCA Update of Cotton Fiber and Fabric Life Cycle Inventory*; 2016. https://resource.cottoninc.com/LCA/2016-LCA-Full-Report-Update.pdf.

(20) Wiedemann, S. G.; Biggs, L.; Nebel, B.; Bauch, K.; Laitala, K.; Klepp, I. G.; Swan, P. G.; Watson, K. Environmental Impacts Associated with the Production, Use, and End-of-Life of a Woollen Garment. *Int J Life Cycle Assess* **2020**, *25* (8), 1486–1499. https://doi.org/10.1007/s11367-020-01766-0.

(21) Carpet America Recovery Effort (CARE). *CARE 2019 Annual Report*; 2020; p 54. https://carpetrecovery.org/wp-content/uploads/2020/06/CARE-2019-Annual-Report-6-7-20-FINAL-002.pdf.
